# Second Intermediate Period date for the Thera (Santorini) eruption and historical implications

**DOI:** 10.1371/journal.pone.0274835

**Published:** 2022-09-20

**Authors:** Sturt W. Manning

**Affiliations:** 1 Cornell Tree-Ring Laboratory, Department of Classics, Cornell Institute of Archaeology and Material Studies, Cornell University, Ithaca, NY, United States of America; 2 The Cyprus Institute, Nicosia, Cyprus; University at Buffalo - The State University of New York, UNITED STATES

## Abstract

The historical relevance of the Thera (Santorini) volcanic eruption is unclear because of major dating uncertainty. Long placed ~1500 BCE and during the Egyptian New Kingdom (starts ~1565–1540 BCE) by archaeologists, ^14^C pointed to dates ≥50–100 years earlier during the preceding Second Intermediate Period. Several decades of debate have followed with no clear resolution of the problem—despite wide recognition that this uncertainty undermines an ability to synchronize the civilizations of the eastern Mediterranean in the mid-second millennium BCE and write wider history. Recent work permits substantial progress. Volcanic CO_2_ was often blamed for the discrepancy. However, comparison of ^14^C dates directly associated with the eruption from contemporary Aegean contexts—both on and remote from Thera—can now remove this caveat. In turn, using Bayesian analysis, a revised and substantially refined date range for the Thera eruption can be determined, both through the integration of the large ^14^C dataset relevant to the Thera eruption with the local stratigraphic sequence on Thera immediately prior to the eruption, and in conjunction with the wider stratigraphically-defined Aegean archaeological sequence from before to after the eruption. This enables a robust high-resolution dating for the eruption ~1606–1589 BCE (68.3% probability), ~1609–1560 BCE (95.4% probability). This dating clarifies long-disputed synchronizations between Aegean and East Mediterranean cultures, placing the eruption during the earlier and very different Second Intermediate Period with its Canaanite-Levantine dominated world-system. This gives an importantly altered cultural and historical context for the New Palace Period on Crete and the contemporary Shaft Grave era in southern Greece. In addition, the revised dating, and a current absence of southern Aegean chronological data placed soon afterwards, highlights a period of likely devastating regional eruption impact in the earlier-mid 16^th^ century BCE southern Aegean.

## Introduction

Likely the largest global volcanic eruption by volume of material ejected of the last several thousand years [1], and regularly mentioned as a pivotal event in the prehistory of the Aegean and wider East Mediterranean [2–9], the Minoan eruption of the Thera (or Santorini) volcano is in fact best known for a long-running debate over its absolute (calendar) date [4–14]. In relative terms the eruption is placed late in, or at the end of, the Late Minoan (LM) IA cultural period in the southern Aegean (or regional equivalents: Late Cycladic (LC) I and Late Helladic (LH) I). The LMIA period marks the high-point of the Minoan New Palace civilization of Crete [15–19], a time when its major center at Knossos perhaps had a population of 20,000–25,000 people [20], and the Minoan culture including its language (written primarily as untranslated Linear A in this period) was a dominant force in the Aegean [15–18, 21–24]. Little of this is disputed. The controversy surrounds the calendar date of the eruption, and hence the wider cultural-historical placements and synchronizations of the eruption and the associated Aegean archaeological phases versus the cultural phases and history of the eastern Mediterranean (e.g. Egypt, the Levant, Mesopotamia, Cyprus, Anatolia) [4–13, 25, 26].

Before the advent of science-based dating, the chronology of the Aegean Bronze Age was derived from the interpretation of material culture and stylistic linkages between the archaeological record in the Aegean versus contexts dated by the approximate Egyptian historical chronology [9, 25, 27–29]. No clear or undisputed material culture or stylistic linkages for the LMIA period exist. However, the subsequent LMIB and LHIIA periods offered several associations with the reign of the Egyptian king Tuthmosis III. The accession of this king was conventionally placed either ~1504 BCE or ~1479 BCE. As a result, the Thera eruption and the close of the LMIA period was placed ~1500 BCE or ~1480 BCE contemporary with the earlier 18^th^ Dynasty of the Egyptian New Kingdom (NK) [6, 9, 25, 27–30]. A date range of ~1530–1500 BCE remains the ‘conventional’ date for the Thera eruption [6, 8, 25, 27–30]. At a minimum, several authors categorically state that the eruption must be placed after the start of the Egyptian New Kingdom (stated as after ~1550 BCE by [13] citing [30]), e.g. “…that the eruption occurred after the start of the New Kingdom seems in little doubt” ([13] at p. 168). Thus, in an effort to conform to this conventional chronology (and the associated conventional history), dates no earlier than ca. 1560 BCE are sought (e.g. [13] at p.176), and since indeed even this date appears too early for the strict conventional chronology, there is hence also a careful listing of other possible later 16^th^ century BCE suggested possibilities such as 1554, 1548, 1546, 1544 and 1524 BCE ([13] at p.177).

However, then radiocarbon (^14^C) suggested an earlier date range by ≥50–100+ years [7, 9, 31], and so the possibility of a date during the very different Second Intermediate Period (SIP), when a Canaanite dynasty, known as the Hyksos, controlled Lower Egypt and dominated regional interactions [32–35]. This raised (or lent support to) the possibility of a very different cultural history and set of associations in the East Mediterranean and Aegean region [9, 36] (potentially undermining what, until now, have been the very successful efforts of the later New Kingdom state to minimize or remove the Hyksos from history [32–34, 37]). The Hyksos capital city and major port site of Avaris, occupying around 250 ha, was the mega-site of the East Mediterranean at this time [35, 38–40]. The difference in dates and associations thus leaves the Thera eruption on one side or other of a key historical bifurcation ([35] at pp.383-386). The conventional position leaves the majority of the LMIA period and all of the subsequent LMIB period contemporary with and influenced by the New Kingdom Egyptian Empire that expands into southwest Asia from the mid-16^th^ century BCE. In contrast, the earlier date as suggested by ^14^C places all of LMIA and potentially some of LMIB into the time period and cultural influence of the Canaanite Middle Bronze Age world system driven from Avaris. These are two very different contexts and histories (see further below).

**Fig 1:**
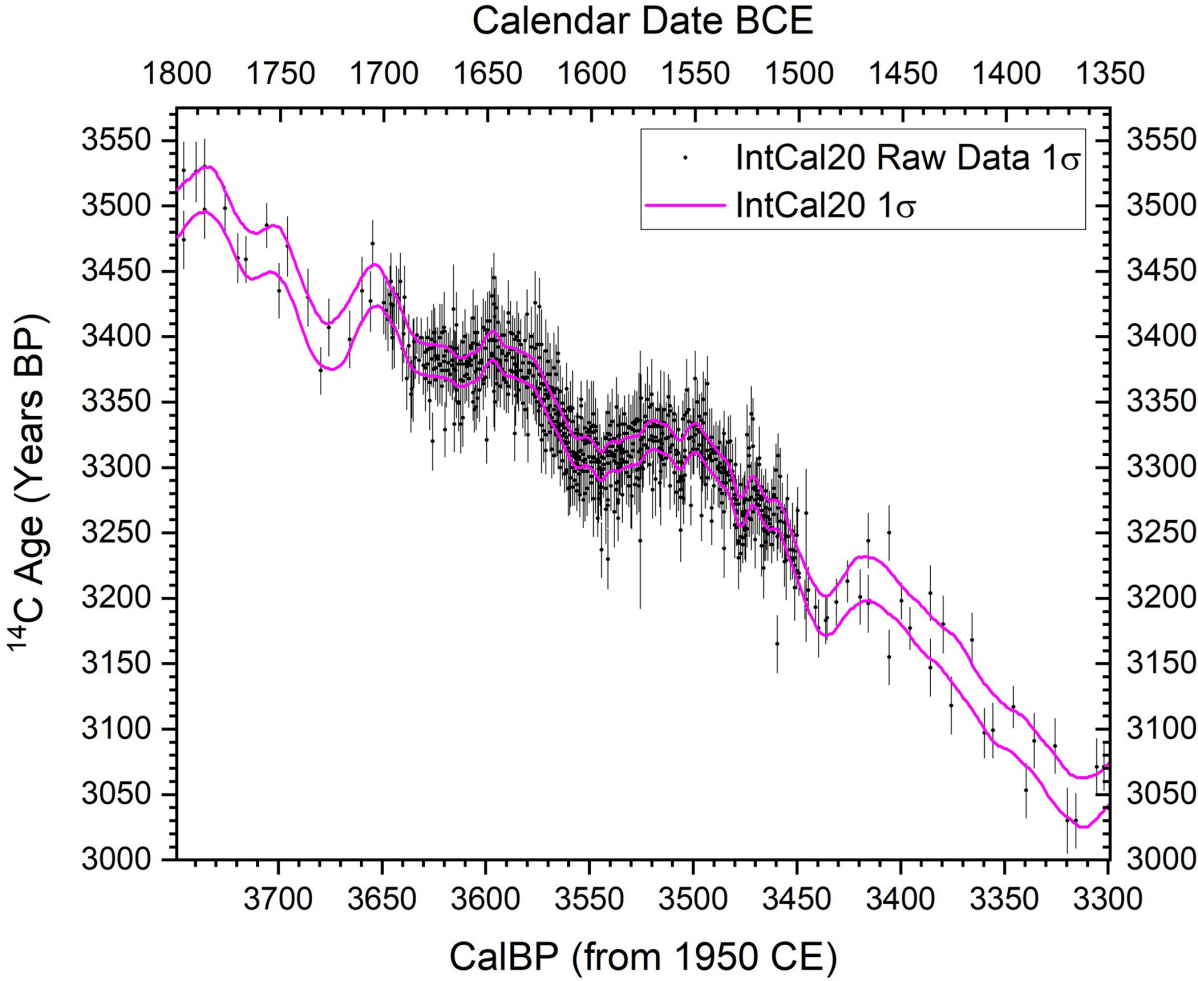
IntCal20 modelled ^14^C calibration curve (1σ) and the raw ^14^C data (1σ) used to compile this curve showing the period 1800–1350 BCE [41]. The notable density of data available for the interval 1700–1500 BCE is evident (most new as part of IntCal20), as also the much better definition that is therefore available for the modelled IntCal20 curve over this period. The constituent IntCal20 dataset is available from: http://intcal.org/.

**Fig 2:**
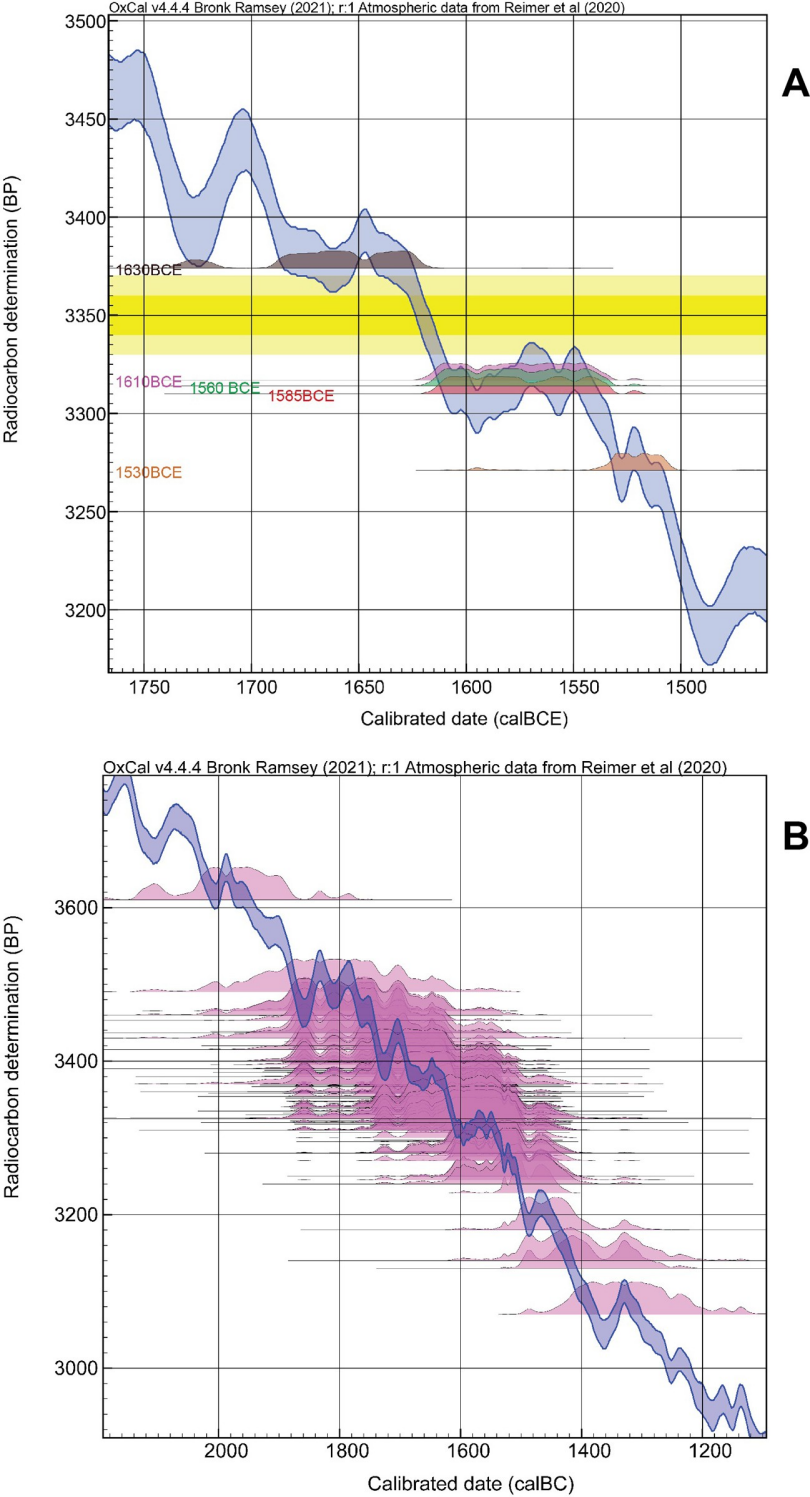
Relationship between ^14^C dating of the Thera eruption and IntCal20 ^14^C calibration curve taphonomy [41]. (**A**) Calibrated calendar age probabilities (no modelling) using OxCal 4.4.4 [57] for ^14^C ages drawn from IntCal20 itself for calendar dates 1630, 1610, 1585, 1560, and 1530 BCE illustrate how ages around especially 1610–1530 BCE spread across the reversal-plateau in the calibration curve ~1620–1540 BCE [41] (see Materials and methods below). The only clarity is that ages ≥1630 BCE or ≤1530 BCE may be distinguished from those in between. A stated average ^14^C age for the Thera eruption [43] is shown at 1σ and 2σ by the yellow bars, intersecting with the calibration curve at multiple places from the early 17^th^ century BCE to mid-16^th^ century BCE. (**B**) The calibrated calendar age probabilities of each of the ^14^C dates in S1A Table (numbers 1–110) employed as relevant to the Thera date showing how the dating probabilities for the vast majority spread across the 17^th^ to 16^th^ centuries BCE given the calibration curve shape.

Resolution of the Thera eruption date is therefore central to the synchronization of civilizations in the eastern Mediterranean in the later Middle Bronze Age (MBA) and early Late Bronze Age (LBA). The current discrepancy has led to rival and largely incompatible ‘high’ and ‘low’ chronologies and different histories [4–13, 25, 26, 28–31]. Recent work has much better defined the ^14^C calibration curve 1700–1500 BCE [12, 41] (Fig 1) reducing the discrepancy and leading to compromise suggestions of a mid to later 16^th^ century BCE date [12, 42]—but even the new data still leave wide ambiguity between potential later 17^th^ century through 16^th^ century BCE ranges [13, 14, 42–45]. The problem is a reversal-plateau in the ^14^C calibration curve that spreads calendar dating probability (Fig 2). The disjuncture and lack of dating clarity undermines wider understanding of the role and impacts of the Thera eruption. To quote one study describing the time of the Thera eruption: “It was summer time, around 1525 B.C. (or was it 1646?)…” ([46] at p.175). While archaeology and geology record the stages of the eruption, its destruction and burial of prehistoric settlements across Thera, its precipitation of devastating tsunamis in the Aegean region, and the covering of a large area of the eastern Mediterranean in tephra [1–4, 9, 13, 17, 45–56], the date is the critical factor for assessing impacts and relevance versus wider archaeology, history and environmental records.

The correct historical narratives, in particular, have been much debated. There are two main topics. First, the subsequent cultural period on Crete after the Thera eruption and the close of the LMIA period is the LMIB period (and its mainland contemporary: LHIIA). The LMIB period on Crete famously ends in a set of well-known destructions at a number of sites [3, 17, 18, 49, 58, 59]. Both archaeology and ^14^C place these close of LMIB destructions in the mid-15^th^ century BCE [3, 5, 8, 9, 18, 25]. The question of whether or not there was an association between the LMIB destructions and the Thera eruption, direct or indirect, has occupied scholarship for over 80 years [3, 17, 18, 49, 58–60]. Second, as already noted, there is the need to elucidate the relationship and synchronization of the enormous Thera volcanic episode with the wider East Mediterranean—for example the approximately historical world of ancient Egypt [4–12, 25–30, 42, 61]. In both cases everything depends on the date. The original ‘low’ date range suggests any eruption impacts are during the earlier 18^th^ Dynasty of Egypt (begins ~1565–1540 BCE) [61–63] and that the eruption was perhaps within 50 years or less of the LMIB destructions and thus potentially linked, even if indirectly [17, 49, 60]. In contrast, the ‘higher’ ^14^C date range places the eruption into the SIP [7, 9, 11, 36, 44, 61–63], when lower Egypt and the Levant were part of a different, culturally mixed, not well-understood, Levantine-Canaanite associated and oriented world-system (in terms of aspects of culture, trade, art, ideas, language) centered at the Hyksos capital of Avaris in the Nile Delta [32–35, 37–40, 64, 65] that dominated the region until the kings of Upper Egypt reconquered all Egypt—sacking Avaris in what is described as “a major rupture and redirecting moment in the history of the Mediterranean” ([35] at p.386)—and going on to create an Egyptian Empire in the Levant. This would be some ≥100 years before the LMIB destructions and it is very difficult, in this case, to see any association between the Thera eruption and the LMIB destructions. There has long seemed no way to overcome the contradictions and impasse between the respective ‘low’ v. ‘high’ chronology positions.

However, recent work and observations prompt and enable a substantial rethink of the topic of the Thera eruption date based on directly relevant ^14^C evidence from the Aegean region. An initial issue is that there has long been one fundamental question mark rendering any ^14^C-based date uncertain or carrying some degree of caveat: are the ^14^C dates from the Minoan eruption volcanic destruction level (VDL) on Thera affected by volcanic carbon dioxide (CO_2_) emissions [4–6, 8, 9, 66–69]? It is argued here that findings reported lately permit resolution of the volcanic CO_2_ question through a comparison of sets of ^14^C dates for the eruption episode from well away from Thera versus the ^14^C dates from Thera itself. It is thus possible to make progress. At the same time, previous assumptions of possible associations of the Thera eruption with volcanic signals observed in ice-cores or with tree-ring growth anomalies have been critiqued and revised [70], and new information is also available [14], all of which highlight the need for critical re-examination of the topic by returning to the available data directly relevant to the Thera eruption. In this paper I argue that the relevance of an important stratigraphically defined temporal sequence in the period immediately before the eruption has not been appreciated and appropriately incorporated into previous dating analyses. By bringing the available data and this observed sequence together via Bayesian chronological modeling, it is possible to determine a better, revised, and refined dating for the Thera eruption.

For the information of readers, I note that the next section, Material and methods, is quite long and detailed. This is because it is (i) important to highlight the sizeable scale and constitution of the datasets used, (ii) essential to recognize and address the possible issues raised in previous scholarship, and (iii) key to explain the novel methods employed in this paper, that, together with the data, enable a distinct (new), much refined, and robust finding to be reported in this study (see Results section). Hence the data and methods are presented here in the main text, providing the necessary details to justify/explain this study and its findings and to allow other **investigators** to **fully replicate this study. Those less interested in materials and methods may wish to skip to the subsequent section: Results.**

## Materials and methods

### Materials

**Fig 3:**
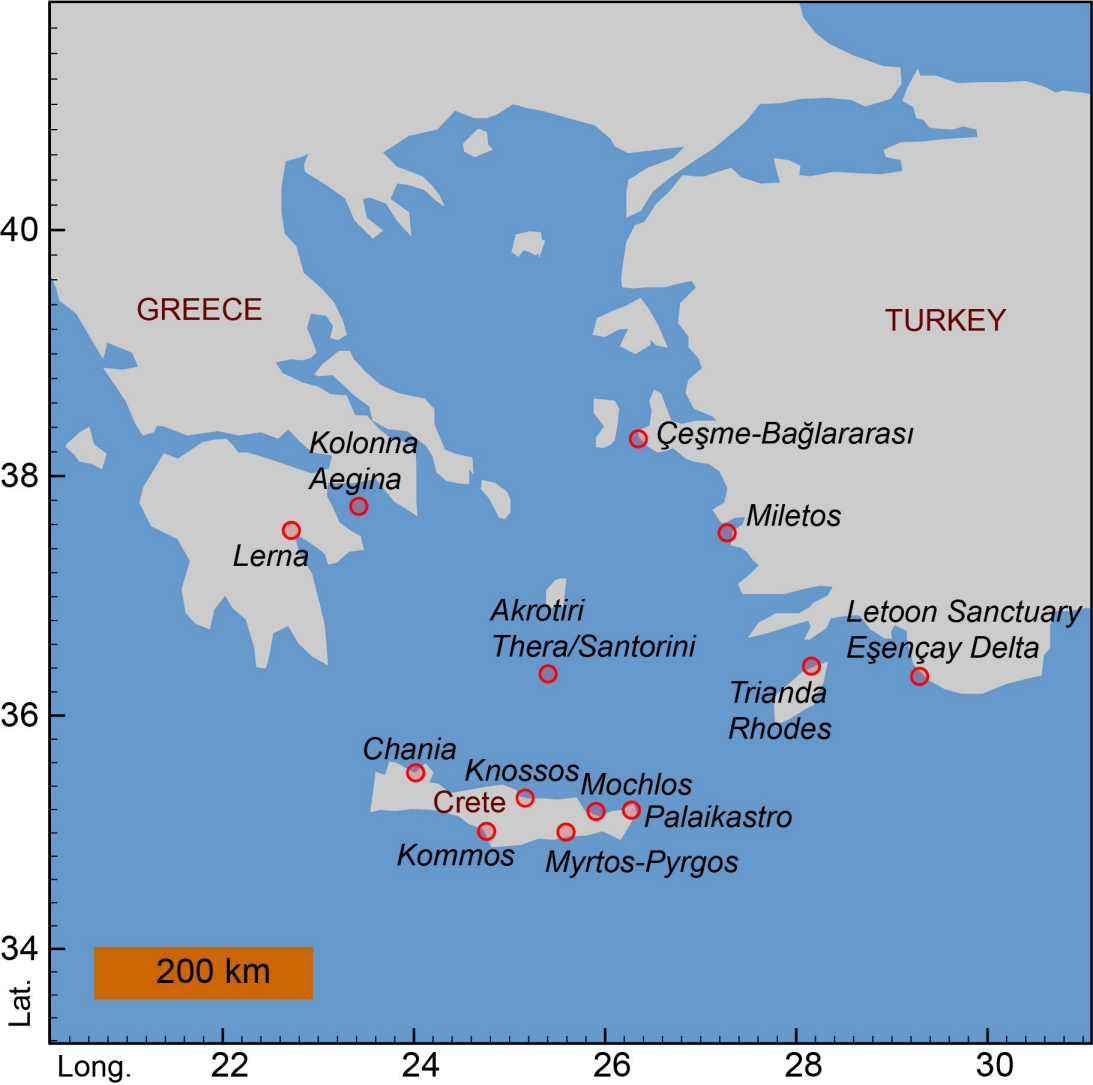
Map showing the locations of the sites in the southern Aegean region providing the ^14^C dates employed in this study. The schematic base map was produced in OxCal 4.4.4 [57] using the open access, publicly available, USGS topo map.

The radiocarbon data employed in this study are all listed, with source reference, in S1A–S1D Table. No permits were required for the described study, since all data employed have been previously published and are publicly available, and no other approvals or regulations apply. Fig 3 shows the locations of the Aegean sites that are the sources of the ^14^C dates used in this study. The datasets used in this study are:

Dataset (a) Samples 1–33 all from non-Thera loci directly associated either with airfall Theran tephra (samples from just before this tephra fall) at Trianda, Rhodes (for archaeological sequence at Trianda, see [71, 72]) or from Thera tsunami contexts at Palaikastro on Crete, Çeşme-Bağlararası, western Turkey and the Letoon Sanctuary, Eşençay Delta, southwestern Turkey [45, 52, 54, 73]. This dataset includes the eruption *terminus post quem* (TPQ) from a tree-ring defined series of ^14^C dates on an oak sample from Miletos in western Turkey [74] that allows a wiggle-match dating for the waney edge (last ring under bark) and so the felling date for this timber which was found buried under Thera tephra (see Bayesian chronological modelling below).

Dataset (b) Samples 34–64 comprising those from secure last VDL (stages (ii)/(iii) or 2/3) or LCI Advanced or Advanced? contexts at Akrotiri from normal processing approaches [7, 67, 74–78] but not including the data from earlier technology and methods produced by the Pennsylvania (P) laboratory (see dataset (d)). This (b) dataset includes a date on insect chitin from a West House VDL (stages (ii)/(iii)) context [79].

Dataset (c) Samples 65–82 on olive branch or root samples from stated, or reasonably assumed to be, Thera eruption pumice contexts [80–82]. The ^14^C dates on a section of an olive branch found likely killed by and buried in the Thera/Santorini Minoan pumice [80, 81] provide a temporal sequence since they were measured on an ordered sequence of inner to outer growth segments. To benefit from this instance of a temporal sequence, the end Boundary of the ordered sequence (the best estimate for the date the sample was likely killed and buried by the eruption: see below in Bayesian chronological modelling) is cross-referenced with the end Boundary of the Phase with the other olive branch/root samples.

**Fig 4:**
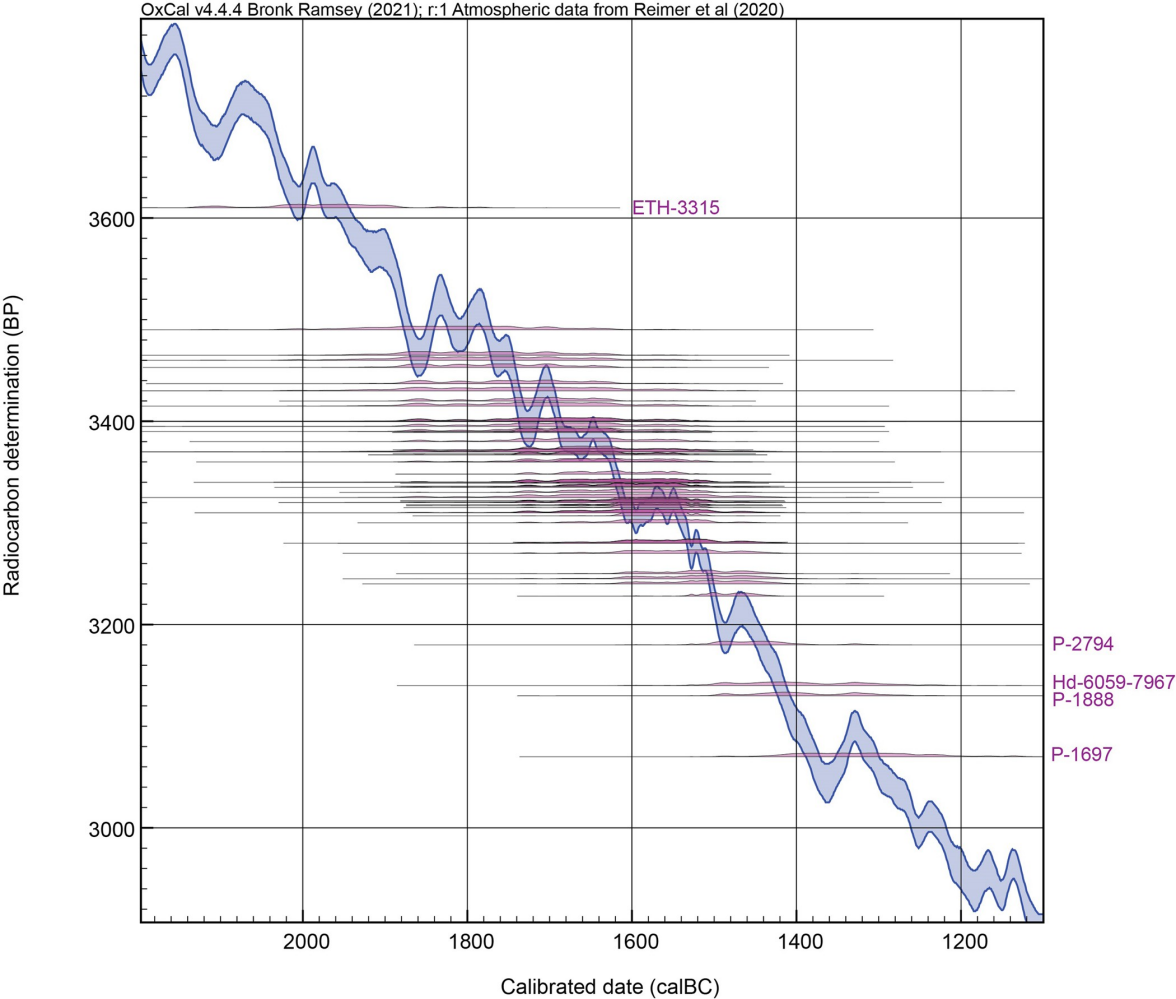
Calibrated calendar probability distributions for each of the dates in dataset (d) from [57] using [41]. As indicated, four of the dates seem visibly too recent; and one date appears substantially too old.

Dataset (d) Samples 34–64 and 83–110 comprising published ^14^C data linked specifically to the VDL (stages (ii)/(iii) or (i)/(ii)–also expressed as stages 2/3 or 1/2) or Thera eruption (Bo pumice-covered) contexts from Akrotiri or elsewhere on Thera (but not the olive wood samples in dataset (c)). This dataset includes the ‘residue’ samples from [67] (both stages (i)/(ii) and (ii)/(iii)), the legacy data from Lamont and from the Pennsylvania Laboratory series I-III work [83–87] that are not either stated as under-sized (small counter: P-1599, P-1619, P-2562, P-2563, P-2564, P-2566) or are clearly very much older than the stated context (unexplained outliers: P-2561, P-2560), as well as the dates on short-lived samples listed from the ETH laboratory [88]. VDL data on long-lived samples (wood charcoal) were not included when apparently rather to very much older than the plausible VDL age and especially when a specific find context was not stated and thus samples might easily relate to earlier MBA first use (e.g. the ETH dates on charcoal reported in [88]). Nonetheless, even with this prior pruning of data, there is still ‘noise’ in dataset (d) based on even a cursory inspection; in particular, four dates yielded ^14^C ages indicating much too recent calendar ages on any mainstream Thera eruption chronology (P-2794, Hd-6059-7967, P-1888, P-1697), and, in reverse, ETH-3315 appears much too old to be compatible with (i.e. relevant to) the VDL (thus 5 of 59 data in dataset (d), or ca. 8% of the data, are likely outliers to some larger extent): see Fig 4. All these major outlier dates except ETH-3315 are pre-AMS technology products, and all were run over 30 years ago. We can therefore anticipate that the outlier model will identify and down-weight these samples in particular (see Bayesian chronological modelling below).

Dataset (e) Dataset (a) re-run with a worst-case Growing Season Related Offset (GSRO) of 4±2 ^14^C years (see section below: Growing season related offsets (GSRO) for ^14^C dates from the southern Aegean?).

Dataset (f) Dataset (b) re-run with a worst-case GSRO of 4±2 ^14^C years

Dataset (g) Dataset (c) re-run with a worst-case GSRO of 4±2 ^14^C years

Dataset (h) Dataset (d) re-run with a worst-case GSRO of 4±2 ^14^C years

Dataset (i) Samples 111–156, and the analytical model used, comprise the Early Helladic to LH ^14^C dataset from the stratigraphically defined temporal sequence at Kolonna on Aegina [89]. These data and Sequence are used in this paper to define the Middle Helladic to LHI transition (start LHI) from the transition between Kolonna Phases J to K. The LHIIIA dates are also employed as a *terminus ante quem* (TAQ) in Model 2 (see Bayesian chronological modelling below). Date VERA-4630, placed as Phase L and dated to the “LHII” period, and so subsequent to the time of the Thera eruption, is used as a TAQ for the Thera eruption (see below).

Dataset (j) Samples 157–165 from early LMIA Kommos, Crete; these offer a temporal sequence to define the date of a short-lived oak twig from an early LMIA context that thus provides a date for a point in early LMIA [74, 90].

Dataset (k-1, k-2) Samples 166–177 from animal bones, parts of either sheep/goat, cow, donkey, deer, pig, from the (single) funerary feast events at mid-LHI Shaft Grave 1 (k-1) and late-LHI Shaft Grave 2 (K-2) at Lerna in southern Greece [91]. The in-built age range incorporated into these animal parts, then consumed as part of each of these two single feasting events, reasonably occupy a maximum total period of ≤15–20 years, since this more than covers the likely maximum age of any of the animals slaughtered, and, in all probability, the time period was rather less (e.g. single digits and likely low single digits) since younger animals will have been preferred for such feasting purposes. Thus we may apply a conservative time constant, a uniform probability of 0–20 years, to the analysis of the period of the Tau_Boundary paired with a Boundary in the OxCal analysis (see Bayesian chronological modelling below) of each feasting Phase. The comparison of the ceramic assemblages suggests that Shaft Grave 1 is mid-LHI and thus likely a little to sometime before the Thera eruption, whereas Shaft Grave 2 is late LHI and thus likely sometime around, or even a little after, the Thera eruption. Datasets k-1 and k-2 are thus not employed in runs of Model 2 (see Bayesian chronological modelling below) as the exact relationships vis à vis the Thera eruption are not stratigraphically-defined, nor definite.

Dataset (l) Samples 178–203 come from three LMIB destruction contexts on Crete, Chania, Myrtos-Pyrgos, Mochlos (close of LMIB destruction at each of the sites), and a subsequent LMII destruction at Knossos [74, 92]. Only ^14^C dates on short-lived samples were used and only dates from AMS ^14^C technology. The Myrtos-Pyrgos destruction in LMIB Late is regarded as before the LMIB Final destruction at Mochlos, following the ceramics-based assessment of Rutter [93]. The LMII destruction at Knossos is a TAQ for all the LMIB destructions.

Dataset (m) Samples 204–257, comprising an additional 54 ^14^C dates from Akrotiri, Thera, Trianda, Rhodes, and Miletos, Turkey which may be specifically related to time periods before or after the Thera eruption (either adding to the evidence for the end MBA/start LMIA period through to the LCI/LMIA period before the Akrotiri VDL, or providing another LMIB dataset [72, 74, 78]. These samples are included in the runs of Model 2 (see Bayesian chronological modelling below). Note: dates derived from older technology radiocarbon dating, and where often lacking proper modern pretreatment requirements and corrections (for isotopic fractionation), or with large measurement errors, or no specific context, are not included in dataset (m)—e.g. dates from the Pennsylvania laboratory (such as those in [84]) are not included in dataset (m).

The data employed are all available in a cited publication (thus the Simon Fraser data [94] are not employed as the measurement data are not publicly available). Data processed and thought to observe a potential older contaminant (Oxford ‘contaminant’ results in [67]) are only employed where stated. Wood-charcoal samples from Akrotiri from recent work were only included in dataset (b) where they were stated as coming from very final pre-eruption LCI Advanced (or Advanced?) contexts [74, 78]. A range of other end MBA/start LMIA through LMIB wood-charcoal samples are included in dataset (m).

### Post-eruption samples setting a TAQ for the Thera eruption date range?

There is a conspicuous absence in work to date of organic samples and ^14^C dates for contexts that are earlier LMIB or mainland equivalent (earlier LHIIA). On Crete, the available ^14^C evidence mainly comes from the close (end) of the LMIB period (see dataset l above), and this appears to be much later (around a century or so) than the Thera eruption (see Results below). Hence these data do not offer a useful (that is an effective) TAQ for the Thera eruption date range. This lack of any ^14^C dates from, and for, earlier LMIB contexts is ever more notable since, contrary initial estimates (e.g. [95]), there is increasing evidence from several sites that the overall span of LMIB was reasonably long, and not very short [92, 93, 96]. However, as evident in a summation of a 2007 workshop on LMIB pottery, recognition of earlier LMIB has proved difficult at many sites, complicated by evidence that earlier LMIB is likely often marked by a continuation of LMIA style (sub-LMIA or so-called Standard Tradition) ([96] at p.51). While some argue, although it is not well or clearly represented, that an LMIB Early phase can be identified at a few sites and represents a time before the appearance of the late LMIB Special Palatial Tradition (e.g. Marine Style), no such early phase has yet been recognized at several other sites ([93, 96] at pp.51-52)). This is a problem, and trying to ^14^C date initial (post-Thera-eruption) early LMIB contexts should be a priority for future work.

The stratigraphically defined series of ^14^C dates from Kolonna on Aegina (dataset i) includes one sample and date, VERA-4630, placed as Phase L and dated to the “LHII” period [89]. This date, whether LHIIA or LHIIB, on a sample of *Ovis/Capra* tibia—thus short/shorter-lived and approximately contemporary with context—should set a TAQ for the Thera eruption. The only small question-mark is that the laboratory notes the collagen yield for this sample was between 1% and 0.5%, and thus a little below the common 1% good/acceptable threshold used to identify reliable ^14^C ages. The date is coherent within the Kolonna Sequence and thus nonetheless appears a reasonable age estimate, but, as the single piece of evidence from this Phase, there is no other direct test/control on the accuracy of this date. Overall, this date appears to offer a reasonable/plausible if imperfect TAQ for the Thera eruption.

A small piece of charcoal found *in situ* in the Pelekita cave on Crete, just above a layer of Thera (Minoan eruption) tephra [97], is another potential piece of evidence. It is stated that:

“It is obvious that the charcoal is anthropogenic in origin, in terms of deposition. Considering its stratigraphic position, the charcoal piece probably reflects renewed usage of the cave by humans after the Minoan Santorini eruption”.

The question of relevance is whether the wood involved (the dated tree-growth increments–the species is not identified) also dates after the eruption or whether these growth increments could be from wood growth before the eruption. In their publication of 2019, Bruins et al. [97] observed that the two ^14^C dates obtained indicated a weighted average ^14^C age, 3365±28 years BP, a little older than the weighted average value from Thera eruption datasets (e.g. the value of 3350±10 ^14^C years BP stated in [43]—see Fig 2). Thus these authors suggested the charcoal possibly “consists of wood that already grew before the eruption, but was used by people to make a fire in the cave after the eruption (old-wood effect)”. However, with the publication of IntCal20, this concern might be reconsidered. The raw dataset behind IntCal20 suggests a number of measurements from the 16^th^ century BCE with similar ^14^C ages to those determined from the Pelekita charcoal (see Fig 1). Thus the sample could indicate wood that grew after the eruption. However, the lack of species identification or any other information limits any further analytical potential. We are left with multiple possible scenarios and no clarity. The wood used in the cave could have been residual (a dead branch, etc., collected on the ground and then used by humans), or inner (older) tree-rings of a piece of wood now used; just as it could also have been part of a shorter-lived branch collected and now used and so provide a good TAQ for the eruption. The problem is that we simply cannot determine which scenario is correct. Therefore, it appears inappropriate to regard this Pelekita sample as providing any useful TAQ information; hence it is not used.

Some dates from Tsoungiza from “LHI-II” contexts were published in [74]. Whether the LHI-II context involved necessarily post-dates the Thera eruption is not certain. Moreover, this set of six dates from Tsoungiza is clearly problematic [74]. The three dates from LHI-II contexts all offer rather older ^14^C ages than the three from earlier LHI late contexts, contrary expectations, and two of the three LHI late dates on charcoal (OxA-11312, OxA-11314) are conspicuously too recent for LHI on any plausible (high or low) chronology. It therefore appears impossible to regard any of these dates as necessarily offering an appropriate, let alone reliable, TAQ for the Thera eruption.

Finally, there are a few LMIB or LHII ^14^C dates from older pre-AMS ^14^C work [84, 98], mainly from the Pennsylvania (P) laboratory. Many of these samples lack the requirements of modern pretreatment standards, and correction for isotopic fractionation. Two dates from Myrtos-Pyrgos on Crete are on the same short-lived material from the close of site LMIB destruction dated by subsequent AMS ^14^C in dataset (l) [74, 92, 99]. It seems preferable to use the more recent AMS values (although the P values are not inconsistent). The other samples from LMIB contexts at Myrtos-Pyrgos (P-2115, P-2116, P-2343, P-2344A [86]) are all on unidentified wood charcoal and, while one or more might relate to LMIB use of wood with only modest in-built age (e.g. P-2115?), the others with older ^14^C ages may well either include more substantial in-built age or in fact relate to building activities at the site in early LMIA after a Middle Minoan III destruction, and thus have no relevance to the date of LMIB. We simply cannot offer any secure association and no clear TAQ for the Thera eruption. Hence it seems inappropriate to use these data. Two dates from Palaikastro published in 1965 (St-1263, St-1264 [100]) on wood charcoal are much older than plausible for LMIB (notwithstanding large measurement errors) and are either cases of dating samples of old-wood (in-built age) or residual material. Again, it is inappropriate to use these data. There are also some dates from Ayia Irini on Keos (P-1282, P-1283, P-1284, published 1969 [101], and P-2576, P-2579 published 1978 [86]). All are on charcoal with no further information on species or samples. Two of the ages obtained are much too recent on any chronology (P-1282, P-1283). The others come from Period VII contexts at the site, these are placed as LHIIA contemporary with LMIB [102]. These site contexts should offer a TAQ for the Thera eruption. So might these samples, but this is subject to an unknown caveat concerning possible in-built age since they are unidentified wood charcoal samples. A critical assessment would again have to rule them out as secure TAQ evidence. There are a few other dates from LMIB or LMIB? or equivalent LHII contexts, but all are on wood charcoal, and the potential in-built age caveat applies, even if the find context is secure, and in several cases these dates, from now historic measurement processes, appear aberrant (e.g. far too recent like P-1356 at 2964±74 ^14^C years BP [101]), or are later than other close of LMIB AMS ^14^C dates in dataset (l) and hence not useful as closer TAQ evidence than the data already available (e.g. P-2717 “probably of LMIB date” from Plagiada, Crete [87]), and hence the only decision is to regard all these old-technology dates as inappropriate or irrelevant for use.

At present, this leaves VERA-4630 from Kolonna as the only recent AMS ^14^C age of relevance as a close TAQ for the Thera eruption. The modelled posterior probability for this ^14^C measurement from the separate run of the dataset (i) model is thus employed as a TAQ in the initial Model 1 run (see Results). An obvious question is whether VERA-4630 has a major effect on the date determined for the Thera eruption? The answer is no. It acts only to slightly restrict the spread of probability on the late side (see Results). It is not decisive in identifying a most likely date range for the Thera eruption around the end of the 17^th^ century or early 16^th^ century BCE. In Model 1 VERA-4630 acts as a TAQ for the whole Sequence to that point. In Model 2 the probability for VERA-4630 is cross-referenced in as a TAQ for the Thera Eruption Boundary. Model runs without VERA-4630 are also considered for comparison (see Results) and to show that the inclusion of VERA-4630 only slightly restricts the late part of the dating range for the Thera eruption and is not decisive.

### Temporal sequence on Thera immediately leading to the eruption

The Thera volcanic eruption occurred in the later spring-summer [79, 103] towards the end of the LMIA or LCI or LHI cultural periods in the southern Aegean destroying a large and, until then, thriving cosmopolitan port city at Akrotiri on Thera [17, 46, 50, 51]. Excavation and study at Akrotiri has identified a temporal sequence of stages in the period immediately leading to the eruption: (i) major earthquake, (ii) systematic clearance/repair works, (iii) abandonment of the town—presumably because of first signs of volcanic activity or renewed seismicity—removing most portable valuables but leaving stored foodstuffs and other materials, carefully secured, behind, (iv) subsequent precursory eruption(s) leaving four thin pumice layers, Bo_0_ layers 1–4 [48], with evidence of some gap (period of time, days to months?) between Bo_0_ layers 3 and 4 given evidence that people returned to the site starting clearance and some repairs, and (v) the massive Bo_1-4_ Minoan eruption [2, 46–48, 51, 104]. Some evidence of humus and colonization of soil above the seismic debris of stage (i) by plants likely indicates “a period of several years” [104] before stage (iv), but the overall timespan, stages (i) to (v), is variously considered relatively brief (between months, a season, to a few/several years) [3, 17, 46, 48, 49, 51, 104, 105].

### Volcanic CO_2_ and resolving this issue for the Thera case

A potential volcanic CO_2_ effect (release and incorporation of old, depleted, CO_2_) could inflate ^14^C ages [66–69, 106–109]. While usually more associated with ground-level plants and shrubs near an emission source (few hundred meters), effects of large, strong volcanic CO_2_ releases have been observed in proximate tree leaves and tree-rings [106, 108–110]. Instances of substantive effects, that is ≥100s to 1000s of ^14^C years, are usually observed in low and ground-level plants or plants/trees growing within a short distance of an emission source, such as the instances from the Eifel area, Germany, and Thera [106], or those from the Furnas caldera, Azores, within just a few hundred meters of the vent [107], or in leaves and tree-rings in or close to the heavy emission loaded areas at Mammoth Mountain and Yellowstone in the USA [108, 109]—e.g. in the Yellowstone case “a mature, 16-m-tall lodgepole pine located 60m from nearest hydrothermal feature (Mud Geyser) but surrounded by 8 major hydrothermal features within a 300m radius” [109]). In contrast, no instances of apparent substantive (larger) offsets or differences (to older ^14^C ages) seem apparent when reviewing the set of Theran VDL ^14^C samples. The Thera VDL dataset instead comprises ^14^C ages broadly similar with the ^14^C dates achieved on similar material from other approximately contemporary Aegean contexts [4, 5, 7, 9, 67, 70, 74]. No volcanic CO_2_ effect is evident. This is not a surprise. It may be noted that evidence for volcanic CO_2_-caused ^14^C-aging in tree-rings, even when the trees grew close to a degassing locus, can be indistinct or ambiguous [111], and, examining the Azores case in detail, there was a zero-age effect for the control site, PG, located outside the caldera and only some 1km from the indicated fumaroles [107]. Each such case clearly varies with very different histories and circumstances and scales of emissions evident (and claims to identify such effects can be controversial [112, 113]). The indications of relatively rapid magma chamber assembly for the Thera volcano (as also Taupo) [114], contrast longer drawn-out processes, may be relevant in minimizing any longer-term (beyond the period of the eruption itself) evidence for volcanic CO_2_ effects on vegetation as relevant to the available samples and contexts investigated for the dating of the Minoan eruption. Nonetheless, at high-precision, and because of the location of the Thera eruption timeframe around a reversal/plateau in the ^14^C calibration curve, even quite small ^14^C effects could nevertheless substantially affect calendar dates [12, 13, 44] (see Fig 2). Thus: can we resolve whether or not any relevant volcanic CO_2_ effect applies in the specific Thera case?

Resolution of this long-running problem is only possible through comparison of (i) ^14^C data from the VDL on Thera versus (ii) ^14^C dates from closely contemporary contexts well away from Thera and distinct from any possible volcanic CO_2_ influence. This comparison enables us to achieve both an eruption date free from any possible volcanic CO_2_ influence, and quantification of any possible offset relevant to samples from Thera itself. Organic samples associated with Thera eruption tsunami and airfall tephra deposits at loci well away (>200km) from Thera (northeast at Çeşme-Bağlararası, western Turkey, east in the Letoon Sanctuary, Eşençay Delta, southwestern Turkey, southeast at Palaikastro, Crete, and east at Trianda on Rhodes) (Fig 3) [3, 45, 52, 54, 74], with no plausible volcanic CO_2_ input, enable substantial progress. We can compare ^14^C dates on organic samples from these contexts, stage (v) in terms of the Akrotiri stratigraphic sequence (see above), versus those on olive tree elements likely killed and buried by the main eruption on Thera (also dating stage (v) in terms of Akrotiri), and versus those from the preceding final use and abandonment stages (ii)/(iii) at Akrotiri, in order to address and resolve the question of volcanic CO_2_, while in addition clarifying the timing between stages (ii)/(iii) and (v) (see below: Bayesian chronological modelling).

### Growing season related offsets (GSRO) for ^14^C dates from the southern Aegean?

**Fig 5:**
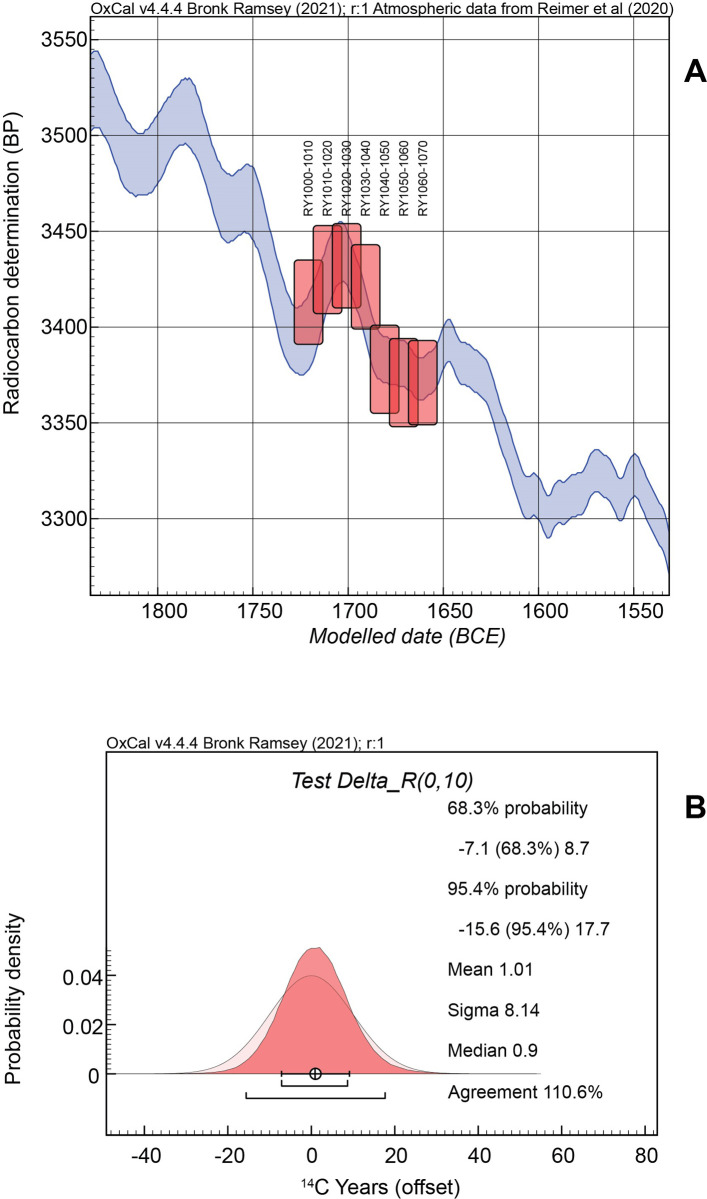
Results of a neutral (0 ± 10 ^14^C years) Delta_R test for a growing season related offset (GSRO) for the tree-ring defined time-series from an oak sample from LMIA Miletos, western Anatolia. **(A)** The seven weighted average ^14^C values [132] for 11 tree-rings (years) samples, Relative Years (RY)1000-1010 … to RY1060-1070, each spaced (mid-points of each block) 10 rings = calendar years apart (from pairs of ^14^C data in each case: see S1A Table nos. 20–33) show a good, ordered, fit against the IntCal20 ^14^C calibration curve [41]. The IntCal20 curve is shown as a 68.3% highest probability band and the Miletos samples are shown with the boxes illustrating, y axis, the weighted average ^14^C ages plus or minus 1σ, and, x-axis, the 68.3% highest posterior density (hpd) calendar placement of each element of the time-series. **(B)** The Delta_R test indicates a ~0 to very negligible offset (mean difference: 1.01 ± 8.14 ^14^C years) versus the 0 ± 10 ^14^C years prior (see below: Bayesian chronological modelling).

This issue may apply if there is a sufficient difference (or offset) in growing season between (i) a dated sample and (ii) the growing season of the trees used to derive the relevant portion of the northern hemisphere IntCal ^14^C curve such that the dated sample (i) records a differing portion of the intra-annual (seasonal) atmospheric ^14^C cycle [44, 115–117]. Thus the question is whether the relevant plant material from the southern Aegean has a sufficiently different growing season as to be different in measurement terms versus the tree-rings and their growing seasons used to provide the ^14^C values for the IntCal calibration curve (trees from central-northern Europe and high elevation SW USA for the relevant period 1700–1500 BCE) [41, 44, 115–117]. In the eastern Mediterranean/Near East the issue is an earlier, primarily autumn-spring, versus spring-summer, growing season for many field crops (note grapes and olive fruit have later growing seasons). In extreme cases in the literature related to Egypt and the southern Levant (with harvest of most field crops by April-May), and when comparing comparable modern accelerator mass spectrometry (AMS) ^14^C dates for both sample and calibration curve, the difference seems to be of the order of ~12±5 ^14^C years [44, 117]. Comparison of bomb-period data also suggests similar underlying atmospheric ^14^CO_2_ differences of around 1‰ or 8 ^14^C years (Rehovot, Israel versus Vermunt, Austria in 1967–1968 [118, 119]) and these small differences merge also into observations of small (e.g. ~1‰ or 8 ^14^C years) latitudinal differences [120–122]. Given an intra-annual seasonal cycle of atmospheric ^14^CO_2_ levels from a late winter low to a later summer high [122–125], the effect is to increase ^14^C ages slightly versus the calibration curve. However, in the central to southern Aegean case, with field crops typically planted late Autumn and with harvest May-June to start of July [126], the offset will be rather less. The exact possible variation will vary by species (and area, specific weather conditions, and farmer [126]). For example, in a study on the island of Amorgos, relatively proximate to Thera, harvest (so a time beyond any further growth) of the typical and preferred autumn-winter sown field crops “started in late May with pulses and continued through June with cereals–barley before wheat and finally oats” ([126] at p.71). Standard modern (variously 1996–2008) timings for flowering and then maturity of early or late Autumn planted wheat and barley (10 November or 30 November) for Crete are modelled presently to give dates (day of year) of days 56–142 and 79–155 for wheat and days 83–147 and 103–160 for barley [127]—hence respectively dates in March to June and late March to mid-April to June. In contrast, these crops are harvested in traditional/ancient circumstances from April-May in Jordan, Israel and Egypt [126, 128, 129]. The growing season for central and northern European oak, which comprises the majority of the evidence for the relevant section of IntCal20 [41], is late April/early May to late August/mid-September [117, 130]. The other tree species providing data for the period 1700–1500 BCE in IntCal20 is bristlecone pine, with a mid to late June until late July or early August growing season [12]. Thus the overlap of the grain/seed development period in the southern Levant-Egypt case is around 0 to 0.5 to at most 1 month. In contrast, it is around 2 months for Crete versus the oak. Hence an estimate of a typical difference of around (0 to)25-33% of the Egypt-southern Levant offset appears appropriate, with the worst case, rounded up, as ~4±2 ^14^C years. The real expectation would be less to effectively 0—as indicated also by a neutral 0±10 ^14^C years Delta_R test comparing the Miletos oak tree-ring-defined ^14^C series versus IntCal20 (Fig 5) that yielded a negligible offset (quoting the mean ± σ) of ~1.01 ± 8.14 ^14^C years. Hence, overall, the relevance of a GSRO should be small to negligible for the southern Aegean case. And, indeed, some other researchers do not consider such small growing related seasonal offsets to be either real or substantive or relevant [131]. The potential maximum, or ‘worst-case’, relevance of the southern Aegean GSRO is therefore noted in a few instances in the main text, but, in general, it is likely that the calendar ages derived from IntCal20 are probably reasonably representative for the southern Aegean. Inevitably the addition of the GRSO factor, and especially its additional error component, creates slightly greater variation in realizations from modelled results, and especially at the recent end where the plateau in the ^14^C calibration curve lacks diagnostic discrimination (see the examples in Results below).

### Bayesian chronological modelling

Bayesian chronological modelling is employed to analyze the ^14^C data, and, in particular, to incorporate known prior information, such as stratigraphically-defined order [57, 133–136] and to include, assess, or address the additional issues discussed above. Analysis of the ^14^C data employs the northern hemisphere IntCal20 ^14^C calibration curve [41] with Bayesian chronological modelling using the OxCal software [57] version 4.4.4 with curve resolution set at 1 year. For the OxCal manual, see: https://c14.arch.ox.ac.uk/oxcalhelp/hlp_contents.html. OxCal Chronological Query Language (CQL2) command terms, like Boundary, Date, D_Sequence, Delta_R, Difference, Phase, R_Combine, Sequence, Tau_Boundary, etc., are capitalized in the text. Data with some association (i.e., not entirely independent), modelled within Phases or Sequences in OxCal [57, 136], are modelled within Boundaries to avoid a non-realistic wider spread of probability [137]. Since it is known that several of the datasets involved likely lie on a period of a reversal-plateau in the ^14^C calibration curve (Fig 2), some issues of ambiguity are anticipated. Hence a much increased (x100 from the OxCal default) kIterations value of kIterations = 3000 was routinely employed to be conservative and to try to ensure stable results with good convergence for each model run. In the case of the Model 2 runs, a kIterations value of 300 was also employed since this large, complex, model (especially the GSRO version) will not always complete (or within any reasonable time) using the online version of OxCal with kIterations set at 3000. Thus, in order to allow full, accessible, testing/replication of the findings reported, a model version that does run satisfactorily with the online version of OxCal within a reasonable timeframe (versus running on an independent machine) was also employed (kIterations = 300). Only model runs with all elements with Convergence values ≥95 were used (at end of run or >20M iterations, most cases, or once >12M iterations completed for some Model 2 cases). The IntCal20 calibration curve includes several hundred recent ^14^C measurements on known-age tree-rings for the calendar interval 1700–1500 BCE, due to the widespread recent interest in trying to define this period and the Thera eruption date (since [12]), making this two-century interval by far the best-defined portion of the IntCal20 ^14^C calibration curve in the BCE era [41, 43] (Fig 1). This important revision of the IntCal dataset (IntCal20) refines the calibration context for the Thera period—versus past work that employed earlier iterations of the IntCal calibration curve (e.g. IntCal13 and previous versions) [12, 43, 44]—and makes findings much more robust. The OxCal General Outlier model is applied to dates on short or shorter-lived samples (including some instances of wood-charcoal dates where it is evident any in-built age factor must be consistently very minor to negligible: see below) and for combinations of such dates (unless in a tree-ring sequenced wiggle-match) to detect and then down-weight outliers proportionally [138]. Dates on wood-charcoal where some in-built age cases are likely are employed with the OxCal Charcoal Outlier model applied to approximately reduce the likely in-built age element [138]. In cases where dates were run on the identical sample material, these were combined using the OxCal R_Combine function as long as they passed a Chi-square test indicating the measurements were consistent with this assumption [132]. Dates within an R_Combine (or Combine) function, or dates forming part of a tree-ring defined wiggle-match [139, 140], were assessed and down-weighted as outliers using the OxCal SSimple Outlier model [138].

Most of the data employed belong to a set of dates from a context (a Phase in OxCal) that ends with a specific event, e.g. the stages (ii)/(iii) abandonment at Akrotiri, the stage (v) Thera eruption/tsunamis, a feasting event, a site LMIB destruction, etc. The aim of the analysis is to estimate the date of this end event. In these cases the data should all provide ages before the final/closing event. Most data, and especially those on short/shorter-lived samples, are likely immediately or very shortly before the event, but some may be a little older (e.g. storage, or because of a little in-built age, or because they are residual). Plant materials were likely only stored for short periods of a couple, to at most a few, years given evidence of available ancient-traditional practice in contexts like those relevant to the southern Aegean LMI-II sites [126, 141, 142]. Animal bones, whether sheep, goat, cow, pig (etc.), may incorporate one to a few years of age before slaughter and use as food (when found disarticulated), to a few to several years if kept until older age/death [143], and any directly killed by the tsunami could be in either category. A few samples may be even rather older (e.g. in-built age in the case of wood-charcoal if not outermost tree-rings, or residual material from earlier activities in the area). Altogether, in the case of a set of such data from a defined event (destruction, or a specific use event like a funerary feast), the data thus likely form an exponential distribution ramped to immediately before the destruction/final activity event of interest. To represent this situation the data in such a Phase are modelled within a Sequence using a Tau_Boundary paired with a Boundary in OxCal, so that that an assumed exponential distribution of data best describes the event of interest as the end Boundary for the Phase [57]. Such a modelling approach has the advantage of ensuring that older dates on individual samples, whether residual or because of some in-built age, do not lead to overestimation of the age of the eruption event (contrast use of an average value for the set). Where data or Phases of data have a known stratigraphic or agreed material culture-based temporal order, they are modelled within an OxCal Sequence that incorporates this prior knowledge.

Comparison of the posterior probability distributions describing the end Boundaries for different datasets, e.g. the ^14^C data on samples from Thera eruption/tsunami contexts away from Thera/Santorini (dataset a) versus those from stages (ii)/(iii) contexts on Thera/Santorini (dataset b) or those on olive material from the eruption pumice on Thera (dataset c), use the OxCal Difference query to quantify the temporal difference between them expressed in calendar years. The Difference probabilities between the two compared probability distributions are illustrated and described in terms of 68.3% and 95.4% probability ranges, and as the differences between the mean (μ) or median (M) of the respective Difference probability distributions (see Results). Quantification of the existence of, or effect of, an offset in ^14^C ages between a dataset and the IntCal20 calibration curve, for example because of a growing season related offset (GSRO), see above, uses the OxCal Delta_R command [138]. In the former case a neutral prior of 0±10 ^14^C years is used to test for the existence of a potential offset; in the latter case the effect of the likely maximum GSRO for southern Aegean samples is tested by use of a likely worst-case 4±2 ^14^C years offset (see above).

**Fig 6:**
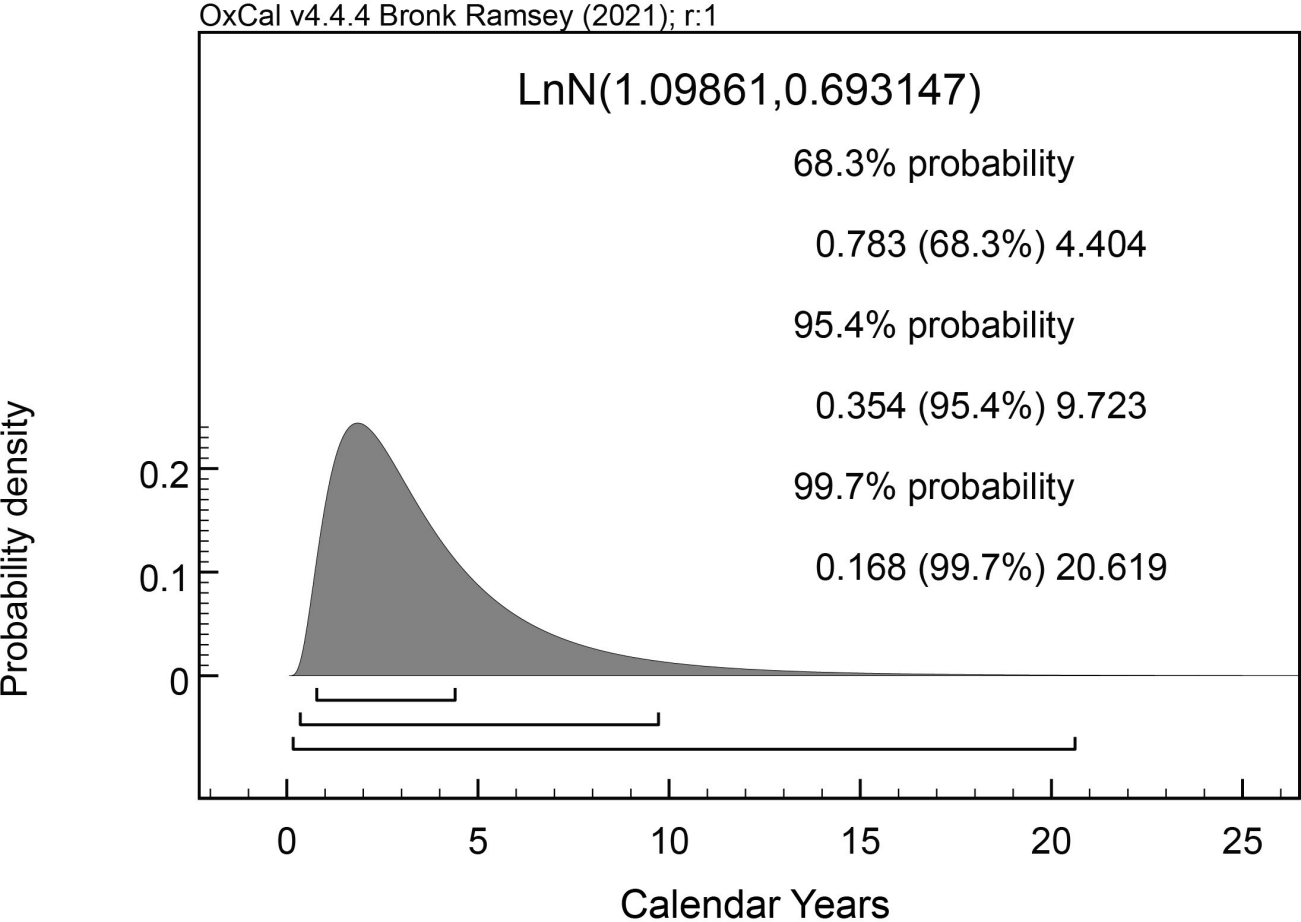
The prior probability distribution from the LnN(ln(3),ln(2)) constraint applied to the difference query in the OxCal [57] models discussed below (see Results).

Archaeological and geological observations propose that the total timespan between stages (ii)/(iii) to (v) at Akrotiri on Thera represents a relatively short period of time (see above). Estimates vary from a timespan of weeks/months/season(s) [3, 17, 46, 48, 49, 51, 104] up to “a period of several years” [105]. A plausible extreme maximum allowance comes from the comparison of the abandonment/eruption date ranges from datasets (a) versus (b) (see Results below). Here, with no additional constraints applied, and with probability allowed to spread across the reversal-plateau in the ^14^C calibration curve from the late 17^th^ through mid-16^th^ centuries BCE, the comparison of differences between dataset (a) versus (b) suggests (from the mean/medians, μ/M) a likely total maximum difference of the order of <15 years. To implement the expected timespan limit, a constraint was placed on an OxCal Difference query applied to the period of time between the Boundaries determined for the end of stages (ii)/(iii) and stage (v) in the OxCal model. An appropriate strategy for this constraint appears to be a log-normal distribution with most probability for a very short interval (months to several years) but with some decreasing probability for a longer interval with no hard boundary (since this actual period is not known, only estimated), such that the data can overwhelm the prior assumption if this assumption proves to be inappropriate. A log-normal distribution with a mode around 2 years and a standard deviation giving a 68.3% range from <1 year to ≤5 years and a 95.4% range from around <0.5 year to around 10 years appears appropriate given the stated expert expectations. This was implemented using OxCal code of the form:

Difference("D","TE5","E2/3",LnN(ln(3),ln(2)));

Where in this example (as used in Model 1 see below) the Difference “D” is the time between the “TE5” Boundary (stage v) for the pooled datasets (a) + (c) (Thera Eruption) and E2/3 which is the End (E) Boundary for the Phase with the Akrotiri stages (ii)/(iii) dataset (a), and this Difference has the LnN(ln(3),ln(2)) constraint applied to it. The form of this LnN(ln(3),ln(2)) prior probability distribution is illustrated in Fig 6. In the Model 1 runs, shown below in Results, the available data and modelled assumptions correspond well (see below). As an alternative, use of a uniform probability (U) constraint of 0–15 years was also tried (see Results below). Thus:

Difference("D","TE5","E2/3",U(0,15));

Both approaches yield very similar results.

Two time-ordered series are included in both Model 1 and Model 2 (see Results). The first series comprises ^14^C dates on a defined tree-ring (oak, *Quercus* sp.) sequence of 72 tree-rings ending in waney edge (ring immediately below bark) from Miletos from a sample that was found buried beneath Theran tephra fall [74, 140, 144]. The OxCal D_Sequence function [139] is employed to undertake a ‘wiggle-match’ and to quantify the dating probability for the waney edge (and so the TPQ for the Thera eruption provided by this sample). The OxCal SSimple outlier model is used to test for and to down-weight outliers within the wiggle-match [138]. This waney edge TPQ date estimate is then included in the non-Thera/non-Santorini dating set as an element in the Phase of data. The second series is from an olive (*Olea europaea*) branch section where ^14^C dates are available from an ordered sequence from inner to outer growth segments [80, 81]. However, since it is difficult (to impossible) to determine annual growth increments (tree-rings) in olive via visual identification [145], this series is analyzed as a Sequence in OxCal, inside start and end Boundaries, with the assumption of the order but with no information about supposed ring-spacing included [146]. The end Boundary for the Sequence is regarded as the estimate for the date of the outermost growth increment of the original branch/tree and so the date for, or very close TPQ for, the Thera eruption (that likely killed and buried the tree [80, 81, 146]), and, in order to incorporate the known temporal sequence information, this age is incorporated via a cross-reference with the end of dataset Boundary for dataset (c).

**Fig 7:**
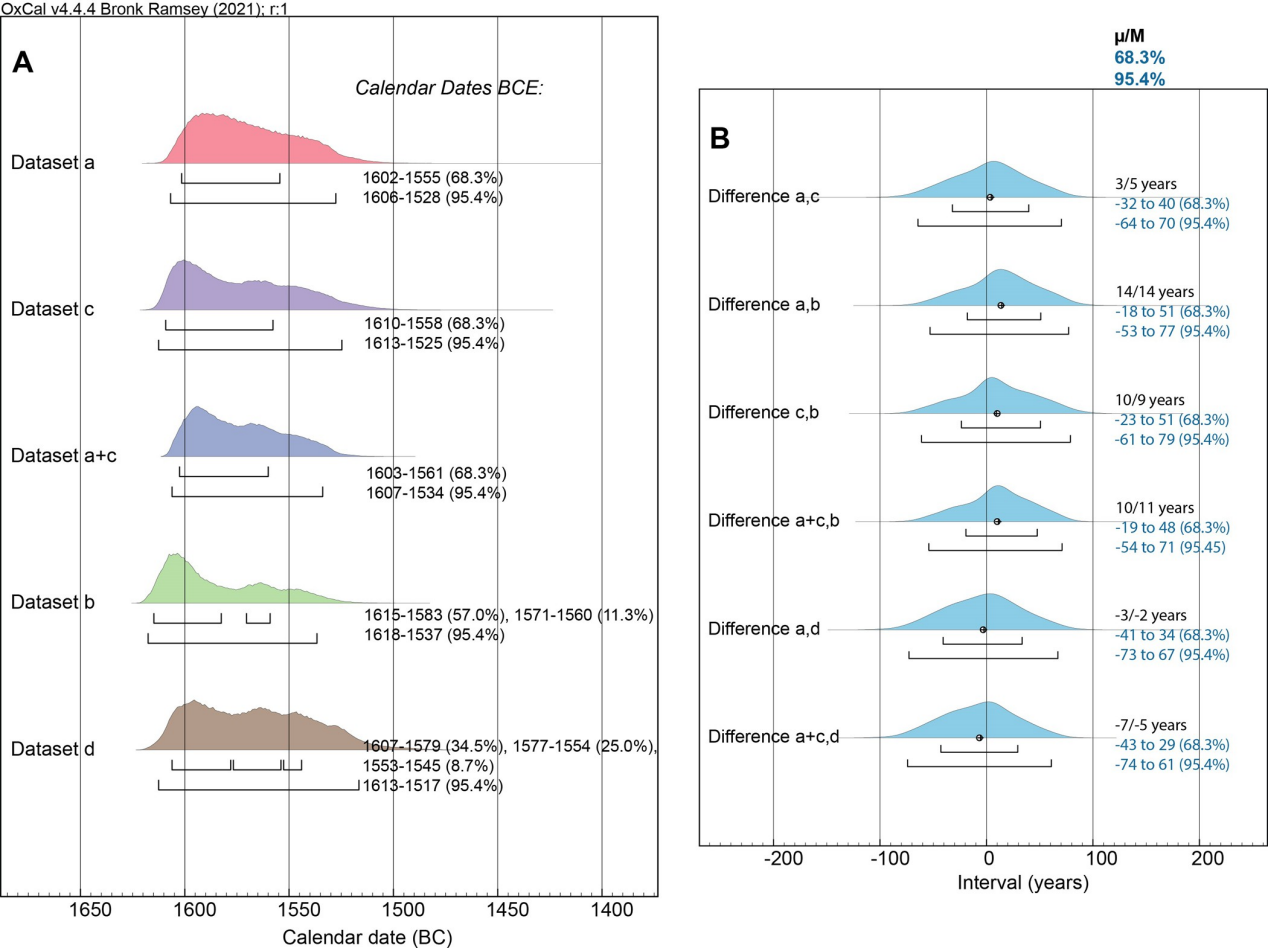
Comparison of the calendar dating probabilities for the Boundary after the datasets (a), (b), (c) and (d), and for dataset (a)+(c), and investigation of likely temporal difference between these datasets. **(A)** The posterior probability distributions from the models for each dataset and for datasets (a)+(c) combined with the 68.3% and 95.4% hpd ranges indicated by the upper and lower lines under each distribution. **(B)** The time interval (OxCal Difference query) between the posterior probability distributions–from (A)–for dataset (a) versus (c) and (a) versus (b), for dataset (c) versus (b), for datasets (a)+(c) versus (b), for dataset (a) versus (d) and for datasets (a)+(c) versus (d). The mean (μ) and median (M) of each difference is stated (in calendar years) as well as the 68.3% and 95.4% probability ranges.

**Fig 8:**
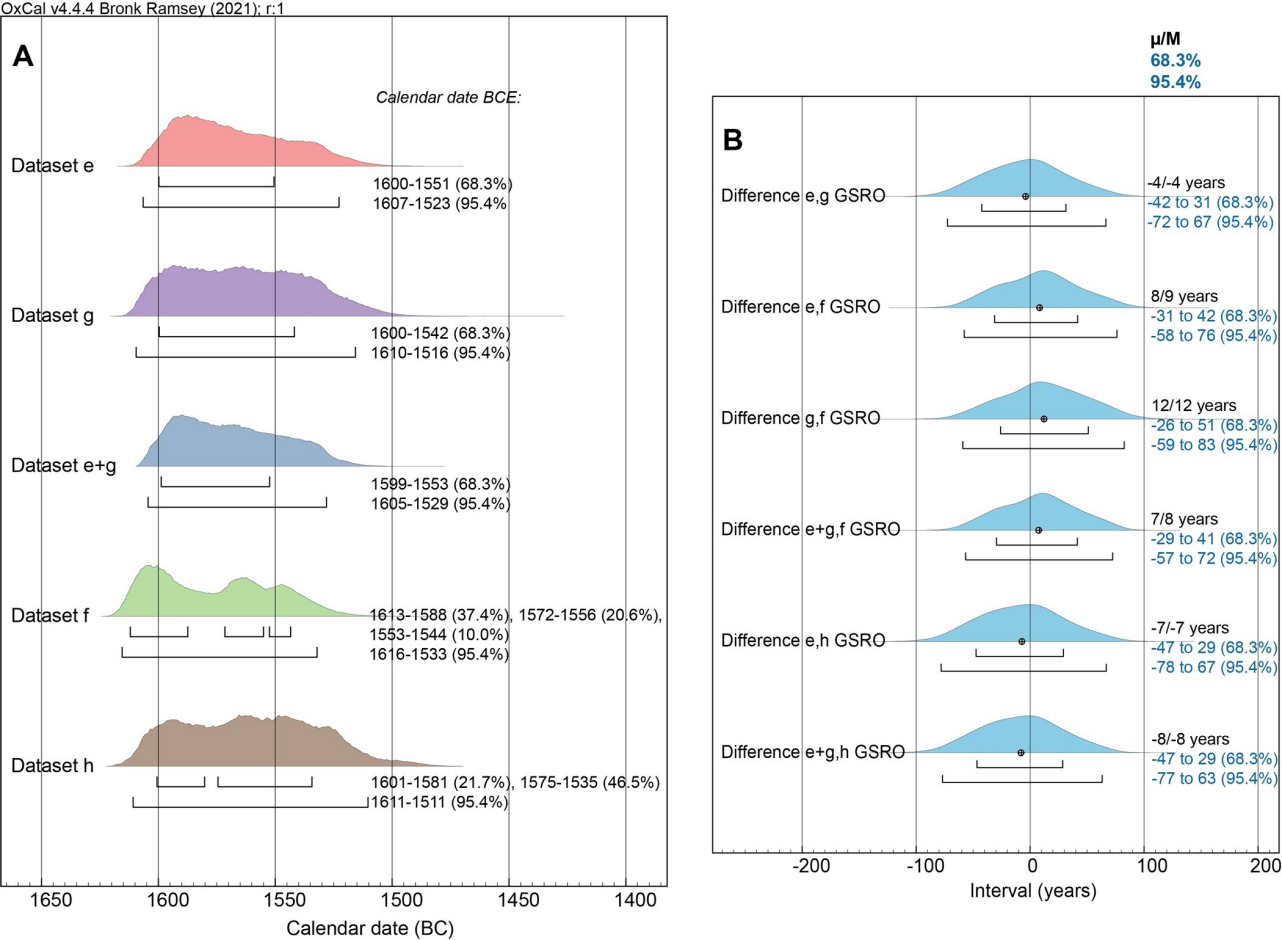
A re-run of the Fig 7 data and models, as datasets (e) to (h), but allowing in each case for the southern Aegean likely worst-case GSRO factor.

The models used for the Figs 7 and 8 results treat each of the named datasets separately (independently) (see below: Results).

**Fig 9:**
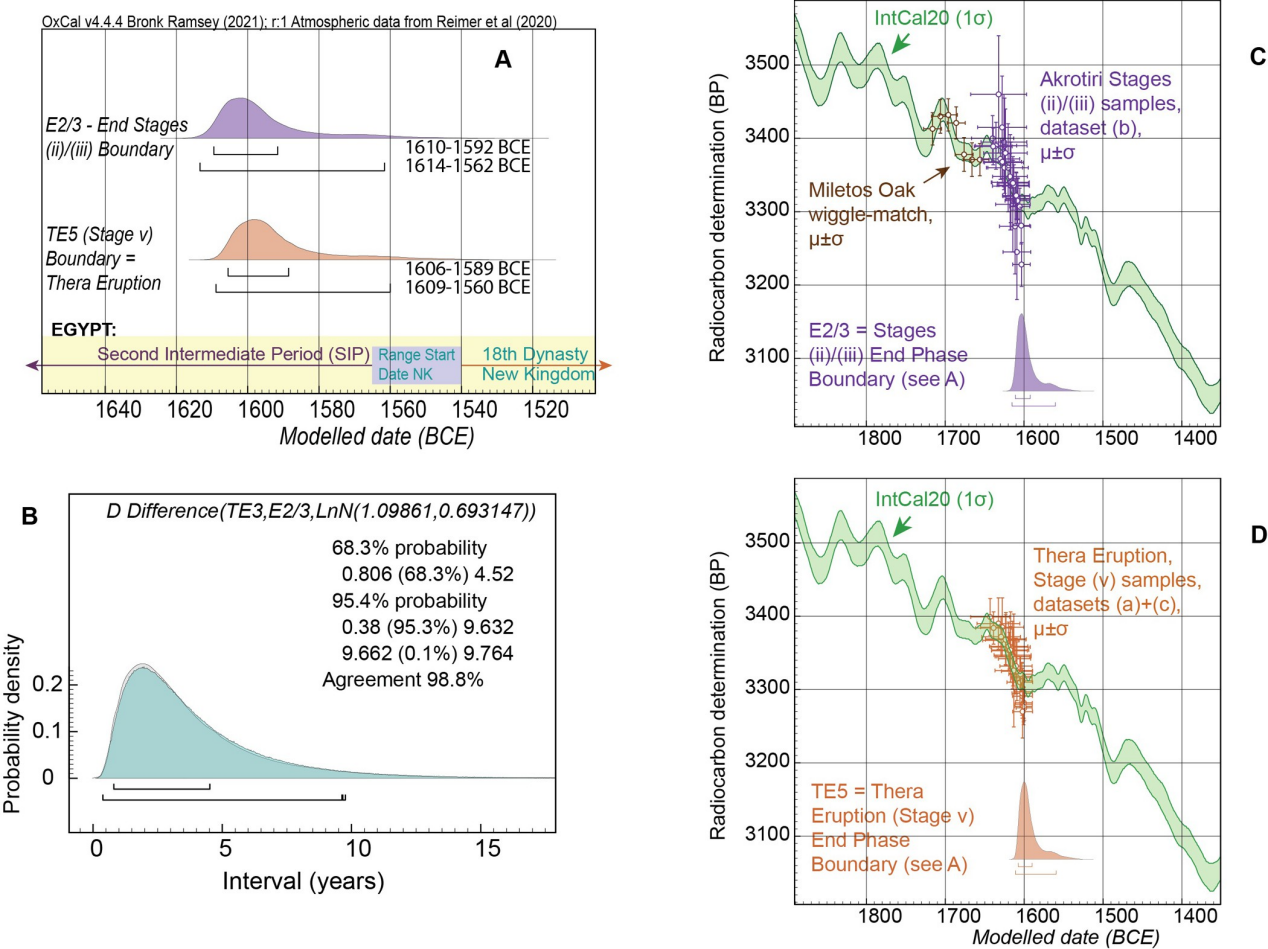
Dating of Akrotiri stages (ii)/(iii) and the Thera eruption (stage v) from Model 1. **(A)** Results for the end of Stages (ii)/(iii) Boundary and the Thera Eruption Boundary from Model 1 run 3 with log-normal, LnN(ln(3),ln(2)), constraint applied (Table 1), detailing the 68.3% and 95.4% hpd calendar age ranges. Comparison is also shown versus the approximate start date of the NK = 18^th^ Dynasty in Egypt ~1565/1540 BCE [7–9, 25, 30, 44, 61–63]. **(B)** Modelled posterior (solid, cyan) probability versus the log-normal prior (hollow distribution) for the Difference constraint. As indicated by the OxCal Agreement value of 98.8%, there is a very good correspondence between modelled result and prior assumption (see also S1 Fig). **(C)** Fit of Miletos wiggle-match and modelled dataset (b) (stages ii/iii) individual data against IntCal20 curve (1σ ages and μ±σ modelled calendar ranges) before end of Phase Boundary (see A) from Model 1. (Note: the Miletos wiggle-match oak data are shown placed against IntCal20 in Fig 9C, where they are more easily viewed, versus in Fig 9D, although they used as relevant to the non-Thera dataset (a) samples included in Fig 9D). **(D)** Fit of modelled datasets (a)+(c) (Thera eruption, stage v) individual data against IntCal20 curve (1σ ages and μ±σ modelled calendar ranges) before end of Phase Boundary (see A) from Model 1.

Consistent with (and informed by) the stratigraphic information, Model 1 used for Fig 9 (see below: Results) employs a Sequence in OxCal whereby the data from stages (ii)/(iii) at Akrotiri, Thera, are placed as before (i.e. older than) the Thera eruption (stage (v)) data with the period of time between these elements constrained, as noted above, by the log-normal constraint on a Difference query between the end Boundary for each Phase. In turn, the eruption is placed before the LHII date on animal bone from Kolonna from dataset (i)—see above. Details of results from the model and different versions (including runs without the Kolonna animal bone TAQ) are provided below in Results.

**Fig 10:**
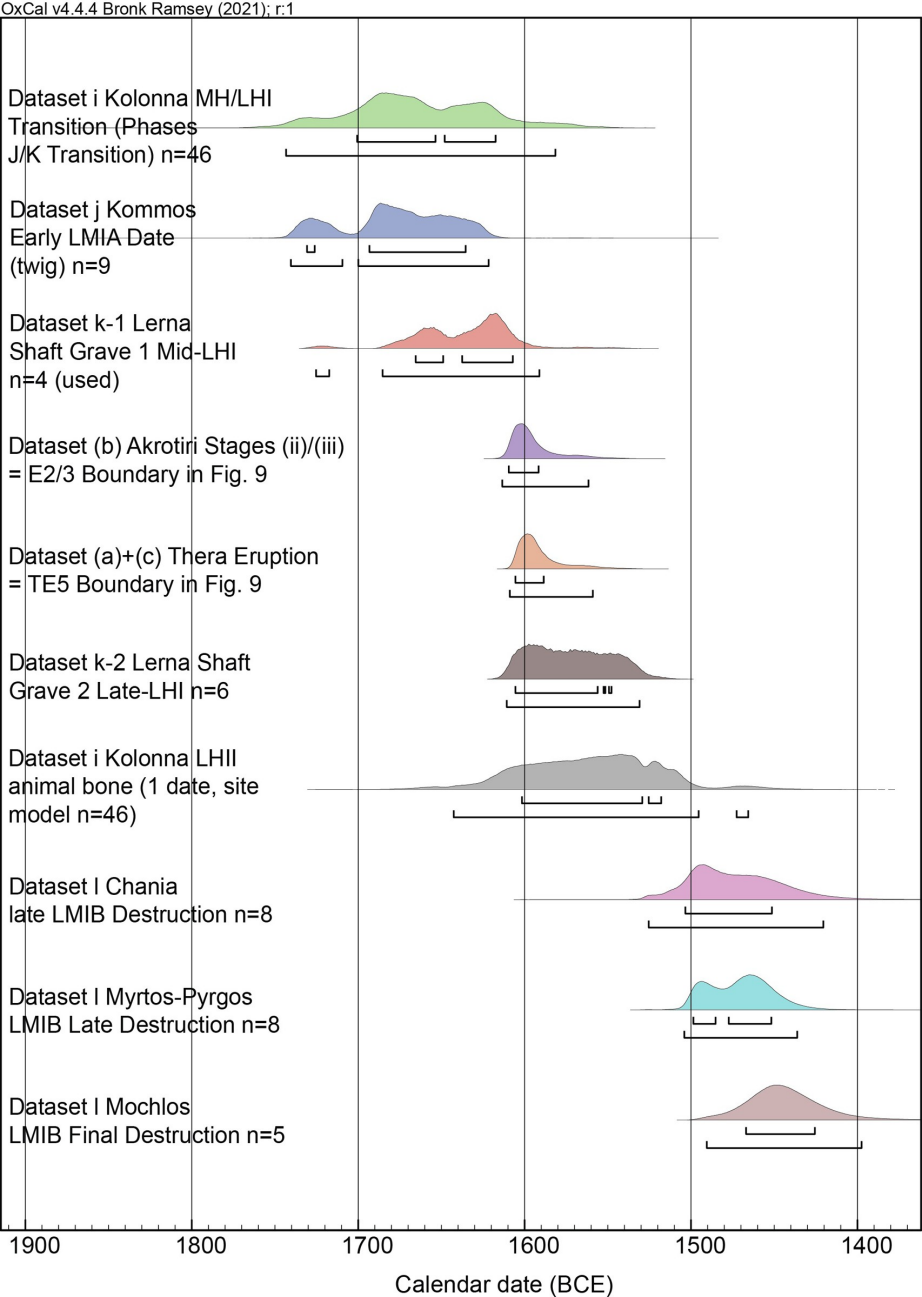
Modelled posterior probability distributions for datasets from Aegean LMIA or LCI or LHI-II contexts prior to, or around, or shortly after, the Thera eruption, and LMIB destruction datasets from the close of the subsequent archaeological period on Crete. For the 68.3% and 95.4% hpd ranges indicated, see Table 5. The datasets offer a coherent chronological sequence.

The models used for the Fig 10 results (see below: Results) comprise: (a) the modelled posterior probabilities from Model 1 (Fig 9) for the Thera stages (ii)/(iii) end Boundary and the Thera eruption Boundary; and (b) the separate (i.e. independent) runs of the dataset models for the other elements (thus dataset (i), (j), (k-1, k-2) and (l)). The modelled 68.3% and 95.4% hpd ranges are listed below in Results.

**Fig 11:**
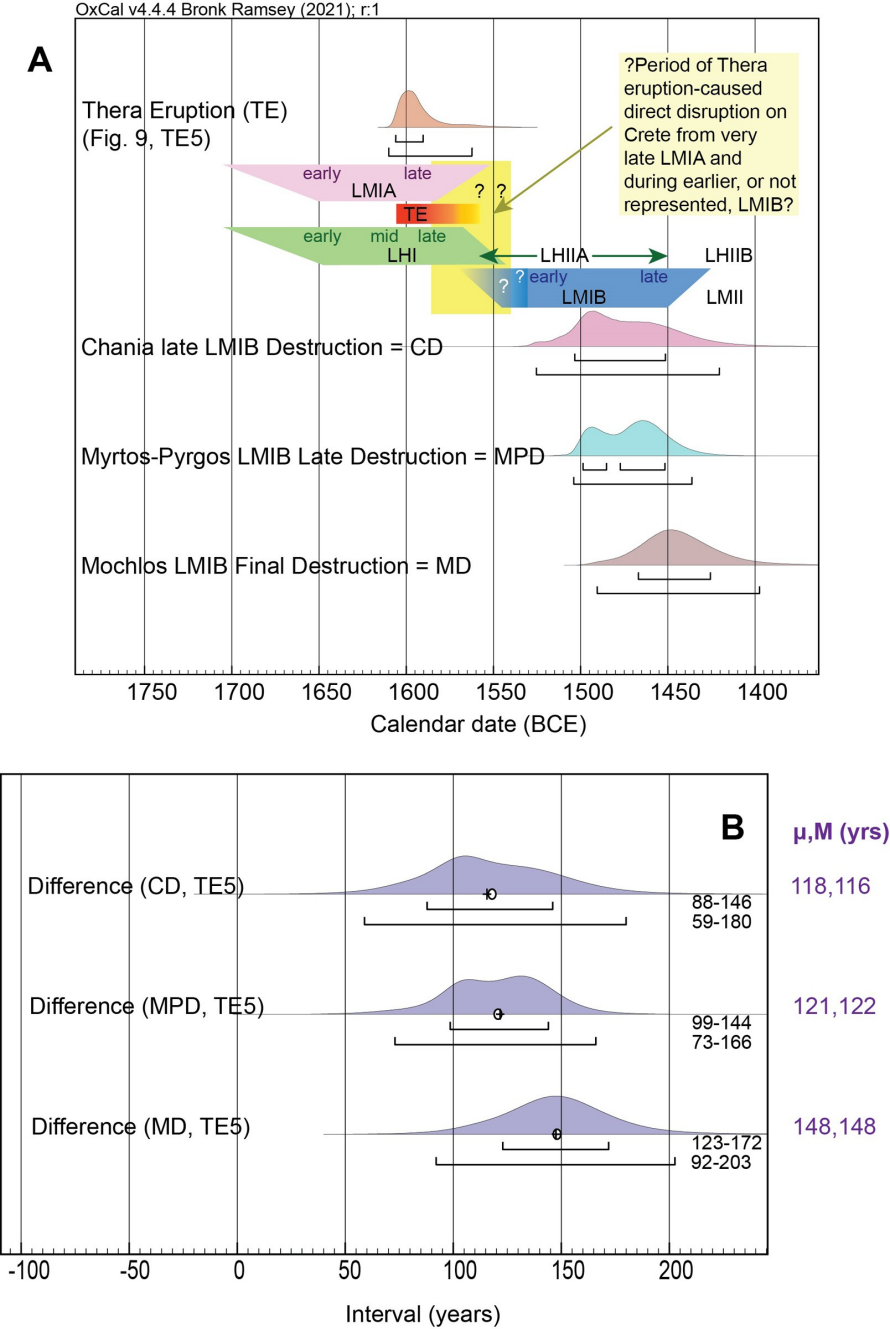
Comparison of the calendar date ranges for the close of LMIB destructions at three sites on Crete versus the Thera eruption (TE, or TE5 = stage v) and the time interval in-between. (**A**) The modelled posterior probability for the Thera eruption (Fig 9) compared with the modelled posterior probabilities for the close of LMIB destructions at Chania, Myrtos-Pyrgos and Mochlos on Crete (Fig 10). Possible archaeological period relationships are indicated along with the suggestion of a potentially important absence of evidence in the earlier-mid 16^th^ century BCE from very late LMIA through earlier LMIB, perhaps linked with the impacts and dislocation initiated by the Thera eruption. **(B)** The time interval (Difference query) between the Thera eruption and each of the LMIB destructions is shown. The mean (μ, ellipse) and median (M, cross) of each difference is stated.

The models used for the Fig 11 results (see below: Results) come from those in Figs 9 and 10.

Model 2 is different. Here one overall model is used to integrate/relate the information in Model 1 with the ^14^C information in datasets (i), (j) and (l) as well as an additional set of dates (dataset m) which may be securely related to the timeframe before, contemporary with, or after the Thera eruption to create a temporal sequence from end MBA/start LCI/LHI/LMIA through to the end of LMII. Additional short tree-ring sequenced wiggle-matches are added in this model (65/N001/I2 *Tamarix* sp. from Akrotiri, Thera; AE1024 *Quercus* sp. from Trianda, Rhodes). A set of dates on samples from an olive branch (M4N003 from Akrotiri, Thera) are also included. As noted above, visual identification of the annual growth increments of such olive samples is problematic to impossible [145]—and in this case the variations in ^14^C ages suggest the supposed ‘ring’ associations are likely entirely insecure and even an assumption of known order is dubious. Thus, since this sample ends in bark (giving the cutting/use date for the sample from a LCI period, but pre-VDL, context at Akrotiri, Thera), the data are grouped in a Phase inside a Sequence with a Tau_Boundary paired with a Boundary to best express the likely date for the cutting/use of this sample. The modelled dates for the use or relevance of the samples (like 65/N001/I2, AE1024, M4N003, or the Kommos early LMIA charred twig K85A/66B/4:22+23), or the Boundaries modelled from the available datasets (the olive branch from [80], Kolonna Transition J/K, VERA-4630, start Kolonna Phase M), are incorporated into Model 2 via cross-references. The set of dates on wood charcoal from later LMIB Trianda on Rhodes [72] offer ^14^C ages that are relatively similar with those on short-lived samples from the close of LMIB destructions from the three sites on Crete. It thus seems unlikely that there is much of an in-built age factor involved in these particular wood-charcoal samples. At the same time, none are identified to species nor characterized (e.g. short-lived twig/branch, or noted as including bark). Thus these data are modelled as a Phase describing likely some portion of mature/later LMIB, with the OxCal General Outlier model applied to each date. An OxCal Date query is used as an estimate of the calendar age represented by this set—yielding an estimate of the time between the start and end Boundaries of this Phase. The model is run with all elements including a couple of larger outliers (rather than removing these). As a result, the Model 2 A_model_/A_overall_ values are lowered and <60; however, since the data are all employed with outlier models applied, these outliers are down-weighted in the analysis, and the results reported are robust.

The OxCal runfiles for each of the models used are listed in order of use/reference in S2 Table. Each model run is very slightly different. Typically there are very small variations of 0 to a few years in quoted ranges. Comparisons of results from different runs of the same versions of Model 1 can be seen in Tables 1 and 2. At the edges of less well-defined 95.4% probability ranges the variations are sometimes a few to several years; this issue applies here especially in the mid-16^th^ century BCE at the end of the 95.4% ranges and especially for those with GRSO applied which creates slight further ambiguity. Some brief comments on the runs of the initial dataset models are given in S1 File.

**Table 1:** Model 1 (Fig 9) results. **A.** Results (**bold = 68.3% hpd**, and non-bold = 95.4% hpd) for the date of the Thera Eruption Boundary from 5 runs of Model 1 (Fig 9) with the Difference query with LnN(ln(3),ln(2)) constraint^†^, and from 5 runs of an alternative version using a Difference query with U(0,15) constraint, each without, and then with, the likely maximum southern Aegean GSRO of 4±2 ^14^C years. **B.** The same but for the Akrotiri stages (ii)/(iii) Boundary. OxCal A_model_/A_overall_ (A_m_/A_o_) values are also listed for each model. Note rounding errors sometimes see the total hpd reported vary by up to 0.1%. The results show how all runs of such models are unique and results determined can vary very slightly—especially in this case in the less well-defined margins of the 95.4% probability region on the recent side, where the calibration curve plateau lacks clear discrimination (exacerbated slightly further again when the GRSO with additional error term is applied). Run 3 (*) with equal highest A_m_ value (121.2) is illustrated in Fig 9.

Run	A_m_/A_o_	LnN(ln(3),ln(2)) Dates BCE	A_m_/A_o_	LnN(ln(3),ln(2)) with Delta_R (4,2) Dates BCE	A_m_/A_o_	U(0,15) Dates BCE	A_m_/A_o_	U(0,15) with Delta_R (4,2) Dates BCE
**A.**	**Thera Eruption (Stage v) Boundary**							
1	121/ 105	**1606–1589**	106/ 89	**1606–1583**	121/ 105	**1604–1588**	105/ 89	**1604–1583**
		1609–1560		1609–1551		1609–1559		1608–1553
2	121 /106	**1606–1589**	106/ 88	**1606–1580**	121/ 105	**1604–1587**	104/ 89	**1604–1582**
		1609–1560		1608–1548		1609–1560		1608–1550
3	121*/ 105	**1606–1589**	105/ 89	**1606–1583**	120/ 105	**1604–1587**	105/ 88	**1604–1582**
		1609–1560		1609–1551		1609–1560		1608–1549
4	121/ 105	**1606–1589**	106/ 88	**1606–1583**	120/ 105	**1605–1587**	105/ 89	**1604–1582**
		1609–1561		1609–1551		1609–1559		1608–1549
5	121/ 106	**1606–1588**	105/88	**1606–1582**	119/ 105	**1605–1587**	105/ 89	**1604–1583**
		1609–1558		1609–1550		1609–1559		1608–1551
**Av. 68.3%**		**1606–1589**		**1606–1582**		**1604–1587**		**1604–1582**
Av. 95.4%		1609–1560		1609–1550		1609–1559		1608–1550
**B.**	**Stages (ii)/(iii) Boundary**							
1	121/ 105	**1610–1592**	106/ 89	**1610–1587**	121/ 105	**1612–1594**	105/ 89	**1611–1591**
		1614–1562		1613–1555		1617–1565		1616–1560
2	121 /106	**1610–1592**	106/ 88	**1610–1588**	121/ 105	**1612–1594**	104/ 89	**1611–1589**
		1614–1563		1613–1557		1617–1567		1616–1557
3	121/ 105	**1610–1592**	105/ 89	**1609–1584**	120/ 105	**1612–1594**	105/ 88	**1612–1589**
		1614–1562		1612–1550		1617–1566		1616–1557
4	121/ 105	**1610–1592**	106/ 88	**1610–1586**	120/ 105	**1612–1594**	105/ 89	**1612–1589**
		1614–1563		1613–1555		1617–1656		1616–1557
5	121/ 106	**1610–1591**	105/88	**1610–1586**	119/ 105	**1612–1594**	105/ 89	**1611–1590**
		1614–1562		1613–1552		1617–1565		1616–1558

^†^Note: readers might ask how determinative is the LnN(ln(3),ln(2)) constraint, and wonder whether a more compressed and shorter period constraint, favoring the shorter assessments of the time interval between stages (ii)/(iii) and stage (v), variously assessed by experts as weeks/months/season(s) up to a period of several years (see Materials and methods), might make a substantive difference. To test and clarify, we may consider, e.g., the same Model 1 version shown in Fig 9 and reported in Table 1 re-run instead with a LnN(ln(0.75),ln(3)) constraint, which assumes a mode value around 2.5 months, a 68.3% hpd range from 0.04 to 1.29 years and a 95.4% range from 0.01 to 4.81 years: see S2A Fig. There is very little difference, the Thera Eruption (stage v) Boundary is 1607–1589 BCE (68.3% hpd) and 1610–1559 BCE (95.4% hpd) (to 1557 BCE in some runs), compared with the average value of 1606–1589 BCE (68.3% hpd) and 1609–1560 BCE (95.4% hpd) reported in Table 1.

**Table 2:** Model 1 re-runs without the VERA-4630 TAQ (compare with Table 1). **A.** Results (**bold = 68.3% hpd**, and non-bold = 95.4% hpd) for the date of the Thera Eruption Boundary from re-runs of Model 1 (Fig 9 and Table 1) but without the VERA-4630 TAQ. Otherwise as Table 1. The absence of the VERA-4630 TAQ leads to slightly more spread, and to minor variations at the late margins of the 95.4% ranges, in particular.

Run	A_m_/A_o_	LnN(ln(3),ln(2)) Dates BCE	A_m_/A_o_	LnN(ln(3),ln(2)) with Delta_R (4,2) Dates BCE	A_m_/A_o_	U(0,15) Dates BCE	A_m_/A_o_	U(0,15) with Delta_R (4,2) Dates BCE
**A.**	**Thera Eruption (Stage v) Boundary**							
1	120/ 104	1607–1587	104/ 87	1606–1578	120/ 105	1605–1586	105/ 89	1605–1580
		1609–1551		1608–1543		1609–1553		1608–1547 (94.4) 1545–1544 (0.3) 1541–1539 (0.7)
2	120 /104	1607–1586	105/ 86	1606–1577 (64.8) 1564–1561 (3.5)	121/ 106	1605–1586	104/ 89	1605–1576
		1609–1550		1608–1540		1609–1553		1608–1537
3	120/ 104	1607–1586	105/ 86	1606–1577	119/ 105	1605–1585	106/ 90	1605–1575 (65.3) 1568–1566 (1.7) 1564–1562 (1.2)
		1609–1552		1608–1541		1608–1553		1607–1534
4	119/ 104	1607–1586	106/ 86	1606–1578 (61.3) 1567–1561 (6.5)	119/ 106	1605–1586	105/ 91	1605–1578
		1610–1551		1608–1539		1609–1550		1608–1539
5	120/ 104	1607–1586	106/85	1606–1578 (67.1) 1561–1560 (1.2)	119/ 105	1605–1586	104/ 90	1605–1576
		1609–1551		1608–1539		1609–1550		1607–1540
**B.**	**Stages (ii)/(iii) Boundary**							
1	120/ 104	**1610–1590**	104/ 87	**1610–1581**	120/ 105	**1612–1594**	105/ 89	**1612–1587**
		1614–1554		1612–1546		1617–1559		1616–1552 (94.7) 1550–1548 (0.7)
2	120 /104	**1610–1590**	105/ 86	**1610–1581 (63.7)** **1568–1564 (4.6)**	121/ 106	**1612–1593**	104/ 89	**1613–1585**
		1614–1553		1612–1543		1617–1562		1615–1545
3	120/ 104	**1610–1590**	105/ 86	**1610–1581**	119/ 105	**1612–1593**	106/ 90	**1612–1583 (64.9)** **1568–1565 (3.4)**
		1614–1555		1612–1544		1617–1560		1615–1542
4	119/ 104	**1610–1590**	106/ 86	**1609–1582 (61.3)** **1570–1564 (7.0)**	119/ 106	**1612–1593**	105/ 91	**1612–1586**
		1614–1555		1612–1543		1617–1559		1616–1547
5	120/ 104	**1610–1590**	106/85	**1610–1582 (65.5)** **1577–1561 (2.7)**	119/ 105	**1612–1593**	104/ 90	**1612–1583**
		1614–1554		1612–1543		1617–1557		1615–1546

### Inclusivity in global research

Additional information regarding the ethical, cultural, and scientific considerations specific to inclusivity in global research is included in the (S2 File, Checklist).

## Results

### Resolving the question of a volcanic CO_2_ effect on the Thera VDL samples

The initial requirement to make progress with a date for the Thera eruption is to identify whether or not a volcanic CO_2_ effect applies to the dates from the Thera VDL, and so whether this issue may (or may not) be discounted in this particular case. If it can be eliminated, then substantial further progress is possible. This applies both to dating the eruption itself, but also to establishing the time relationships between the eruption and other ^14^C-dated contexts, including the close of LMIB destructions on Crete.

The Boundary after the dataset (a) Phase should estimate the date of the Thera eruption’s date from its distant (>200km) effects via eruption-linked tsunami and direct airfall tephra impacts. No volcanic CO_2_ effect is plausible. The Boundary after the dataset (c) Phase should estimate the time olive trees on Thera were likely killed by the Thera eruption. In calendar terms (a) should approximately equal (c). If there is no substantive difference between these two date estimates, then there is no evidence for a substantive volcanic CO_2_ effect applying on Thera affecting the olive samples; or, alternatively, the reverse applies if there is a substantive difference. The Boundary after dataset (b) should yield the date of the Phase (ii)/(iii) abandonment of Akrotiri. This should be very slightly older than the date of the Thera eruption, by a period of weeks/months/season(s) up to a period of several years (see above, Materials and methods). Thus, if dataset (b) is only a little older than dataset (a) then this is consistent with known expectations as long as this difference is plausibly within a period of no more than several years older. If so, then there is no evidence for a substantive volcanic CO_2_ effect affecting the archaeological samples from the Akrotiri VDL. Alternatively, if there is a major temporal difference, then the reverse applies. Similarly, the Boundary after dataset (c) should be a little (but no more than several years) later than the boundary after dataset (b), and similar in scale to the dataset (a) versus dataset (b) case. Such a small difference would indicate no substantive volcanic CO_2_ effect applies, and, alternatively, a large difference would indicate the reverse. If there is no substantive difference between datasets (a) and (c), then we might consider a combination of dataset (a)+(c) as a best estimate for the approximate date of the Thera eruption and for comparison with the other datasets (e.g. dataset (b)). Finally, we can compare the Boundary after the dataset (a) Phase with the Boundary after the dataset (d) Phase. Dataset (d) contains all the data from VDL or pumice-covered contexts on Thera, so stages (i)/(ii) and (ii)/(iii) and (v)) including dates from older technology measurements—but excluding the dates on olive wood samples in dataset (c). Most of dataset (d) should date a little older than dataset (a); hence we might expect a result similar to those analyses comparing dataset (a) with dataset (b). Such a finding and only small differences would again suggest no substantive volcanic CO_2_ effect applies for these data; alternatively, a larger difference would indicate the reverse.

Fig 7 shows the comparisons of the Boundaries after the Phases with datasets (a), (b) (c) and (d), and dataset (a)+(c), comparing the 68.3% and 95.4% hpd ranges and also the Difference between the dataset (a) probability distribution versus datasets (c), (b) and (d) probability distributions and the same for the dataset (c) probability distribution versus the dataset (b) probability distribution, and for dataset (a)+(c) versus dataset (d). Fig 8 shows a repeat considering the effect of a worst-case GSRO of 4±2 ^14^C years for datasets (a), (b), (c), (d) and datasets (a)+(c) (as datasets e–h and dataset (e)+(g)).

Comparison of dataset (a) versus dataset (c) shows only a very small/negligible difference (mean, μ, median, M: 3/5 calendar years; -4/-4 years with GSRO in Fig 8). This contradicts both any substantive volcanic CO_2_ effect applying to the available samples relevant to the Minoan eruption of Thera, and suggestions of a substantial missing period of time between the last dated olive-wood segments found on Thera and the time of the eruption [147]. In particular, if recent work on ^14^C dating olive stem sections [147, 148] is examined, it is evident that approximately correct (known age) results are found for outermost wood along the visible growth nodes (e.g. see [148] at Fig 2), with older ages reported for non-growth-node wood from elsewhere around the circumference (where many years of growth may be compressed into, or even missing from, this portion of the circumference). The wood segments ^14^C-dated across the olive branch from Thera in [80] come from a growth node (see [80] at Fig 1), and thus not only progress from older to more recent in a temporal sequence [80, 81, 146], but it is likely that the outermost segment reflects the active and most recent growth period of the tree and hence approximately the time when the tree was killed and buried by the Thera volcanic eruption. In the case of the ^14^C dates from other olive wood samples from Thera (dataset c), this observation confirms that it is appropriate to use an assumed exponential distribution of the dates with the most recent ages likely from outermost segments from growth nodes and hence it is these dates that reflect last growth and hence a close to immediate TPQ for the eruption.

Therefore, overall, we may regard the available dates from the Thera VDL as representative and not affected by any substantive volcanic CO_2_ effect, and so go ahead to use these data as part of efforts to achieve a likely representative date estimate for the timing of the Thera eruption.

### Modeling the temporal sequence immediately leading to the Thera eruption and resolving the Thera eruption date range

In line with the archaeological-geological observations, the comparison of dataset (b) versus dataset (a) shows that dataset (b) exhibits slightly older ages than dataset (a) by (μ, M) 14/14 calendar years (8/9 years with GSRO). This is consistent with the archaeological sequence observed (see above), where stages (ii)/(iii) are earlier than stage (v) by as much as “a period of several years” [105]—noting that the calibration curve taphonomy (reversal-plateau) (Fig 2) tends to exaggerate any difference in calendar terms because there is spreading of the (lower) probability tail widely across the 16^th^ century BCE (Figs 7 and 8). The comparison of dataset (c) versus dataset (b) provides a similar picture: dataset (b) is slightly older by (μ, M) 10/9 calendar years (12/12 years with GSRO). The combination of ^14^C dates from different technologies (conventional and AMS) and run some time ago and more recently, for VDL samples from Akrotiri and from elsewhere on Thera (dataset d), on a ‘wisdom of crowds’ approach despite some noise in the set especially amongst the non-AMS results (Fig 4), again yields similar age relationships indicating no large discrepancy (and so no substantive volcanic CO_2_ bias). There is only an overall small offset versus datasets (a) or (a)+(c) (Figs 7 and 8), with μ/M -3/-2 or -7/-5 calendar years (-7/-7 or -8/-8 years with GSRO). It is thus evident that different samples from different sites run by different radiocarbon laboratories (and slightly varying methods) have in general all produced sets of relatively similar results, suggesting we have representative and robust datasets and information. We focus our dating efforts on, and draw conclusions from, the samples run more recently, from good contexts, and with proper (modern) pretreatment—thus datasets (a), (c) and (b) for the Thera eruption.

A Sequence model (Model 1) integrating the ^14^C dates from datasets (a), (b) and (c) with the observed stratigraphic sequence and using the proposed modeling approach (see Materials and methods above) is shown in Fig 9 (for results from this model see Tables 1 and 2, for an alternative version, see S1 Fig). The eruption date range is given by the end Boundary immediately after the Phase with the combined datasets (a)+(c). Quoting the averages from 5 model runs (Table 1), this is most likely 1606–1589 BCE (68.3% hpd) and 1609–1560 BCE (95.4% hpd); or 1606–1582 BCE (68.3% hpd) and 1609–1550 BCE (95.4% hpd) allowing for the possible maximum GSRO. The posterior probability for the time interval between stages (ii)/(iii) to (v) corresponds well with the prior assumption (Fig 9B), suggesting that it is appropriate. The data (the ^14^C ranges and properties in each Phase set) moreover offer clear and specific visual best fits against IntCal20 (Fig 9C and 9D) in the periods immediately before each end of Phase Boundary (Fig 9A), with the constituent data in each case lying on the steep slope in the ^14^C calibration curve between ~1640–1600 BCE. Given these observations, there is not surprisingly very good agreement of data, model, and calibration curve. Only one date, DEM-1607, has an outlier probability >7% (at 9% or 10% across runs), and just 3 other dates have outlier probabilities of 6% (VERA-5610) (or sometimes 6%: OxA-12305) or 7% (OxA-12303). Potentially as, or more, important is the modelled age probability for dataset (b), stages (ii)/(iii), in Fig 9: 1610–1592 BCE (68.3% hpd), 1614–1562 BCE (95.4% hpd) (for other runs and models, especially Model 1 without the VERA-4630 TAQ: see S1 Fig and Tables 1 and 2). This provides a TAQ for the extraordinary LCI material culture and wall-paintings recovered at Akrotiri from before the extinguishing VDL episode [46, 50, 51, 149].

In support of the results in Fig 9, the sets of ^14^C dates from other LMIA–IB or LHI Aegean contexts yield very similar and compatible findings (Fig 10). The further question is the relationship of the Thera eruption date with regard to the timing of the close of LMIB destructions on Crete [3, 17, 49, 58–60]. Fig 11 compares the dates of the close of period destruction episodes for three LMIB sites on Crete (dataset l) versus the Thera eruption (Fig 9), and shows the length of time in calendar years between the eruption and each of these LMIB destructions. The mean/median differences range from 116–148 calendar years (59–203 calendar years at 95.4% hpd).

**Fig 12:**
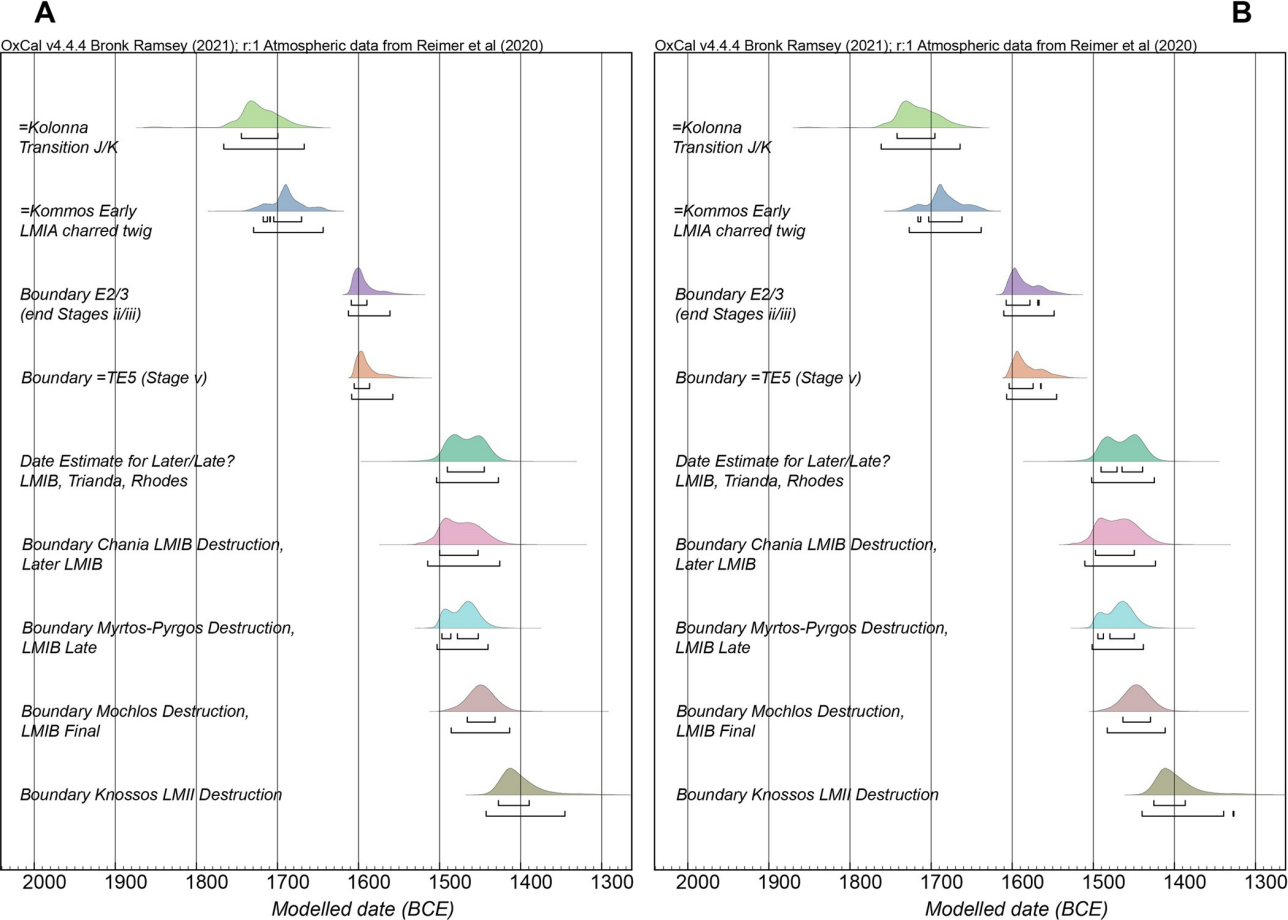
Dating results for the main elements of Model 2 integrating datasets (i), (j), (l) and (m) with Model 1 (as used for Fig 9) (datasets (a)-(c)) to give a temporal sequence running from end MBA/start LCI/LHI/LMIA through to the end of LMII. **(A)** Model 2 with no GSRO. **(B)** Model 2 with the southern Aegean maximum GSRO test of 4±2 ^14^C years. For the 68.3% and 95.4% hpd calendar age ranges indicated (upper and lower lines under each probability distribution, respectively), see Table 3. For results from re-runs of Model 2 without VERA-4630, see Table 4.

Model 2 offers an integrated analysis combining Model 1 with the other datasets securely placed before and after the Thera eruption. The results for the main elements of Model 2 are shown in Fig 12 (note: some labels are edited to fit the available display space) and the 68.3% and 95.4% hpd ranges are listed in Table 3. The results for the dating of the Akrotiri stages (ii)/(iii) Boundary and the Thera eruption Boundary are very similar to those calculated in Fig 9 (Tables 1 and 2). Results from a version of Model 2 allowing for the likely maximum possible GSRO are also shown. As explained above, the dataset (k) dates from the Lerna Shaft Graves are not used in the Model 2. The results of re-runs of the Model 2 without the inclusion of the VERA-4630 TAQ are listed in Table 4 for comparison with those in Table 3.

**Table 3:** Results (bold = 68.3% hpd, and non-bold = 95.4% hpd) for selected elements from Model 2 (Fig 12), typical examples. As in Model 1 (Fig 9), a LnN(ln(3),ln(2)) constraint is applied to a Difference query for the period of time between the Thera eruption (TE5 Boundary) and stages (ii)/(iii) at Akrotiri (E2/3 Boundary). Results are shown for the model run without, and then with, the likely maximum possible GSRO for the southern Aegean (see above). The clear majority range, if present, where there are two or more split ranges is underlined. Note rounding errors sometimes see total hpd reported vary by up to 0.1%.

Model Element	No GSRO Model Dates BCE	GSRO model Dates BCE
Kolonna Transition J/K = Middle Helladic to LHI transition, so TPQ for start LHI	**1745–1701**	**1743–1696**
	1767–1667	1762–1665
Kommos Early LMIA charred twig	**1720–1712 (6.9)** **1711–1709 (2.1)** **1703–1671 (59.4)**	**1717–1713 (2.4)** **1704–1663 (65.9)**
	1732–1645	1728–1639
E2/3 Boundary (End Stages ii/iii)	**1609–1588**	**1608–1579 (66.4)** **1569–1567 (1.9)**
	1612–1558	1611–1549
TE5 Boundary (Stage v, Thera Eruption)	**1606–1584**	**1604–1575 (67.4)** **1566–1565 (0.8)**
	1608–1556	1607–1546
*Interval not represented*, *Post-Eruption Final LMIA to Earlier LMIB*	***32–101 years***	***27–97 years***
	*0–115 years*	*0–114 years*
Date Estimate for Later/Late? LMIB, Trianda, Rhodes	**1491–1445**	**1491–1471 (29.2)** **1465–1440 (39.1)**
	1505–1427	1503–1425
Chania LMIB Destruction, later LMIB Boundary	**1500–1453**	**1498–1450**
	1515–1426	1511–1424
Myrtos-Pyrgos Destruction, LMIB Late Boundary	**1498–1487 (17.6)** **1479–1453 (50.7)**	**1495–1488 (9.4)** **1480–1450 (58.9)**
	1503–1441	1502–1439
Mochlos Destruction, LMIB Final Boundary	**1466–1432**	**1464–1430**
	1487–1414	1483–1412
Knossos Destruction LMII Boundary	**1428–1388**	**1426–1387**
	1442–1340	1440–1340 (95.3) 1328–1327 (0.1)

**Table 4:** Results (bold = 68.3% hpd, and non-bold = 95.4% hpd) for selected elements from re-runs of Model 2 without the VERA-4630 TAQ, typical examples. Compare with Table 3.

Model Element	No GSRO Model Dates BCE	GSRO model Dates BCE
Kolonna Transition J/K = Middle Helladic to LHI transition, so TPQ for start LHI	**1745–1700**	**1742–1692**
	1766–1669	1760–1662
Kommos Early LMIA charred twig	**1719–1711 (6.7)** **1704–1672 (61.6)**	**1699–1661 (61.5)** **1659–1658 (0.9)** **1657–1650 (5.9)**
	1731–1645	1725–1637
E2/3 Boundary (End Stages ii/iii)	**1610–1586**	**1607–1580 (57.0)** **1571–1562 (11.3)**
	1613–1553	1610–1542
TE5 Boundary (Stage v, Thera Eruption)	**1606–1583**	**1603–1575 (58.7)** **1568–1561 (9.5)**
	1609–1549	1606–1539
*Interval not represented*, *Post-Eruption Final LMIA to Earlier LMIB*	***30–100 years***	***23–92 years***
	*0–115 years*	*0–112 years*
Date Estimate for Later/Late? LMIB, Trianda, Rhodes	**1491–1445**	**1491–1471 (28.5)** **1466–1440 (39.7)**
	1504–1428	1502–1425
Chania LMIB Destruction, Later LMIB Boundary	**1500–1453**	**1498–1450**
	1515–1426	1511–1424
Myrtos-Pyrgos Destruction, LMIB Late Boundary	**1498–1486 (18.0)** **1478–1453 (50.3)**	**1494–1489 (7.7)** **1480–1450 (60.5)**
	1503–1441	1502–1438
Mochlos Destruction, LMIB Final Boundary	**1466–1432**	**1464–1430**
	1487–1414	1483–1411
Knossos Destruction LMII Boundary	**1428–1390**	**1426–1387**
	1443–1345	1441–1338

## Discussion

### Thera eruption date range

The analysis and comparison of the ^14^C date sets associated with the Thera eruption, from contexts on Thera itself, and from well away from Thera (>200km distant), shown in Fig 7, collectively demonstrate an absence of any general volcanic CO_2_ aging effect as relevant to the available Thera VDL datasets. Therefore, we may regard the present set of ^14^C dates from defined VDL contexts on Thera as accurately defining their sample ages. In view of what is known of traditional and ancient agricultural practices and storage at Akrotiri [125, 141, 142], the short-lived sample material likely offers dates within ~0–2 years of the find context and other short to shorter-lived sample material (animal bones from likely use as food, twigs, branches and outermost tree-rings) will not include more than a few years of in-built age. The OxCal modelling of the destruction and specific use event Phases as exponentially distributed towards the end of the Phase, with the destruction/event as the Boundary immediately following, thus likely accurately describes the data, including, but not over-estimating the age because of a few samples that are older, residual, or include more substantial in-built age (e.g. wood charcoal that is neither outer rings nor relatively short-lived material) [7, 11, 57, 133]. In turn, analysis using these ^14^C datasets should provide accurate and robust dating. At the same time, the comparison of datasets (a) and (c) versus (b) indicates a temporal sequence consistent with archaeological observations where secure stages (ii)/(iii) organic material from Akrotiri reflect abandonment of the site a little earlier than the eruption (stage (v) at Akrotiri). This further supports use of the stratigraphically-informed modelling approach allowing for a short interval between stages (ii)/(iii) and (v). The findings reported in Fig 8 considering the possible effect of a likely maximum possible GSRO, suggest that, if relevant in this case, it is very minor (see also Tables 1–4).

Bayesian analysis integrating the stratigraphically defined sequence information and expert assessment with the large set of available ^14^C dates relevant to the dating of the Akrotiri stages (ii)/(iii) abandonment and the Thera eruption (Akrotiri stage (v)) in Model 1 provides clear visual best fits for both sets of data against the northern hemisphere atmospheric ^14^C record (IntCal20): Fig 9C and 9D. The modelled probability in Fig 9A is strongly skewed towards the older age direction (to the period where the shape of the calibration curve offers a good fit for the range/properties of the ^14^C date sets for the Phases comprising datasets (a)+(c) and (b)). The modelled Thera eruption age range from Model 1 (Fig 9) and/or the other variations and models in Figs 10–12 and S1 and S2 and Tables 1–4 suggest, even at the limits of 95.4% probability, that possible volcanic signals or paleoclimatic or environmental events in various archives (ice-cores, tree-rings, speleothems) before ~1610 BCE or after ~1560/50 BCE are now very unlikely to represent the Minoan Thera eruption. (And it remains possible, especially since the Minoan eruption of Thera was not particularly sulfur-rich, that in fact Thera is not clearly represented in some or more of the volcanic eruption proxies provided by ice-core and tree-ring archives: see next section below.) In combination with other recent data and reassessments [14, 70, 150], this finding removes many past positions or suggestions for the date of the Thera eruption (e.g. several past suggestions for a date in the mid to later 17^th^ century BCE, 1640s-1620s BCE) as now unlikely or not possible, and revises and refines even recent observations and suggested possible proxy associations.

The most likely 68.3% hpd range in Fig 9, 1606–1589 BCE, does not in fact correspond with most of the possible proxy volcanic eruption signals noted so far in ice-core, tree-ring and other records—only the bristlecone pine minima 1597 BCE overlaps—with the other signals noted in this vicinity just outside this range: 1611/1610 BCE, 1586 BCE and 1584 BCE [12–14, 44, 70]. However, clearly, all these signals are near enough (or within the 95.4% range) to deserve further investigation in an attempt to identify a possible specific Thera eruption signature. The ice-core volcanic eruption signals noted for 1561, 1558, 1555 and 1550 BCE [14] are either barely in, or just outside, the (poorly defined) late end tail of the most likely 95.4% range. In terms of the Boundary TE5 (Stage v) = Thera Eruption shown in Fig 9A, <1% of the probability within the most likely 95.4% range occurs after 1562 BCE. Thus, although these possible volcanic signals should not be excluded for investigation, given there are small dating flexibilities on both sides (but see further below if we consider adding the Sofular Cave speleothem into the date modelling), nonetheless, these later volcanic signals appear now much less likely to have relevance to the Thera eruption case than the plausible candidates within or close to the most likely 68.3% dating range(s) identified above for the Thera eruption period (Figs 9–12 and Tables 1–4).

### Thera eruption proxy identification and adding the Sofular Cave evidence?

When attempting to identify the Thera eruption in various proxies recording or potentially recording larger volcanic eruptions, investigations have highlighted an important revision to past assumptions. While the Minoan eruption of Thera was huge in terms of rock-equivalent volume produced [1], and despite some uncertainties and potentially complicating factors (e.g. [151, 152]), the composition and nature of the Thera magma suggest that the Minoan eruption of Thera was not a particularly sulfur-rich eruption [153–155], and so, despite its enormous scale, the Thera eruption may not have had a very large sulfur deposition onto Arctic and Antarctic icesheets, nor had a particularly large aerosol-related climate impact globally [13, 14, 153, 154]. Thus, in a revision to previous logic (tending to link the Thera eruption with the biggest or only known signal in the period, e.g. [156–158]), the Thera eruption might well not be represented by one of the largest, or even larger, sulfur signals in polar ice sheets, and might instead be represented by one of the more modest volcanic, or potentially volcanic, signals in the ice or tree-ring records like one of those ~1611/1610 BCE, 1597 BCE, 1586 BCE or 1584 BCE [13, 14], or, indeed, despite its cataclysmic relevance to the southern Aegean and surrounding region, the Minoan Thera eruption might not even offer a clear signal in one or more of these types of distant proxy records [159, 160].

At this point one local Anatolian proxy might be brought into the discussion. The date range reported above for the Thera eruption is consistent with the reported likely volcanic eruption signal(s) recorded in the Sofular Cave speleothem in northwest Turkey [161], with a bromine (Br) peak 1621±25 BCE, then a molybdenum (Mo) peak 1617±25 BCE, and finally a sulfur (S) peak centered 1589±25 BCE. The first two dates are very similar and there is a plausible set of mechanisms explaining the slight timing offsets in the speleothem record ([161] at pp.62-63). The slightly later and less sharply-defined sulfur peak is as expected, given understanding of soil-vegetation processes and hence transfer times for sulfur between availability from the eruption to speleothem inclusion, that will likely involve a period up to 15–30 years ([161] at p.63). Hence it is the first two dates from the bromine and molybdenum that are relevant. While the dating available from the Sofular Cave speleothem record is not high-precision, these two dates both include the ^14^C range defined above, and have the merit of deriving from an independent timescale, and they have a plausible geographic-geochemical association with the Thera eruption [161]. Thus the question is whether these signals are likely to represent the Thera eruption (e.g. [13] at pp.174-175 expresses some caution/caveats)?

There is clearly no definite positive case at present. However, several observations are possible which support a Thera eruption association. The published Sofular Cave Br record (starting just after 1640 BCE and running to 1415 BCE—noting the Sofular chronology is ±25 years) has just one conspicuous peak 1621 BCE. There is a much smaller peak in the mid-1630s BCE and an even smaller one just before 1600 BCE and lesser little peaks in the mid-1590s and earlier 1580s BCE. The spacings do not suggest any clear or systematic associations with the major volcanic eruptions of the period as listed in [14] at Table 2 (even moving the dates systematically up or down within the ±25 years dating error). Instead, the single clear peak 1621 BCE suggests a specific, and thus likely proximate, cause for the large 1621 BCE signal. The Mo record, also covering the period just after 1640 to 1415 BCE, has the one major peak around 1617 BCE, with the only other notable peaks just after 1570 BCE and then a smaller peak about 1565 BCE. The spacings do not suggest any likely pattern of associations with the major eruptions of the period as listed in [14] at Table 2, and there is just the one major peak within the available record, suggesting again a specific, local, source. Finally, the S record (starting 1665 BCE) shows just the one major peak (centered 1589 BCE) between 1665–1415 BCE. There are some other modest increases in the late 1620s, early 1560s and mid-1540s BCE, but all are much smaller, and thus, if the S record is associated with volcanic eruptions, the issue is why the large and sustained peak around 1589 BCE is so very much more conspicuous? Again, the only plausible explanation appears to be a large, local, volcanic source. For these reasons it would seem plausible that the signals in the Sofular Cave speleothem not only reflect a volcanic eruption [161], but that it is likely to be a large, local, eruption and this forms the distinctive feature that explains the available record. If so, then this is likely the approximately contemporary Minoan eruption of the Thera volcano.

**Fig 13:**
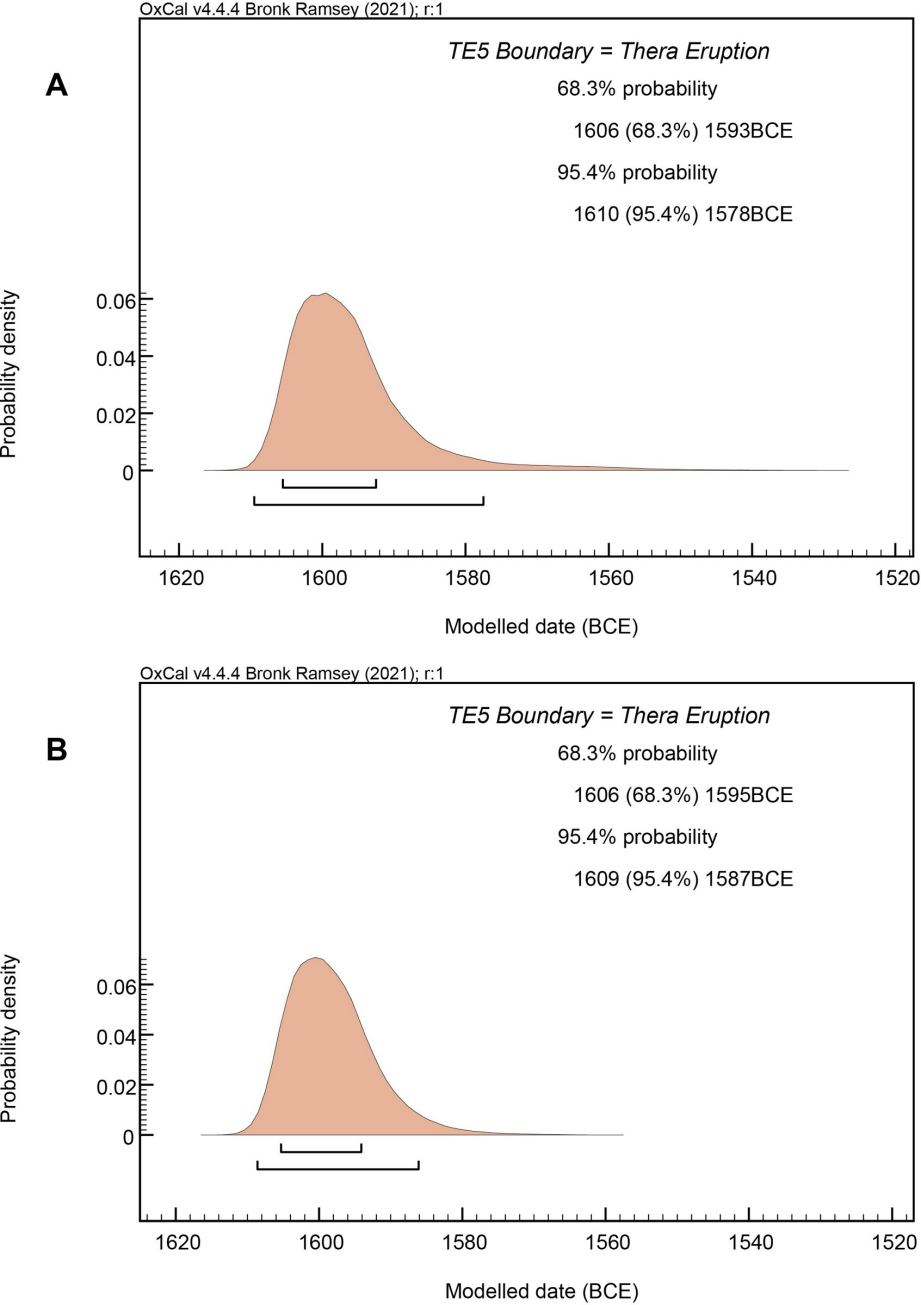
Dating of Akrotiri stage (v) = Thera eruption Boundary from Model 1 in Fig 9 re-run incorporating the Sofular Cave dates for likely Thera-eruption associated spikes in bromine (Br) and molybdenum (Mo) [161]. **(A)** with the two Sofular Cave dates treated independently, **(B)** with the two Sofular Cave dates combined.

Therefore, while only a plausible scenario versus definite, we might view the Sofular Cave evidence and its approximate timescale as likely both adding to the information for, and being constrained by, the ^14^C defined time range for the Thera eruption. The likely near-contemporary evidence from the Br and Mo peaks are the relevant evidence, versus the expected much-delayed sulfur rise. We may include the Sofular Cave approximate dates into the Thera eruption dating models used above. Fig 13 shows the modelled Thera eruption Boundary from Model 1 (in Fig 9) revised to include a Date query for a Phase comprising the bromine peak 1621±25 BCE and the molybdenum peak 1617±25 BCE added into the Boundary representing the date estimate for the Thera eruption. The addition of the independent Sofular Cave date estimates reduces the Thera dating probability in the mid-16^th^ century BCE and instead acts to concentrate likely dating probability around ~1600 BCE. Fig 13A shows the Thera eruption Boundary including the bromine and molybdenum peak dates treated separately in a Phase with a Date query, while Fig 13B uses a Date query for the Phase when it contains a combination (OxCal Combine command) of the (very similar) bromine and molybdenum peak dates (as representing a common date estimate) (see S2 Table). In both cases the inclusion of the Sofular Cave dates reduce the 95.4% hpd Thera eruption ranges, respectively to 1610–1578 BCE and 1609–1587 BCE, with the most likely 68.3% ranges respectively 1606–1593 BCE and 1606–1595 BCE. These date ranges including the Sofular Cave evidence might further focus attention, if relevant, towards the ice-core and/or tree-ring proxy volcanic-environmental signals ~1611/10, 1597, 1586 or 1584 BCE [13, 14]. Most importantly, if it is a valid assumption to integrate the Sofular Cave evidence, then this would help more firmly to exclude as plausible some dates for possible volcanic proxy evidence that have been suggested as potentially relevant for the Thera eruption around 1561/1560, 1558, 1555/1554, 1546, 1544, 1539/1538 or 1524 BCE [13, 14].

### Implications of a Thera eruption date ~1609–1560 BCE and likely ~1606–1589 BCE (Fig 9)

The importance of accurate chronology in archaeology is that it provides the correct (that is real) timetable and synchronization of past events, episodes, peoples, places and interactions, and therefore the ability to address an historical analysis and narrative. This is why the topic of the date of the Thera eruption has been controversial and why suggestions to revise the date have been strongly resisted. There was a generally agreed approximate chronology and cultural synchronization, and thus history, of the East Mediterranean in the mid-second millennium BCE. This placed the beginning of the Aegean Late Bronze Age contemporary with the start of the New Kingdom (18^th^ Dynasty) in Egypt [27, 84]. But, if the date of the Thera eruption is shifted by a substantial amount, then this implies sets of different relations/connections for the Aegean and East Mediterranean with Egypt and the Near East: a new history, new questions.

Is it time for a new history for the Thera eruption period based on directly relevant ^14^C evidence? Up until now the key counter-arguments have been to note (i) that the ^14^C evidence may have been compromised/affected by volcanic CO_2_, and (ii) that the ^14^C data and the calibration curve are not very accurate/precise and mid- even later-16^th^ century BCE dates also remain possible. This paper has shown that neither (i) nor (ii) apply. It is demonstrated that there is no substantive volcanic CO_2_ effect on the available ^14^C dates from the Thera VDL, and, with the availability of the IntCal20 ^14^C calibration dataset with its considerable strengthening and refinement specifically of the relevant period 1700–1500 BCE, we now have an accurate and precise ^14^C chronology for the Thera eruption from a large dataset derived from directly relevant loci. Incorporation of the known stratigraphic-temporal sequence and timing within the period immediately leading to the eruption further helps to tie the dating down. The net result is a Thera eruption date before ~1560/1550 BCE, contradicting the conventional assessment, and most likely somewhere in the last decade of the 17^th^ century BCE through first or second decades of the 16^th^ century BCE (Figs 7–12 and Tables 1–4). The final counter-argument claimed is that the archaeological evidence contradicts such an earlier chronology (e.g. [6, 8, 10, 26, 28, 29, 162–165]). However, this clearly is not the case. The scarcity and potential flexibility of the archaeological evidence for absolute chronology for the Thera period, MMIII through LMIA in the southern Aegean, is the conspicuous feature [7, 9, 11, 25, 31, 61, 98, 166–170]. The available evidence can either be re-interpreted as consistent with an earlier date, or in no way provides non-ambiguous, contradictory evidence.

It is important to observe both the substantial body of data employed in the analyses reported in this paper, and the consistent and coherent results obtained, with the data offering a good and specific fit—chronological placement—against the IntCal20 ^14^C calibration curve (Fig 9C and 9D). Entirely new and different evidence would be required to undermine the above findings and conclusions as shown in Figs 9–12 and Tables 1–4. Assuming the current ^14^C measurements, which derive from several sites and from different ^14^C laboratories, are approximately accurate and representative, then adding more similar measurements to the available datasets from Thera itself, or to the Çeşme-Bağlararası, Eşençay Delta, Palaikastro, or Trianda datasets, would likely shift the age ranges only a little at most, but might be anticipated progressively to reduce the spread of the low late probability tail into the mid-16^th^ century BCE. For example, simply including repeats of all the current data from Thera, Çeşme-Bağlararası, Eşençay Delta, Palaikastro and Trianda in a re-run ‘hypothetical’ revised Model 1 produces a 68.3% hpd range of 1605–1594 BCE and 95.4% hpd range of 1608–1586 BCE. This indicates very little real change in the most likely age, but does noticeably reduce the mid-16^th^ century BCE range. This hypothetical x2 model achieves age ranges very similar to those achieved if the Sofular Cave dates for the likely Thera-related bromine and molybdenum peaks are included into Model 1 (Fig 13).

We thus reach the conclusion that the Minoan eruption of the Thera volcano should in all likelihood be placed in the SIP. This finding is considerably more robust (and refined) than previous work. Importantly, this conclusion is also consistent with substantial evidence from other sets of ^14^C dates and dendrochronology demonstrating the need to re-think wider Mesopotamian-Levantine-Egyptian chronology and linkages in the period from the MBA to the early LBA [44, 171–173].

Overall, the weight of evidence has therefore shifted substantially across the period from the initial interventions of ^14^C into second millennium BCE Aegean chronology (e.g. [84]) to now. In the past ^14^C was not seen by many scholars to contribute usefully to second millennium BCE Aegean chronology because the archaeologically-based assessments were deemed more refined (e.g. “The radiocarbon dating evidence for Aegean chronology after about 2000 BC is for the most part less precise than dates obtainable from the Egyptian correlations”: [29] at p.127), and there was a presumption on the part of many archaeologists that Carl Sagan’s statement applied (adapting Pierre-Simon Laplace [174]), whereby “*extraordinary claims require extraordinary evidence*”, and hence ^14^C could not just arrive and change and undermine a long tradition of archaeological and art-historical interpretation. However, several decades later, a large well-replicated ^14^C dataset, a well-defined ^14^C calibration curve 1700–1500 BCE, and the application of Bayesian chronological modelling incorporating the archaeological-geological information, all combine to shift the onus now to those who would dispute the solid and refined ^14^C-based timeframe.

Beyond the date of the Thera eruption itself, the modelled findings reported imply a date range predominantly in the 17^th^ century BCE, to no later than the early/earlier 16^th^ century BC, for the majority of the LCI, LMIA and LHI cultural phases–that is the major (or total) portion of these cultural phases which occurred before the Thera eruption. This places these Aegean cultural phases—the apogee of the New Palace period on Crete and the formative period for the early Mycenaean world of southern Greece (the Shaft Grave era), along with linked cultural phases like Late Cypriot IA [4, 5, 7, 9, 11, 25, 29]—contemporary with the SIP in Egypt and later MBA of the Levant. Thus, even without definition of an exact Thera eruption date, the results reported already resolve long-running controversy over the correct dating and cultural synchronization of the Aegean, East Mediterranean, Egypt and the Levant in the LMIA period, and necessitate reassessment of conventional historical syntheses and assumptions based on the conventional low archaeological chronology for the start of the LBA in the Aegean and its supposed NK or earlier 18^th^ Dynasty context and synchronization [7, 9, 11, 31, 61, 74, 98, 166, 170–173]. Instead, the New Palace period of Crete and contemporaries in the southern Aegean are coeval with, associated with, influenced by, and a product of, the SIP/Hyksos world system. The importance of this SIP/Hyksos world, and especially of the huge regional mega-site of Avaris, cannot be over-exaggerated at this time for the whole East Mediterranean-Near East region ([35] at pp.383-386), and, especially with reference to the Aegean, has been too long ignored or excluded from scholarship [36]. “Avaris and the associated Hyksos world became *the* driving centre of trade and much else in the eastern Mediterranean over a period of towards 150 years. Rather than–as often before–looking to downplay the wide distribution of Hyksos items (from Crete to Bagdad) … it becomes more apparent that the world-system centred at Avaris was a driving force in the (long) 17^th^ century BC (end 18^th^ to early 16^th^ centuries) that formed the context for the creation of the subsequent Late Bronze Age world of the East Mediterranean” [172].

This temporal and thus culture-historical realignment requires critical re-examination and re-thinking of the myriad connections and influences across the region during the SIP, from the material to the immaterial realms. The presence of an alabaster lid with the cartouche of the Hyksos ruler Khyan at Knossos on Crete, the Nilotic imagery and a range of later Middle Bronze Age (MBA) Levantine technical and aesthetic connections in metalwork and jewelry both physically present in LCI/LHI finds from Akrotiri/Thera and Mycenae, and as represented prominently in wall paintings at Akrotiri, along with finds of later MBA Canaanite jars and Tell el-Yahudiyeh juglets at Akrotiri, and other SIP-Aegean connections (e.g. [9] at pp.55, 136–144, Figs 18–20, [25] at pp.172-175, [29] at pp.135-137, [175–177]), all take on new and different importance and meanings. Not only was there contact and trade (consumption) at these key sites but likely influence and engagement broadly across the political, social, economic and ideational realms. To give just one example of the importance of this era for history: among the key inventions and technologies of profound wider culture-historical significance likely linked with the Hyksos era is alphabetic writing, and its initial spread and development into the Levant in the later Middle Bronze Age through early Late Bronze Age [178–180], and from thence more widely across the East Mediterranean/Eurasia from the Late Bronze Age to Iron Age.

### Historiography of Thera eruption dating

The historiography of the field over the last half century reveals several stages in the process of trying to date the Thera eruption. In the 1970s there was still active debate over whether the Thera eruption and the Cretan LMIB destructions could somehow be brought together to maintain Marinatos’ 1939 hypothesis [59] that the eruption caused the Minoan destructions [58]. This was despite the increasingly clear evidence from the systematic modern excavations at Akrotiri on Thera starting in 1967 that the eruption occurred in the LMIA period, whereas the Cretan destructions were distinct and later at the close of the subsequent LMIB period (e.g. [181]). If anything, the critical pressure through this period was to test and ask whether the conventional ~1500 BCE date for the eruption (e.g. [28, 59, 84, 95]) might in fact be pushed a little later into the 15^th^ century BCE to allow as much temporal propinquity as possible [58, 182, 183]. Then ^14^C intervened, with suggestions of rather earlier dates for the Thera eruption [84, 184]. Some environmental records, from ice-cores and tree-rings that likely indicated possible major past volcanism, were also noted with suggestions made that one of these might represent the Thera eruption—in particular, events were noted in the mid-later 17^th^ century BCE [156–158]. This challenge was largely resisted by the archaeological community committed to the conventional ~1500 BCE date, with responses observing that the ^14^C evidence (at that time) was less than clear-cut and highlighting (correctly) that the supposed associations with events noted in ice-cores and tree-rings lacked any positive evidence for an association with Thera, and instead could have been any other volcanic eruption or even other cause (e.g. [28, 159]). There was, however, one important and interesting new observation: archaeological finds at Akrotiri started to indicate that at least some part of the LMIA period likely overlapped with the SIP ([185] at pp.106-107 and n.2).

Whatever the criticisms, the early ^14^C dates reported in the 1970s for Thera eruption contexts nonetheless raised an important question. Was the conventional archaeological chronology of the Aegean and east Mediterranean around the time of the Thera eruption correct? This prompted work that identified and highlighted weaknesses in the conventional archaeological orthodoxy—observing that several interpretations and assumptions were less than secure given critical study—and this began to lead to suggestions of possible changes to the Aegean chronology including an earlier date for the Thera eruption (e.g. [31, 98, 166]). The mainstream archaeological field largely moved towards a damage-limitation mode. Such views had to incorrect and were strongly opposed (e.g. [6, 8, 28, 30, 162–165]). However, at the same time, the obvious strategy was adopted, whether consciously or unconsciously, when progressively confronted especially with increasing numbers of ^14^C dates pointing to earlier ages for the eruption before ~1500 BCE, to compromise a little and suggest dates for the Thera eruption a fraction earlier while staying as close to the original ~1500 BCE date as possible. Thus dates for the eruption in the range ~1530–1500 BCE were proposed (e.g. [6, 30, 165]).

As more data became available, along with improving analytical methods, as well as on-going critical examination of both the archaeological and scientific information (which saw several initial suggestions removed), the case for re-thinking the original ~1500 BCE date kept becoming stronger despite firm opposition at each step (e.g. [4–6, 8, 10, 26, 28–30, 84, 162–165]). The key science evidence came from ^14^C. After critical scrutiny and much further investigation, no clear Thera eruption association was yet available from any of the suggested environmental proxies, and it subsequently became clear that Thera could be excluded for the volcanic signals noted ~1653 BCE and ~1628/1627 BCE [71]. By 2007, unless firmly wedded to the conventional low chronology position, it seemed only two options were possible on a critical review of data and interpretative frameworks, either (i) a new compromise chronology with the Thera eruption “in the earlier to mid-16^th^ century BC” or (ii) the ‘high’ chronology based on the then available ^14^C data and then available calibration curve with an eruption date somewhere between “1663–1599 BC” ([167] at p.125).

The last (to now) stage in the debate came with a substantial revision of the northern hemisphere ^14^C calibration curve 1700–1500 BCE, thanks to the additional input of a massive number of new modern ^14^C measurements on known-age wood across this interval (Fig 1) [12, 41, 43, 186], currently making this interval by far the best dated two century period in prehistory. This revised calibration dataset removed the possibility of a mid-17^th^ century BCE Thera date range (thus revising the previous ‘high’ chronology date range derived from the prior IntCal13 (and predecessors) ^14^C calibration curve), and instead pointed to possible dates for the Thera eruption somewhere from the late 17^th^ century through mid-16^th^ century BCE [12, 13, 42–45]—for example [12] identified a 2σ (95.4%) date range of 1614–1538 BCE. This date range potentially could include major volcanic eruption signals in various ice-core and tree-ring records from ~1611/10 BCE to ~1539 BCE [12–14, 42–44], and in general required either a revised (slightly lowered) ‘high’ chronology, or the compromise chronology of 2007—although scholars committed to the conventional ‘low’ chronology nonetheless still suggested that a date ~1525 BCE is possible (e.g. [26] at p.312), justified primarily on the basis that “1525 BC … is nearest to the archaeological range of dating” (this is despite the fact that 1525 BCE is comfortably outside the 95.4% probability range from ^14^C and therefore highly unlikely), or simply continued to adhere to a ~1500 BCE date [187].

The present paper enters the debate at this point, with its analysis reducing the 95.4% probability eruption date range to ~1609–1560 BCE (Fig 9), or ~1608–1556 BCE (Fig 12A) (or ~1610/1609–1587/78 BCE) if we incorporate the Sofular Cave bromine and molybdenum signals (Fig 13), with a most likely 68.3% range ~1606–1589 BCE (Fig 9), ~1606–1584 BCE (Fig 12A) or ~1606–1595/93 BCE (Fig 13), and, in particular, identifying that the sets of ^14^C dates directly associated with the eruption (stages (ii)/(iii) and then (v) at Akrotiri) exhibit a range/pattern in measured ^14^C ages that fits specifically against the now well-defined ^14^C calibration curve between (using the means of the modelled data) ~1644–1600 BCE with the Thera eruption date then immediately following the latter range (Fig 9C and 9D). This analysis therefore firmly excludes a Thera eruption date after ~1530 BCE and the general ‘low’ or conventional chronology interpretation and analysis, and instead points clearly to a later SIP date, most likely a date around the last decade of the 17^th^ century through the first or second decades of the 16^th^ century BCE. Further data in the future will of course lead to revision, but given that (a) the ^14^C calibration curve is now robustly defined by numerous data in this period (Fig 1), and (b) there are large sets of ^14^C data defining the stages (ii)/(iii) and stage (v) episodes and numerous other compatible ^14^C data from the Aegean region from contexts dating before, around, and after the time of the Thera eruption, it is unlikely there will be any substantial change to the approximate current best-fit region. Indeed, more data will likely only better define the date range close to or within the current 95.4% range ~1609–1560 BCE and likely close to the region of the current most likely range ~1606–1589 BCE (using the Fig 9 ranges). As noted above, if the data from the Sofular Cave are added, then such a position is reinforced and narrowed (Fig 13).

### Archaeological flexibilities

The reason there has been such a long debate over the date of the Thera eruption and the beginning of the Aegean LBA is not because of ^14^C: ^14^C merely highlighted an existing problem. This problem is that there is not, and never has been, any good, clear, set of archaeological linkages/synchronisms that tie the Aegean (Cretan) MMIII through LMIA periods to the approximate Egyptian historical chronology. Whereas there are a set of linkages for Cretan MMIB-II Kamares ware with Egyptian Middle Kingdom contexts, and then a set of linkages for material from the LMIB/LHIIA through LHIIIB periods with NK contexts [6–11, 25–30, 84, 162–168, 188], there is, as long-noted, a conspicuous gap in any secure connections during MMIII to LMIA [7, 9, 11, 84, 98, 168]. In turn, there is thus scope for considerable flexibility. This situation was recognized over 30 years ago by Hallager [169] who wrote: “it is important to stress that the renewed investigations of the traditional synchronisms of the MMIII/LMIA material have shown the contexts–both Egyptian/Near Eastern and Aegean–so dubious that a revised high chronology for the beginning of the LMIA is possible”. This situation remains the case today.

There has thus conventionally been conjecture, rather than any solid archaeological chronology, for the LMIA date range. At this point one further observation must be highlighted. Where there is a well-replicated archaeological or historical chronology directly linking contexts of interest with an historically dated timeframe (e.g. Aegean to Egypt), then modern, calibrated, ^14^C evidence has invariably supported a similar/compatible chronology and there is no major disagreement. The close of the LMIB period (Figs 10 and 11) is an example [92], and the end of the LBA offers another instance (e.g. [25, 31, 84, 189, 190]). The dating of East Mediterranean/Near Eastern historical chronologies with good evidential basis likewise yields compatible results (or, even, as resolution increases, results from ^14^C analysis that allow selection between competing historical reconstructions). The dating of the Egyptian kings is a good example [63], as is the dating of the Old Assyrian/Old Babylonian period [44, 191]. Nor are these observations peculiar to the eastern Mediterranean. The period of initial European invasion into North America offers a good example. Indigenous sites with strong, well-replicated, connections with European trade goods and their date ranges, or association with historically attested episodes (like Samuel *de Champlain spending a winter at a site)*, *yield compatible* ^14^*C dated calendar age ranges*, *whereas some sites lacking well-replicated connections and conventionally dated according only to various assumptions and interpretations may yield* ^14^*C dated ranges that challenge these past assumptions and interpretations and so call for a revised chronology (e.g. [192–195]).*

The topic of the relationship of the last part of the LHI period versus the date of the Thera eruption is a case where critical examination is necessary. Here it is important to note that several of the pieces of archaeological evidence previously adduced to try to dispute a higher date for the Thera eruption (that is a date during the SIP and before the NK) can no longer be regarded as sound or unambiguous (e.g. [7, 9, 11]). In particular, two items should be noted. First: the last stage of LHI is not represented at Akrotiri before the eruption [196], and a late-LHI ^14^C dataset (k-2) from Lerna Shaft Grave 2 in fact suggests late-LHI may continue a little later into the 16^th^ century BCE (Fig 10). Thus it is possible that a few elements may occur before the very end of LHI that post-date the Thera eruption and these specifically do not offer a TPQ for the eruption date. Second: diagnosis of some late SIP versus early NK (18^th^ Dynasty) vessel types (especially stone vessels) is difficult and not securely based, undermining past use of an NK TPQ for a point in later LHI or LCI based on such items ([7] at pp.440-441, [9] at RE pp.37-38, [11] at pp.1165-1167] (and note the previous point, regardless).

Overall, the field has reached a turning point in the search for a date for the Thera eruption. From the ^14^C side the evidence available is plural, plentiful and coherent, and, critically, the calibration curve is now very much better defined and revises past positions. Previous criticisms have been overcome, or no longer apply. Of course, those who, nevertheless, are committed to supporting the conventional, low, chronology for the Aegean and eastern Mediterranean will point to some archaeologically based observations or interpretations as supposedly disproving the possibility of the higher ^14^C-based chronology (e.g. [6, 8, 26, 30, 162–165]), and will dispute arguments demonstrating that such evidence is in fact either ambiguous or lacks chronological precision or is capable of different interpretations. Of course, the simple fact that such debate has continued for several decades highlights the fundamental weakness of this opposition and the lack of any solid (positive) evidence in support of its case. Indeed, the strongest opposition tactic of last resort was not drawn from archaeology, but to make arguments that the ^14^C evidence was potentially ambiguous or not reliable, and could not really be trusted because it might be biased by volcanic CO_2_ (e.g. [6, 8, 26, 165, 197]). However, this paper has demonstrated that these concerns no longer apply. This leaves nothing sound—only belief and tradition—that contradicts the findings reported in this paper pointing to a SIP date for the Thera eruption.

Assessed objectively, the likely Thera eruption date range identified in this paper, between past ‘high’ (1640s, 1620s BCE) and ‘low’ (1530–1500 BCE) positions, usefully facilitates a new consensus that can overcome many previous archaeological objections. As an example, and perhaps presciently, Merrillees in a paper of 2009 remarked that “a lowering of this event [the Thera eruption] closer to the end of the 17^th^ century BC would fit … more comfortably, as well as satisfy me” ([198] at p.251). We now have a most likely 68.3% dating range ~1606–1589 BCE (Fig 9). In support, other Aegean ^14^C datasets, (i)–(l), provide compatible calendar placements: early to mature LMIA and early to mid-LHI in the 17^th^ century BCE, late-LHI (including Mycenae Shaft Grave Circle A [199]) in the late 17^th^ to earlier 16^th^ century BCE, and (we lack any earlier LMIB dataset) later/close LMIB in the earlier to mid-15^th^ centuries BCE (Figs 10 and 12 and Tables 1–5). At the same time, the archaeologically-based site chronology of Avaris/Tell el-Dab‘a, and the linked/derived Levantine MBA to early LBA timeframe, long deployed as an obstacle to a higher/earlier chronology for the Thera eruption and the start of the Aegean LBA by Bietak (e.g. [8, 26, 200]), have also been shown both to lack solid archaeological-historical foundation and to be contradicted by extensive ^14^C evidence that instead offers a coherent chronological framework consistent with the ^14^C timeframe in the Aegean (e.g. [7, 9, 11, 44, 170–172, 201]).

**Table 5:** Modelled results (bold = 68.3% hpd, and non-bold = 95.4% hpd) for the datasets shown in Fig 10. The majority range where there are two or more split ranges is underlined.

Dataset	68.3% hpd Dates BCE	95.4% hpd Dates BCE
**Dataset I, Kolonna J/K or MH/LHI transition**	**1701–1654 (45.0)** **1649–1618 (23.3)**	1744–1582
**Dataset j Kommos Early LMIA Date (twig)**	**1731–1727 (3.9)** **1694–1636 (64.3)**	1741–1710 (19.4) 1700–1622 (76.0)
**Dataset k-1 Lerna Shaft Grave 1 Mid-LHI**	**1666–1649 (20.0)** **1638–1608 (48.3)**	1726–1718 (1.2) 1686–1592 (94.3)
**Akrotiri Stages (ii)/(iii)**	**1610–1592**	1614–1562
**Thera Eruption (TE5)**	**1606–1589**	1609–1560
**Dataset k-2 Lerna Shaft Grave 2 Late-LHI**	**1606–1557 (65.4)** **1553–1552 (1.2)** **1550–1548 (1.8)**	1611–1531
**Dataset l Chania LMIB Destruction**	**1504–1452**	1526–1421
**Dataset l Myrtos-Pyrgos LMIB Late Destruction**	**1499–1486 (21.3)** **1478–1452 (47.0)**	1505–1437
**Dataset l Mochlos LMIB Final Destruction**	**1467–1426**	1491–1398

### Thera and the LMIB destructions on Crete

The comparison in Fig 11 of the date ranges for the close of LMIB destructions on Crete versus the Thera eruption indicates a lengthy time interval between these episodes, likely >100 years. Such a long temporal separation makes it challenging to articulate any direct causal association, although the long-term systemic impacts of the evisceration of Thera, and what had been a major port and hub in the Aegean region until that time, will have significantly impacted the whole area, and necessarily led to substantial reconfiguration of regional networks and histories [3, 17, 202]. More salient: this long interval and, at present, the lack of dated contexts on Crete for the period ~1580/50–1500 BCE, along with, for instance, indications of disruption and partial abandonment at Trianda on Rhodes in the period after the Theran tephra fall [71], and changes and much reduced Minoan associations at Iasos, Caria, in western Turkey in the period after Thera tephra fall [203], highlights the question of whether there was an, as yet, largely undocumented period of major, sustained, disruption on Crete (and affected parts of the Aegean) associated with the period of the Minoan Thera volcanic eruption, starting in late/end LMIA perhaps associated with the major earthquake evident on Thera and felt in Crete and then the enormous eruption itself [1–3, 17, 49, 56, 60]. Despite a lack of clear regional environmental evidence for Thera eruption impacts in pollen records from northwest Crete and southwest Turkey [204, 205], in light of the increasing evidence for disruptions in parts of the southern Aegean, and increasing evidence for devastating Thera tsunami impacts in the Aegean [3, 45, 52–54, 56, 73], and indications of complicated processes of late LMIA abandonment, tephra fall, and flooding as observed in east Crete [206], attention focuses onto a potentially culturally transformative episode—mainly marked at present by an absence of evidence—in the earlier to mid-16^th^ century BC. This needs more investigation with analysis and dating of initial to earlier LMIB contexts. The subsequent appearance and wide but selective distribution of the notable Marine Style motifs of the Special Palatial Tradition in later LMIB, and the associations of these with ritual contexts, is conspicuous [95, 207, 208], and may offer one reflection of, and even ideological statement referencing, the long-reaching events (seismic, volcanic, and *tsunamogenic)*, trauma, and changes in the southern Aegean that resulted directly and indirectly from the enormous Thera eruption [3, 17, 45, 49, 56, 60, 199, 208].

## Supporting information

S1 Table The ^14^C dates employed in this study in four parts: S1A Table, S1B Table, S1C Table and S1D Table. (DOCX)

S2 Table OxCal runfiles for the models used in this paper. (DOCX)

S1 File Comments on the individual datasets and their modelling. (DOCX)

S2 File Checklist–additional information regarding the ethical, cultural, and scientific considerations specific to inclusivity in global research. (DOCX)

S1 Fig Results from a re-run of Model 1 (Fig 9) without the VERA-4630 TAQ—see Fig 9 caption for description—showing model run 1 from Table 2. **(A)** Results for the end of Stages (ii)/(iii) Boundary and the Thera Eruption Boundary from Model 1 run 1 with log-normal, LnN(ln(3),ln(2)), constraint applied (Table 2), detailing the 68.3% and 95.4% hpd calendar age ranges. **(B)** Modelled posterior (solid, cyan) probability versus the log-normal prior (hollow distribution) for the Difference constraint.
(JPG)

S2 Fig Two alternative versions of Model 1 (as in Fig 9) and the modelled Thera Eruption (stage v) Boundary. **(A)** Model 1 (with VERA-4630 TAQ) as in Fig 9 and reported in Table 1 but re-run instead with a more compressed/shorter LnN(ln(0.75),ln(3)) constraint on the Difference query for the time interval between stages (ii)/(iii) and stage (v). The modelled Thera Eruption (stage v) Boundary determined is very similar to the result reported with the slightly looser LnN(ln(3),ln(2)) constraint in the main text (Fig 9 and Table 1). Note: the INSET shows the modelled posterior (solid, cyan) probability versus the log-normal prior (hollow distribution) for this Difference constraint and shows good agreement (98.0%) for this more compressed/shorter constraint also (compare Fig 9B). **(B)** Model 1 (with VERA-4630 TAQ) as in Fig 9 and reported in Table 1 re-run adding two Thera eruption TPQ ^14^C dates from Gölhisar Gölu. The modelled Thera Eruption (stage v) Boundary is very similar to that shown in Fig 9 and the values reported for this Boundary from multiple runs of the Fig 9 model in Table 1.
(JPG)

S3 Fig (JPG)

S4 Fig (JPG)

## References

[1] Johnston EN, Sparks RSJ, Phillips JC, Carey S. Revised estimates for the volume of the Late Bronze Age Minoan eruption, Santorini, Greece. Journal of the Geological Society, London 2014; 171:583–590.

[2] Friedrich WL. Santorini: volcano, natural history, mythology. Aarhus: Aarhus University Press; 2009.

[3] Driessen J. The Santorini eruption. An archaeological investigation of its distal impacts on Minoan Crete. Quaternary International 2019; 499:195–204.

[4] Hardy DA, Renfrew AC, editors. Thera and the Aegean world III. Volume three: chronology. London: The Thera Foundation; 1990.

[5] Warburton DA, editor. Time’s Up! Dating the Minoan eruption of Santorini. Athens: The Danish Institute at Athens; 2009.

[6] Wiener MH. A point in time. In Krzyszkowska O, editor, Cretan Offerings. Studies in Honour of Peter Warren. BSA Studies 18. London: The British School at Athens; 2010. pp.367–394.

[7] Höflmayer F. The date of the Minoan Santorini eruption: quantifying the ‘offset’. Radiocarbon 2012; 54:435–448.

[8] Bietak M. Antagonisms in historical and radiocarbon chronology. In Shortland AJ, Bronk Ramsey C, editors, Radiocarbon and the Chronologies of Ancient Egypt. Oxford: Oxbow Books; 2013. pp.78–110.

[9] Manning SW. A Test of Time and A Test of Time Revisited. The Volcano of Thera and the chronology and history of the Aegean and east Mediterranean in the mid-second millennium BC. Oxford: Oxbow Books; 2014.

[10] Antiquity. Bronze Age catastrophe and modern controversy: dating the Santorini eruption. Antiquity 2014; 88:267–291.

[11] Manning SW et al. Dating the Thera (Santorini) eruption: archaeological and scientific evidence supporting a high chronology. Antiquity 2014; 88:1164–1179

[12] Pearson CL et al. Annual radiocarbon record indicates 16^th^ century BCE date for the Thera eruption. Science Advances 2018; 4:eaar8241. 10.1126/sciadv.aar8241 30116779 PMC6093623

[13] Pearson C. Re-thinking Thera: tree-rings, radiocarbon, and response in the second millennium BCE. In Roosevelt CH, Haldon J, editors, Winds of Change: Environment and Society in Anatolia. Istanbul: Koç University Press; 2021. pp.161–186.

[14] Pearson C. et al. Geochemical ice-core constraints on the timing and climatic impact of Aniakchak II (1628 BCE) and Thera (Minoan) volcanic eruptions. PNAS Nexus 2022; 1: pgac048. 10.1093/pnasnexus/pgac048 PMC9802406 36713327

[15] Hägg R, Marinatos N, editors. The Minoan Thalassocracy, Myth and Reality: proceedings of the third international symposium at the Swedish Institute in Athens, 31 May-5 June, 1982. Stockholm: Svenska institutet i Athen; 1984.

[16] Wiener MH. The isles of Crete? The Minoan thalassocracy revisited. In Hardy DA, Doumas CG, Sakellerakis JA, Warren PM, editors, Thera and the Aegean World III. Volume one: archaeology. London: The Thera Foundation; 1990. pp.128–161.

[17] Driessen J, Macdonald CF. The troubled island: Minoan Crete before and after the Santorini eruption. Aegaeum 17. Liège: Université de Liège and University of Texas at Austin; 1997.

[18] Rehak P, Younger JG. Review of Aegean Prehistory VII: Neopalatial, Final Palatial, and Postpalatial Crete. American Journal of Archaeology 1998; 102:91–173.

[19] Younger JG., Rehak P. The material culture of Neopalatial Crete. In Shelmerdine CW, editor, The Cambridge Companion to the Aegean Bronze Age. Cambridge: Cambridge University Press; 2008. pp.140–164.

[20] Whitelaw T. Feeding Knossos: exploring economic and logistical implications of urbanism on prehistoric Crete. In Garcia D, Orgeolet R, Pomadère M, Zurbach J, editors, Country in the City: Agricultural Functions of Protohistoric Urban Settlements (Aegean and Western Mediterranean). Oxford: Archaeopress; 2020. pp. 88–121.

[21] Broodbank C. An Island Archaeology of the Early Cyclades. Cambridge: Cambridge University Press; 2000.

[22] Duhoux Y. Le linéaire A: problèmes de déchiffrement. In Duhoux Y, Palaima TG, Bennet J editors, Problems in Decipherment. Bibliothèque des cahiers de l’Institut de Linguistique de Louvain 49. Louvain-La-Neuve: Peeters; 1989. pp.59–119.

[23] Finkelberg M. The language of Linear A. In Drews R, editor, Greater Anatolia and the Indo-Hittite Language Family. Washington DC: Institute for the Study of Man; 2001. pp.81–105.

[24] Salgarella E. 2020. Aegean Linear Script(s): Rethinking the Relationship between Linear A and Linear B. Cambridge: Cambridge University Press; 2020.

[25] Höflmayer F. 2012. Die Synchronisierung der minoischen Alt- und Neupalastzeit mit der ägyptischen Chronologie. Wien: Verlag der Österreichischen Akademie der Wissenschaften; 2012.

[26] Bietak M. Recent Discussions about the Chronology of the Middle and the Late Bronze Ages in the Eastern Mediterranean: Part II. The End of High Chronology in the Aegean and the Levant? Bibliotheca Orientalis 2021; 78(3–4):282–318.

[27] Popham MR. Late Minoan Chronology. American Journal of Archaeology 1970; 74:226–228.

[28] Warren P. Absolute Dating of the Bronze Age Eruption of Thera (Santorini). Nature 1984; 308:492–493.

[29] Warren P, Hankey V. Aegean Bronze Age Chronology. Bristol: Bristol Classical Press; 1989.

[30] Warren P. The Date of the Late Bronze Age Eruption of Santorini on the Basis of the Historical Chronology. Pasiphae 2010; 4:67–72.

[31] Betancourt PP. Dating the Aegean Late Bronze Age with Radiocarbon. Archaeometry 1987; 29:45–49.

[32] Oren E, editor. The Hyksos: new historical and archaeological perspectives. Philadelphia: University Museum, University of Pennsylvania; 1997.

[33] Bourriau J. The Second Intermediate Period (c.1650–1550 BC). In Shaw I, editor, The Oxford History of Ancient Egypt. Oxford: Oxford University Press; 2000. pp.184–217.

[34] Marée M, editor. The Second Intermediate Period (thirteenth-seventeenth dynasties): current research, future prospects. Leuven: Peeters; 2010.

[35] Broodbank C. The Making of the Middle Sea: A History of the Mediterranean from the Beginning to the Emergence of the Classical World. Oxford: Oxford University Press; 2013.

[36] Bernal M. Black Athena: The Afroasiatic Roots of Classical Civilization. Volume I: The Fabrication of Ancient Greece 1785–1985. New Brunswick, NJ: Rutgers University Press; 1987.

[37] Van Seters J. The Hyksos: a new investigation. New Haven: Yale University Press; 1966.

[38] Bietak M. Avaris: The Capital of the Hyksos. London: British Museum Press; 1996.

[39] Bietak M. Houses, Palaces and Development of Social Structure in Avaris. In Bietak M, Czerny E, Forstner-Müller I, editors, Cities and Urbanism in Ancient Egypt: Papers from a Workshop in November 2006 at the Austrian Academy of Sciences. Wien: Verlag der Österreichischen Akademie der Wissenschaften; 2010. pp.11–68.

[40] Cline EH. Rich beyond the dreams of Avaris: Tell el-Dab‘a and the Aegean world—a guide for the perplexed. Annual of the British School at Athens 1998; 93:199–219.

[41] Reimer PJ et al. The IntCal20 Northern Hemisphere radiocarbon age calibration curve (0–55 kcal BP). Radiocarbon 2020; 62:725–757.

[42] Pearson C, Salzer M, Wacker L, Brewer P, Sookdeo A, Kuniholm P. Securing timelines in the ancient Mediterranean using multiproxy annual tree-ring data, Proceedings of the National Academy of Sciences of the United States of America 2020; 117:8410–8415. doi: 10.1073/pnas.1917445117 32229554 PMC7165418

[43] van der Plicht J, Bronk Ramsey C, Heaton T, Scott E, Talamo S. Recent developments in calibration for archaeological and environmental samples. Radiocarbon 2020; 62:1095–1117.

[44] Manning SW et al. Radiocarbon offsets and old world chronology as relevant to Mesopotamia, Egypt, Anatolia and Thera (Santorini). Scientific Reports 2020; 10: 41598. 10.1038/s41598-020-69287-2 PMC7431540 32807792

[45] Şahoğlu V et al. Volcanic ash, victims, and tsunami debris from the Late Bronze Age Thera eruption discovered at Çeşme-Bağlararası (Turkey). Proceedings of the National Academy of Sciences of the United States of America 2021; 119: e2114213118. 10.1073/pnas.2114213118 PMC8740722 34969845

[46] Palyvou C. Akrotiri Thera: An Architecture of Affluence 3,500 Years Old. Philadelphia: INSTAP Academic Press; 2005.

[47] Karátson D et al. Constraining the landscape of Late Bronze Age Santorini prior to the Minoan eruption: insights from volcanological, geomorphological and archaeological findings. Journal of Volcanology and Geothermal Research 2020; 401:106911. 10.1016/j.jvolgeores.2020.106911.

[48] Evans KJ, McCoy FW. Precursory eruptive activity and implied cultural responses to the Late Bronze Age (LBA) eruption of Thera (Santorini, Greece). Journal of Volcanology and Geothermal Research 2020; 397:106868. 10.1016/j.jvolgeores.2020.106868

[49] Driessen J, Macdonald CF. The eruption of the Santorini volcano and its effects on Minoan Crete. In McGuire WJ, Griffiths DR, Hancock PL, Stewart IS, editors, The Archaeology of Geological Catastrophes. Special Publications 171. London: Geological Society; 2000. pp.81–93.

[50] Doumas CG, Palyvou C, Devetzi A, Boulotis C. Akrotiri, Thera 17th century BC: a cosmopolitan town 3500 years ago. Athens: Society for the Promotion of Studies on Prehistoric Thera; 2015.

[51] Doumas C. Prehistoric Thera. Athens: John S. Latsis Public Benefit Foundation, Athens; 2016.

[52] Bruins HJ et al. Geoarchaeological tsunami deposits at Palaikastro (Crete) and the Late Minoan IA eruption of Santorini. Journal of Archaeological Science 2008; 35:191–212.

[53] Lespez L et al. Discovery of a tsunami deposit from the Bronze Age Santorini eruption at Malia (Crete): impact, chronology, extension. Scientific Reports 2021; 11: 15487 doi: 10.1038/s41598-021-94859-1 34326405 PMC8322394

[54] Aydar E, Çiner A, Ersoy O, Écochard E, Fouache EG. Volcanic ash and tsunami record of the Minoan Late Bronze Age Eruption (Santorini) in a distal setting, southwestern Turkey. Journal of Quaternary Science 2021; 36:586–597.

[55] Watkins N et al. Volume and extent of the Minoan tephra from Santorini Volcano: new evidence from deep-sea sediment cores. Nature 1978; 271:122–126.

[56] McCoy FW, Heiken G. 2000. The Late-Bronze Age explosive eruption of Thera (Santorini), Greece: Regional and local effects. In McCoy FW, Heiken G, editors, Volcanic Hazards and Disasters in Human Antiquity. Geological Society of America Special Paper 345, Boulder, Colorado: The Geological Society of America; 2000. pp.43–70.

[57] Bronk Ramsey C. Bayesian analysis of radiocarbon dates. Radiocarbon 2009; 51:337–360.

[58] Page DL. 1970. The Santorini Volcano and the Destruction of Minoan Crete. London: Society for the Promotion of Hellenic Studies; 1970.

[59] Marinatos S. The Volcanic Destruction of Minoan Crete. Antiquity 1939; 13:425–439.

[60] Manning SW. The volcano of Thera and the destruction of Minoan Crete. Kretika Chronika 1987; 26:59–85.

[61] Ritner RK, Moeller N. The Ahmost ‘Tempest Stela’, Thera and Comparative Chronology. Journal of Near Eastern Studies 2014; 73:1–19.

[62] Gautschy R. A reassessment of the absolute chronology of the Egyptian New Kingdom and its ‘brotherly’ countries. Ägypten und Levante 2014; 24:141–158.

[63] Bronk Ramsey C et al. Radiocarbon-based chronology for Dynastic Egypt. Science 2010; 328:1554–1557. doi: 10.1126/science.1189395 20558717

[64] Bietak M, Prell S, editors. The Enigma of the Hyksos. Volume I, ASOR Conference Boston 2017, ICAANE Conference Munich 2018, Collected Papers. Wiesbaden: Harrassowitz Verlag; 2019.

[65] Stantis C et al. Who were the Hyksos? Challenging traditional narratives using strontium isotope (87Sr/86Sr) analysis of human remains from ancient Egypt. PLoS ONE 2020; 15:e0235414. doi: 10.1371/journal.pone.0235414 32667937 PMC7363063

[66] Michael HN. Radiocarbon dates from the site of Akrotiri, Thera, 1967–1977. In Doumas C, editor, Thera and the Aegean world I. London: Thera and the Aegean World; 1978. pp.791–795.

[67] Housley RA, Hedges REM, Law IA, Bronk Ramsey C. Radiocarbon dating by AMS of the destruction of Akrotiri. In Hardy DA, Renfrew AC, editors, Thera and the Aegean world III. Volume three: chronology. London: The Thera Foundation; 1990. pp.207–215.

[68] Soter S. Radiocarbon anomalies from old CO2 in the soil and canopy air. Radiocarbon 2011; 53:55–69.

[69] Manning SW, Kromer B. Considerations of the scale of radiocarbon offsets in the east Mediterranean, and considering a case for the latest (most recent) likely date for the Santorini eruption. Radiocarbon 2012; 54:449–474.

[70] McAneney J, Baillie M. Absolute tree-ring dates for the Late Bronze Age eruptions of Aniakchak and Thera in light of a proposed revision of ice-core chronologies. Antiquity 2019; 93:99–112.

[71] Marketou T. Santorini tephra from Rhodes and Kos: some remarks based on the stratigraphy. In Hardy DA, Renfrew AC, editors, Thera and the Aegean world III. Volume three: chronology. London: The Thera Foundation; 1990. pp.100–113.

[72] Marketou T, Facorellis Y, Maniatis Y. New Late Bronze Age chronology from the Ialysos region, Rhodes. Mediterranean Archaeology and Archaeometry 2001; 1:19–29.

[73] Bruins HJ, van der Plicht J, MacGillivray JA. The Minoan Santorini eruption and tsunami deposits in Palaikastro (Crete): dating by geology, archaeology, 14C, and Egyptian chronology. Radiocarbon 2009; 51:397–411.

[74] Manning SW et al. Chronology for the Aegean Late Bronze Age. Science 2006; 312:565–569. 16645092 10.1126/science.1125682

[75] Friedrich WL, Friborg R, Tauber H. Two radiocarbon dates of the Minoan eruption on Santorini (Greece). In Doumas C, editor, Thera and the Aegean World II. London: Thera and the Aegean World; 1980. pp.241–243.

[76] Friedrich WL, Wagner P, Tauber H, Radiocarbon dated plant remains from Akrotiri Excavations on Santorini, Greece. In Hardy DA, Renfrew AC, editors, Thera and the Aegean world III. Volume three: chronology. London: The Thera Foundation; 1990. pp.188–196.

[77] Hubberten H.-W, Bruns M, Calamiotou M, Apostolakis C, Filippakis S, Grimanis A. Radiocarbon dates from the Akrotiri excavations. In Hardy DA, Renfrew AC, editors, Thera and the Aegean world III. Volume three: chronology. London: The Thera Foundation; 1990. pp.179–187.

[78] Maniatis Y. Radiocarbon dating of the Late Cycladic building and destruction phases at Akrotiri, Thera: new evidence. The European Physical Journal Plus 2012; 127:9. 10.1140/epjp/i2012-12009-y

[79] Panagiotakopulu E, Higham T, Sarpaki A, Buckland P, Doumas C. Ancient pests: the season of the Santorini Minoan volcanic eruption and a date from insect chitin. Naturwissenschaften 2013; 100:683–689. doi: 10.1007/s00114-013-1068-8 23793358

[80] Friedrich WL, Kromer B, Friedrich M, Heinemeier J, Pfeiffer T, Talamo S. Santorini eruption radiocarbon dated to 1627–1600 B.C. Science 2006; 312:548. doi: 10.1126/science.1125087 16645088

[81] Heinemeier J, Friedrich WL, Kromer B, Bronk Ramsay C. The Minoan eruption of Santorini radiocarbon dated by an olive tree buried by the eruption. In Warburton DA, editor, Time’s Up! Dating the Minoan eruption of Santorini. Aarhus: Aarhus University Press; 2009. pp.285–293.

[82] Wild EM, Steier P, Fischer P, Höflmayer F. ^14^C dating of humic acids from Bronze and Iron Age plant remains from the eastern Mediterranean. Radiocarbon 2013; 55:599–607.

[83] Broecker WS, Olson EA. Lamont radiocarbon measurements VI. Radiocarbon 1959; 1:111–132.

[84] Betancourt PP, Weinstein GA. Carbon-14 and the beginning of the Late Bronze Age in the Aegean. American Journal of Archaeology 1976; 80:329–348.

[85] Fishman B, Forbes H, Lawn B. University of Pennsylvania Radiocarbon dates XIX. Radiocarbon 1977; 19:188–228.

[86] Fishman B, Lawn B. University of Pennsylvania radiocarbon dates XX. Radiocarbon 1978; 20:210–233.

[87] Meulengracht A, McGovern P, Lawn B. University of Pennsylvania radiocarbon dates XXI. Radiocarbon 1981; 23:227–240.

[88] Kutschera W, Stadler P. 14C dating for absolute chronology of Eastern Mediterranean cultures in the second millennium BC with accelerator mass spectrometry. In Bietak M, editor, The Synchronisation of Civilizations in the Second Millennium B.C. Contributions to the Chronology of the Eastern Mediterranean I. Wien: Verlag der Österreichischen Akademie der Wissenschaften; 2000. pp.68–81.

[89] Wild EM et al. ^14^C dating of the Early to Late Bronze Age stratigraphic sequence of Aegina Kolonna, Greece. Nuclear Instruments and Methods in Physics Research B 2010; 268:1013–1021.

[90] Manning SW, Bronk Ramsey C. The dating of the earlier Late Minoan IA period: a brief note. In Warburton DA, editor, Time’s Up! Dating the Minoan eruption of Santorini. Aarhus: Aarhus University Press; 2009. pp.227–245.

[91] Lindblom M, Manning SW. The chronology of the Lerna Shaft Graves. In Gauß W, Lindblom M, Smith RAK, Wright JC, editors, Our Cups Are Full: Pottery and Society in the Aegean Bronze Age. Papers presented to Jeremy B. Rutter on the occasion of his 65th birthday. Oxford: Archaeopress; 2011. pp.140–153.

[92] Manning SW. Beyond the Santorini eruption: some notes on dating the Late Minoan IB period on Crete, and implications for Cretan-Egyptian relations in the 15th century BC (and especially LMII). In Warburton DA, editor, Time’s Up! Dating the Minoan eruption of Santorini. Aarhus: Aarhus University Press; 2009. pp. 207–226.

[93] Rutter JB. Late Minoan IB at Kommos: a sequence of at least three distinct stages. In Brogan TM, Hallager E, editors, LM IB pottery: relative chronology and regional differences. Athens: The Danish Institute at Athens; 2011. pp.307–343.

[94] Nelson DE, Vogel JS, Southon JR. Another suite of confusing radiocarbon dates for the destruction of Akrotiri. In Hardy DA, Renfrew AC, editors, Thera and the Aegean world III. Volume three: chronology. London: The Thera Foundation; 1990. pp.197–206.

[95] Popham MR. Pottery styles and chronology. In Hardy DA, Renfrew AC, editors, Thera and the Aegean world III. Volume three: chronology. London: The Thera Foundation; 1990. pp.27–28.

[96] Brogan TM, Hallager E, editors. LM IB pottery: relative chronology and regional differences. Athens: The Danish Institute at Athens; 2011.

[97] Bruins HJ, Keller J, Klügel A, Kisch HJ, Katra I, van der Plicht J. Tephra in caves: distal deposits of the Minoan Santorini eruption and the Campanian super-eruption. Quaternary International 2019; 499:135–147.

[98] Manning SW. The Bronze Age eruption of Thera: absolute dating, Aegean chronology and Mediterranean cultural interrelations. Journal of Mediterranean Archaeology 1988; 1:17–82.

[99] Housley RA, Manning SW, Cadogan G, Jones RE, Hedges REM. Radiocarbon, calibration, and the chronology of the Late Minoan IB phase. Journal of Archaeological Science 1999; 26:159–171.

[100] Engstrand LG, Stockholm natural radiocarbon measurements VI. Radiocarbon 1965; 7:257–290.

[101] Stuckenrath R Jr, Lawn B. University of Pennsylvania radiocarbon dates XI. Radiocarbon 1969; 11:150–162.

[102] Cummer WW, Schofeld E. Keos III, Ayia Irini: House A. Mainz: Phillip von Zabern; 1984.

[103] Trájer AJ. A modelling approach to the investigation of the effects of the Minoan supervolcanic eruption on Aegean sand fly diversity. The Holocene 2021; 31:1593–1608.

[104] Doumas C. Archaeological observations at Akrotiri relating to the volcanic destruction. In Hardy DA, Renfrew AC, editors, Thera and the Aegean world III. Volume three: chronology. London: The Thera Foundation; 1990. pp.48–50.

[105] Limbrey S, Soil studies at Akrotiri. In Hardy DA, Keller J, Galanopoulos VP, Flemming NC, Druitt TH, editors, Thera and the Aegean world III. Volume two: earth sciences. London: The Thera Foundation; 1990. pp.377–383.

[106] Bruns M, Levin I, Münnich KO, Hubberten HW, Fillipakis S. Regional sources of volcanic carbon dioxide and their influence on 14C content of present-day plant material. Radiocarbon 1980; 22:532–536.

[107] Pasquier-Cardin A. et al. Magma-derived CO_2_ emissions recorded in ^14^C and ^13^C content of plants growing in Furnas caldera, Azores. Journal of Volcanology and Geothermal Research 1999; 92:195–207.

[108] Cook AC, Hainsworth LJ, Sorey ML, Evans WC, Southon JR. Radiocarbon studies of plant leaves and tree rings from Mammoth Mountain, CA: a long-term record of magmatic CO_2_ release. Chemical Geology 2001; 177:117–131.

[109] Evans WC, Bergfeld D, McGeehin JP, King JC, Heasler H. Tree-ring ^14^C links seismic swarm to CO_2_ spike at Yellowstone, USA. Geology 2010; 38:1075–1078.

[110] D’Arcy F, Boucher É, De Moor JM, Hélie J-F, Piggott R, Stix J. Carbon and sulfur isotopes in tree rings as a proxy for volcanic degassing. Geology 2019; 47:825–828.

[111] Seiler R. et al. Tree-ring stable isotopes and radiocarbon reveal pre- and post-eruption effects of volcanic processes on trees on Mt. Etna (Sicily, Italy). Ecohydrology 2021; 14:e2340.

[112] Hogg AG. et al. Wiggle-match radiocarbon dating of the Taupo eruption. Nature Communications 2019; 10:4669. doi: 10.1038/s41467-019-12532-8 31604909 PMC6788995

[113] Holdaway RN, Duffy B, Kennedy B. Reply to ‘Wiggle-match radiocarbon dating of the Taupo eruption’. Nature Communications 2019; 10:4668. 10.1038/s41467-019-12491-0 PMC6789094 31604918

[114] Flaherty T, Druitt TH, Tuffen H, Higgins MD, Costa F, Cadoux A. Multiple timescale constraints for high-flux magma chamber assembly prior to the Late Bronze Age eruption of Santorini (Greece). Contributions to Mineralogy and Petrology 2018; 173:75. 10.1007/s00410-018-1490-1

[115] Dee MW et al. Investigating the likelihood of a reservoir offset in the radiocarbon record for ancient Egypt. Journal of Archaeological Science 2010; 37:687–693.

[116] Manning SW et al. Fluctuating radiocarbon offsets observed in the southern Levant and implications for archaeological chronology debates. Proceedings of the National Academy of Sciences of the United States of America 2018; 115:6141–6146. doi: 10.1073/pnas.1719420115 29844183 PMC6004441

[117] Manning SW et al. Mediterranean radiocarbon offsets and calendar dates for prehistory. Science Advances 2020; 6:eaaz1096. doi: 10.1126/sciadv.aaz1096 32206721 PMC7080444

[118] Nydal R, Lövseth K. Tracing bomb 14C in the atmosphere 1962–1980. Journal of Geophysical Research 1983; 88 C6:3621–3642.

[119] Levin I, Kromer B. The tropospheric 14CO2 level in mid-latitudes of the Northern Hemisphere (1959–2003). Radiocarbon 2004; 46:1261–1272.

[120] Braziunas TF, Fung IY, Stuiver M. The preindustrial atmospheric ^14^CO_2_ latitudinal gradient as related to exchanges among atmospheric, oceanic, and terrestrial reservoirs. Global Biogeochemical Cycles 1995; 9:565–584.

[121] Büntgen U et al. Tree rings reveal globally coherent signature of cosmogenic radiocarbon events in 774 and 993 CE. Nature Communications 2018; 9:3605. doi: 10.1038/s41467-018-06036-0 30190505 PMC6127282

[122] Hua Q et al. Atmospheric radiocarbon for the period 1950–2019. Radiocarbon 2021; 64:723–745. 10.1017/RDC.2021.95

[123] Levin I et al. Observations and modelling of the global distribution and long-term trend of atmospheric 14CO2. Tellus B 2010;62:26–46.

[124] Randerson JT, Enting I, Schuur EAG, Caldiera K, Fung IY. Seasonal and latitudinal variability of troposphere Δ^14^CO_2_: Post bomb contributions from fossil fuels, oceans, the stratosphere, and the terrestrial biosphere. Global Biogeochemical Cycles 2002; 16:1112. https://doi:10.1029/2002GB001876.

[125] Graven HD, Guilderson TP, Keeling RF. 2012. Observations of radiocarbon in CO_2_ at seven global sampling sites in the Scripps flask network: Analysis of spatial gradients and seasonal cycles. Journal of Geophysical Research 2012; 117:D02303. 10.1029/2011JD016535.

[126] Halstead P. Two Oxen Ahead: Pre-Mechanized Farming in the Mediterranean. Chichester: John Wiley & Sons; 2014.

[127] Moriondo M et al. Assessing climate change impacts on crops by adopting a set of crop performance indicators. Euro-Mediterranean Journal for Environmental Integration 2021; 6:45. 10.1007/s41207-021-00246-7.

[128] Borowski O. Agriculture in Iron Age Israel. Winona Lake: Eisenbrauns; 1987.

[129] Hartmann F. L’agriculture dans l’ancienne Égypte. Paris: Librairies-imprimeries réunies; 1923.

[130] Haneca K Čufar K, Beeckman H. Oaks, tree-rings and wooden cultural heritage: a review of the main characteristics and applications of oak dendrochronology in Europe. Journal of Archaeological Science 2009; 36:1–11.

[131] Bayliss A, Marshall P, Dee M, Friedrich M, Heaton T, Wacker L. IntCal20 tree rings: an archaeological Swot Analysis. Radiocarbon 2020; 62:1045–1078.

[132] Ward GK, Wilson SR. Procedures for comparing and combining radiocarbon age determinations: A critique. Archaeometry 1978; 20:19–31.

[133] Buck CE, Cavanagh WG, Litton CD. Bayesian approach to interpreting archaeological data. Chichester: Wiley; 1996.

[134] Bayliss A. Rolling out revolution: using radiocarbon dating in archaeology. Radiocarbon 2009; 51:123–147.

[135] Hamilton W, Krus A. 2018. The myths and realities of Bayesian chronological modeling revealed. American Antiquity 2018; 83:187–203.

[136] Bronk Ramsey C. Radiocarbon calibration and analysis of stratigraphy: The OxCal program. Radiocarbon 1995; 37:425–430.

[137] Steier P, Rom W. The use of Bayesian statistics for C-14 dates of chronologically ordered samples: A critical analysis. Radiocarbon 2000; 42:183–198.

[138] Bronk Ramsey C. Dealing with outliers and offsets in radiocarbon dating. Radiocarbon 2009; 51:1023–1045.

[139] Bronk Ramsey C, van der Plicht J, Weninger B. ‘Wiggle matching’ radiocarbon dates. Radiocarbon 2001; 43:381–389.

[140] Galimberti M, Bronk Ramsey C, Manning SW. Wiggle-match dating of tree ring sequences. Radiocarbon 2004; 46:917–924.

[141] Sarpaki A. A palaeoethnobotanical study of the West House, Akrotiri, Thera. Annual of the British School at Athens 1992; 87:219–230.

[142] Van Oyen A. The socio-economics of Roman storage: agriculture, trade, and family. Cambridge: Cambridge University Press; 2020.

[143] Halstead P. Texts, bones and herders: approaches to animal husbandry in Late Bronze Age Greece. In Bennet J, Driessen J, editors, A-na-qo-ta. Studies Presented to J. T. Killen. Minos 1998–1999; 33–34:149–189.

[144] Bronk Ramsey C, Manning SW, Galimberti M. Dating the volcanic eruption at Thera. Radiocarbon 2004; 46:325–344.

[145] Cherubini P et al. Olive tree-ring problematic dating: a comparative analysis on Santorini (Greece). PLoS ONE 2013; 8:e54730. doi: 10.1371/journal.pone.0054730 23382949 PMC3557290

[146] Friedrich W, Kromer B, Friedrich M, Heinemeier J, Pfeiffer T, Talamo S. The olive branch chronology stands irrespective of tree-ring counting. Antiquity 2014; 88:274–277. https://doi:10.1017/S0003598X00050377

[147] Ehrlich Y, Regev L, Boaretto E. Discovery of annual growth in a modern olive branch based on carbon isotopes and implications for the Bronze Age volcanic eruption of Santorini. Scientific Reports 2021; 11:704. doi: 10.1038/s41598-020-79024-4 33436660 PMC7804959

[148] Ehrlich Y, Regev L, Boaretto E. Radiocarbon analysis of modern olive wood raises doubts concerning a crucial piece of evidence in dating the Santorini eruption. Scientific Reports 2018; 8:11841 (2018). doi: 10.1038/s41598-018-29392-9 30093696 PMC6085306

[149] Doumas C. The wall-paintings of Thera. London: The Thera Foundation; 1992.

[150] Cole-Dai J et al. Comprehensive record of volcanic eruptions in the Holocene (11,000 years) from the WAIS Divide, Antarctica ice core. Journal of Geophysical Research: Atmospheres 2021; 126:e2020JD032855. 10.1029/2020JD032855

[151] Michaud V, Clocchiatti R, Sbrana S. The Minoan and post-Minoan eruptions, Santorini (Greece), in the light of melt inclusions: chlorine and sulphur behaviour. Journal of Volcanology and Geothermal Research 2000; 99:195–214.

[152] Cadoux A, Scaillet B, Bekki S, Oppenheimer C, Druitt TH. Stratospheric Ozone destruction by the Bronze-Age Minoan eruption (Santorini Volcano, Greece). Scientific Reports 2015; 5:12243. doi: 10.1038/srep12243 26206616 PMC4513290

[153] Sigurdsson H, Carey S, Devine JD. Assessment of Mass, Dynamics and Environmental Effects of the Minoan Eruption of Santorini Volcano. In Hardy DA, Keller J, Galanopoulos VP, Flemming NC, Druitt TH, editors, Thera and the Aegean world III. Volume two: earth sciences. London: The Thera Foundation; 1990. pp.100–112.

[154] Pyle DM. New Estimates for the Volume of the Minoan Eruption. In Hardy DA, Keller J, Galanopoulos VP, Flemming NC, Druitt TH, editors, Thera and the Aegean world III. Volume two: earth sciences. London: The Thera Foundation; 1990. pp.113–121.

[155] Druitt TH et al. Magma Storage and Extraction Associated with Plinian and Interplinian Activity at Santorini Caldera (Greece). Journal of Petrology 2016; 57:461–494.

[156] LaMarche V, Hirschboeck K. Frost rings in trees as records of major volcanic eruptions. Nature 1984; 307:121–126.

[157] Hammer C, Clausen H, Friedrich WL, Tauber H. The Minoan eruption of Santorini in Greece dated to 1645 BC? Nature 1987; 328:517–519.

[158] Baillie M, Munro M. Irish tree rings, Santorini and volcanic dust veils. Nature 1988; 332:344–346.

[159] Pyle DM. The application of tree-ring and ice-core studies to the dating of the Minoan eruption. In Hardy DA, Renfrew AC, editors, Thera and the Aegean world III. Volume three: chronology. London: The Thera Foundation; 1990. pp.167–173.

[160] Pyle DM. On the “Climatic Effectiveness” of Volcanic Eruptions. Quaternary Research 1992; 37:125–129.

[161] Badertscher S et al. Speleothems as sensitive recorders of volcanic eruptions–the Bronze Age Minoan eruption recorded in a stalagmite from Turkey. Earth and Planetary Science Letters 2014; 392:58–66.

[162] Warren PM. Minoan pottery from Egyptian sites. Classical Review 1985; 35:147–151.

[163] Warren PM. Absolute Dating of the Aegean Late Bronze Age. Archaeometry 1987; 29:205–211.

[164] Egypt Muhly J., the Aegean, and Late Bronze Age chronology in the Eastern Mediterranean: a review article. Journal of Mediterranean Archaeology 1991; 4:235–247.

[165] Wiener MH. Chronology going forward (with a query about 1525/4 B.C.). In Czerny E, Hein I, Hunger H, Melman D, Schwab A, editors, Timelines: Studies in Honour of Manfred Bietak. Volume 3. Leuven: Uitgeverij Peeters en Departement Oosterse Studies; 2006. pp.317–328.

[166] Kemp BJ, Merrillees RS. Minoan Pottery in Second Millennium Egypt. Mainz-am-Rhein: Philipp von Zabern; 1980.

[167] Manning SW. Clarifying the ‘High’ v. ‘Low’ Aegean/Cypriot chronology for the mid second millennium BC: assessing the evidence, interpretative frameworks, and current state of the debate. In Bietak M, Czerny E, editors, The Synchronisation of Civilisations in the Eastern Mediterranean in the Second Millennium B.C. III. Proceedings of the SCIEM 2000 – 2nd EuroConference, Vienna 28th of May– 1st of June 2003. Vienna: Verlag der Österreichischen Akademie der Wissenschaften; 2007. pp.101-137.

[168] Cadogan G. Dating the Aegean Bronze Age without radiocarbon. Archaeometry 1978; 20:209–214.

[169] Hallager E. Final palatial Crete. An essay in Minoan chronology. In Damsgaard-Madsen A, Christiansen E, Hallager E, editors, Studies in ancient history and numismatics presented to Rudi Thomsen. Aarhus: Aarhus University Press; 1988.

[170] Höflmayer F. An early date for Khyan and its implications for Eastern Mediterranean chronologies. In Forstner-Müller I, Moeller N, editors, The Hyksos Ruler Khyan and the Early Second Intermediate Period in Egypt: Problems and Priorities of Current Research: Proceedings of the Workshop of the Austrian Archaeological Institute and the Oriental Institute of the University of Chicago, Vienna, July 4–5, 2014. Vienna: Österreichisches Archäologisches Institut/Verlag Holzhausen GmbH; 2018. pp.143-171.

[171] Höflmayer F, Manning SW. A synchronized early Middle Bronze Age chronology for Egypt, the Levant, and Mesopotamia. Journal of Near Eastern Studies 2022; 81:1–24.

[172] Manning SW. Events, Episodes and History: Chronology and the Resolution of Historical Processes. In Nevett L, Whitley J, editors, An Age of Experiment: Classical Archaeology Transformed (1976–2014). Cambridge: McDonald Institute for Archaeological Research; 2018. pp.119–137.

[173] Forstner-Müller I, Moeller N, editors. The Hyksos Ruler Khyan and the Early Second Intermediate Period in Egypt: Problems and Priorities of Current Research: Proceedings of the Workshop of the Austrian Archaeological Institute and the Oriental Institute of the University of Chicago, Vienna, July 4–5, 2014. Vienna: Österreichisches Archäologisches Institut/Verlag Holzhausen GmbH; 2018.

[174] Gillispie CC, Gratton-Guinness I, Fox R. Pierre-Simon Laplace, 1749–1827: A Life in Exact Science. Princeton, NJ: Princeton University Press; 1999.

[175] Doumas CG. Aegeans in the Levant: Myth and Reality. In Gitin S, Mazar A, Stern E, editors, Mediterranean Peoples in Transition: Thirteenth to Early Tenth Centuries BCE. In Honor of Professor Trude Dothan. Jerusalem: Israel Exploration Society; 1998. pp.129–137.

[176] Laffineur R. Material and craftmanship in the Mycenae Shaft Graves: imports vs local production. Minos 1990–1991; 24–25:245–295.

[177] Åström P. Three Tell el Yahudiyeh juglets in the Thera Museum. In Aloyeropoulou AK, editor, Acta of the First International Scientific Congress on the Volcano of Thera, Held in Greece, 15th-23rd September 1969. Athens: Archaeological Services in Greece, General Direction of Antiquities and Restoration; 1971. pp.415-421.

[178] Goldwasser O. How the Alphabet was Born from Hieroglyphs. Biblical Archaeology Review 2010; 36(2):40–53.

[179] Lemaire A. Alphabetic writing in the Mediterranean world: transmission and appropriation. In Halpern B, Sacks KS, editors, Cultural contact and appropriation in the axial-age Mediterranean world: a periplus. Leiden: Brill; 2017. pp.103–115.

[180] Höflmayer F, Misgav H, Webster L, Streit K. Early alphabetic writing in the ancient Near East: the ‘missing link’ from Tel Lachish. Antiquity 2021; 95:705–719.

[181] Renfrew C. The eruption of Thera and Minoan Crete. In Sheets PD, Grayson DK, editors, Volcanic Activity and Human Ecology. New York: Academic Press; 1979. pp.565–585.

[182] Luce JV. Thera and the devastation of Minoan Crete: a new interpretation of the evidence. American Journal of Archaeology 1976; 80:9–16.

[183] Bolton K. Addendum to J.V. Luce’s Article: ’Thera and the Devastation of Minoan Crete: A New Interpretation of the Evidence’. American Journal of Archaeology 1976; 80:17–18.

[184] Michael HN. 1976. Radiocarbon Dates from Akrotiri on Thera. In Betancourt PP, editor, Aegean Art and Archaeology in the Late Bronze Age, Temple University Aegean Symposium 1. Philadelphia: Department of Art History, Temple University; 1976. pp.7–9.

[185] Warren P. The stone vessels from the Bronze Age settlement at Akrotiri, Thera, Aρχαιoλoγική εφημερίς 1979; 118:82–113.

[186] Pearson C et al. Annual Variation in Atmospheric 14C Between 1700 BC and 1480 BC. Radiocarbon 2020; 62:939–952.

[187] MacGillivray JA. Thera, Hatshepsut, and the Keftiu: crisis and response in Egypt and the Aegean in the mid-second millennium BC. In Warburton DA, editor, Time’s Up! Dating the Minoan eruption of Santorini. Aarhus: Aarhus University Press; 2009. pp.154–170.

[188] Wiener MH. The absolute chronology of Late Helladic III A2 revisited. Annual of the British School at Athens 2003; 98:239–250.

[189] Manning SW, Kearns C, Lorentzen B. Dating the end of the Late Bronze Age with radiocarbon: some observations, concerns, and revisiting the dating of Late Cypriot IIC to IIIA. In Fischer PM, Bürge T, editors, “Sea Peoples” Up-to-Date. New Research on Transformation in the Eastern Mediterranean in the 13th-11th Centuries BCE.Vienna: Österreichischen Akademie der Wissenschaften; 2017. pp.95-110.

[190] Manning SW. Time, Consilience and Climate-History Associations: Details, and the Case of the End of the Late Bronze Age (ca. 1200 BC). In Manning SW, editor, Critical Approaches to Cypriot and Wider Mediterranean Archaeology. Sheffield: Equinox; 2022.

[191] Manning SW et al. Integrated tree-ring-radiocarbon high-resolution timeframe to resolve earlier second millennium BCE Mesopotamian chronology. PLoS ONE 2016; 11:e0157144. doi: 10.1371/journal.pone.0157144 27409585 PMC4943651

[192] Manning SW et al. Radiocarbon re-dating of contact-era Iroquoian history in northeastern North America. Science Advances 2018; 4:eaav0280. doi: 10.1126/sciadv.aav0280 30525108 PMC6281431

[193] Manning SW, Birch J, Conger MA, Dee MW, Griggs C, Hadden CS. Contact-Era Chronology Building in Iroquoia: Age Estimates for Arendarhonon Sites and Implications for Identifying Champlian’s Cahiagué. American Antiquity 2019; 84:684–707.

[194] Birch J, Manning SW, Sanft S, Conger MA. Refined radiocarbon chronologies for Northern Iroquoian site sequences: implications for coalescence, conflict, and the reception of European goods. American Antiquity 2021; 86:61–89.

[195] Manning SW, Lorentzen B, Hart JP. 2021. Resolving Indigenous village occupations and social history across the long century of European permanent settlement in Northeastern North America: The Mohawk River Valley ~1450–1635 CE. PLoS ONE 2021; 16:e0258555. doi: 10.1371/journal.pone.0258555 34653214 PMC8519479

[196] Lolos YG. On the Late Helladic I of Akrotiri, Thera. In Hardy DA, Renfrew AC, editors, Thera and the Aegean world III. Volume three: chronology. London: The Thera Foundation; 1990. pp.51–56.

[197] Wiener MH. Problems in the Measurement, Calibration, Analysis, and Communication of Radiocarbon Dates (With Special Reference to the Prehistory of the Aegean World). Radiocarbon 2012; 54:423–434.

[198] Merrillees RS. Chronological conundrums: Cypriot and Levantine imports from Thera. In Warburton DA, editor, Time’s Up! Dating the Minoan eruption of Santorini. Aarhus: Aarhus University Press; 2009. pp.247–251.

[199] Dietz S. The Argolid at the transition to the Mycenaean age: studies in the chronology and cultural development in the shaft grave period. Copenhagen: National Museum of Denmark; 1991.

[200] Bietak M. Relative and Absolute Chronology of the Middle Bronze Age: Comments on the Present State of Research. In Bietak M, editor, The Middle Bronze Age in the Levant: Proceedings of an International Conference on MB IIA Ceramic Material. Contributions to the Chronology of the Eastern Mediterranean 3. Vienna: Verlag der Österreichischen Akademie der Wissenschaften; 2002. pp.29-42.

[201] Kutschera W et al. The chronology of Tell el-Daba: a crucial meeting oint of 14C dating, archaeology, and Egyptology in the 2nd millennium BC. Radiocarbon 2012; 54:407–422.

[202] Knappett C, Rivers R, Evans T. The Theran eruption and Minoan palatial collapse: new interpretations gained from modelling the maritime network. Antiquity 2011; 85:1008–1023.

[203] Momigliano N. Bronze Age Carian Iasos: Structures and Finds from the Area of the Roman Agora (c. 3000–1500 BC). Rome: Giorgio Bretschneider Editore; 2012.

[204] Bottema S, Sarpaki A. Environmental change in Crete: a 9000-year record of Holocene vegetation history and the effect of the Santorini eruption. The Holocene 2003; 13:733–749.

[205] Eastwood WJ, Tibby J, Roberts N, Birks HJB, Lamb HF. The environmental impact of the Minoan eruption of Santorini (Thera): statistical analysis of palaeoecological data from Gölhisar, southwest Turkey. The Holocene 2002; 12:431–444.

[206] Kulick R, Westgate J. Tephrochronology and micromorphology of Theran tephra deposits at Palaikastro, Crete. Journal of Archaeological Science: Reports 2021; 36:102884. 10.1016/j.jasrep.2021.102884

[207] Mountjoy P.A. The Marine Style pottery of LMIB/LHIIA: towards a corpus. Annual of the British School at Athens 1984; 79:161–219.

[208] Bicknell P. Late Minoan IB marine ware, the marine environment of the Aegean, and the Bronze Age eruption of the Thera volcano. In McGuire WJ, Griffiths DR, Hancock PL, Stewart IS, editors, The Archaeology of Geological Catastrophes. Special Publications 171. London: Geological Society; 2000. pp. 95–103.

