# Supplementary material for: Second Intermediate Period date for the Thera (Santorini) eruption and historical implications
Source: PLoS One. 2022 Sep 20;17(9):e0274835. doi: 10.1371/journal.pone.0274835 (PMC9488803; doi:10.1371/journal.pone.0274835)
Supplement: S1 Table — (DOCX) [file pone.0274835.s001.docx]

Table S1. The ^14^C dates employed in this study in four parts: Table S1A, Table S1B, Table S1C and Table S1D.

Table S1A. Samples and ^14^C dates employed in this paper relevant to the Thera date. These include samples from Thera from the final volcanic destruction level contexts (VDL, LCI Advanced) and samples from other Aegean contexts remote from Thera which are directly related to the Thera eruption (by Theran airfall tephra deposits) by or associated tsunami. The dates included from Thera are those published on samples stated as from the volcanic destruction level (VDL) on Thera/Santorini – thus final VDL phase or LCI Advanced phase at Akrotiri where stated or clarified, or from likely immediate pre-eruption contexts elsewhere from Thera/Santorini. Exceptions not included are samples that were stated as under-sized for the given technology used (P-1599, P-1619, P-2562, P-2563, P-2564, P-2566), and two dates that, for whatever reason, are much too old/deviants (P-2561, P-2560). Where dates were published in *Radiocarbon* date-lists those (rounded) values were used (P-1697, P-1885, P-1888, P-1889, P-1892, P-1894, P-1895). Dates that were never published (the ^14^C measurements not provided) are not included [94]. The focus is on short/shorter-lived samples without any in-built age factor. Dates on wood-charcoal from Akrotiri, Thera, or Trianda, Rhodes, unless stated to be from very final VDL (LCI Advanced) contexts or LMIA late and immediately pre-eruption tephra layer contexts, are not used and are assumed to represent older ages. This includes the set of ETH dates on charcoal listed in [88] which in several cases appear rather too old and perhaps relate to earlier Middle Bronze Age wood use in some cases. Dates on charcoal samples from LMIA late contexts that clearly represent samples with in-built age, e.g. ^14^C ages >3450 ^14^C years BP with measurement error <50 (whether older tree-rings or wood cut and first used in an earlier period like late MBA) were also excluded (e.g. OxA-10641 from LMIA late Trianda, Rhodes). Dates from samples processed to potentially remove a suspected older contaminant and published as ‘contaminant’ (versus ‘residue’) [68] were not used. The ‘residue’ dates are included. Dates on shell samples with a marine reservoir component (GrA-21607) were not used. All references to ‘pumice’ or ‘tephra’ below refer to what is identified as Minoan Thera/Santorini eruption pumice or tephra.

| **No.** | **Date ID** | **Site** | **Archaeological Context** | **Material** | **^14^C Date**  **yrs BP** | **SD** | **Reference** |
| --- | --- | --- | --- | --- | --- | --- | --- |
| 1 | DEM-94 | Trianda, Rhodes | LBIA/LMIA mature/destruction below Thera tephra | charcoal | 3347 | 46 | 73 |
| 2 | DEM-93 | Trianda, Rhodes | LBIA/LMIA mature/destruction below Thera tephra | charcoal | 3358 | 48 | 73 |
| 3 | OxA-10643 | Trianda, Rhodes | LMIA late below Thera tephra | *Quercus* sp. twig | 3367 | 39 | 64 |
| 4 | OxA-11884 | Trianda, Rhodes | LMIA late below Thera tephra | *Quercus* sp. twig | 3344 | 32 | 64 |
| 5 | GrA-30336 | Palaikastro promontory, Crete | Thera tsunami deposit | cattle bone | 3310 | 35 | 52,74 |
| 6 | GrA-30339 | Palaikastro promontory, Crete | Thera tsunami deposit | cattle bone | 3390 | 35 | 52,74 |
| 7 | GrA-28991 | Palaikastro, Crete | Drain deposit with stratified Thera tephra | Goat/sheep bone/jaw | 3325 | 40 | 74 |
| 8 | GrA-29041 | Palaikastro, Crete | Drain deposit with stratified Thera tephra | Goat/sheep bone/jaw | 3345 | 40 | 74 |
| 9 | GrA-29042 | Palaikastro, Crete | Drain deposit with stratified Thera tephra | Tooth animal | 3385 | 40 | 74 |
| 10 | OxA-38858 | Çeşme-Bağlararası, Turkey | Thera tsunami deposit | *Hordeum vulgare* | 3275 | 17 | 45 |
| 11 | OxA-38881 | Çeşme-Bağlararası, Turkey | Thera tsunami deposit | Bos taurus | 3367 | 22 | 45 |
| 12 | OxA-38973 | Çeşme-Bağlararası, Turkey | Thera tsunami deposit | Ovis aries | 3318 | 19 | 45 |
| 13 | OxA-38972 | Çeşme-Bağlararası, Turkey | Thera tsunami deposit | Sus scrofa | 3316 | 20 | 45 |
| 14 | OxA-38857 | Çeşme-Bağlararası, Turkey | Thera tsunami deposit | *Olea europaea* | 3312 | 17 | 45 |
| 15 | OxA-38950 | Çeşme-Bağlararası, Turkey | Thera tsunami deposit | charcoal | 3384 | 22 | 45 |
| 16 | D-AMS 019172 | Çeşme-Bağlararası, Turkey | Thera tsunami deposit | bone (unidentified) | 3372 | 27 | 45 |
| 17 | OxA-38966 | Çeşme-Bağlararası, Turkey | Thera tsunami deposit | charcoal | 3297 | 19 | 45 |
| 18 | D-AMS 019173 | Çeşme-Bağlararası, Turkey | Thera tsunami deposit | charcoal | 3291 | 30 | 45 |
| 19 | Lyon7920 | Letoon Sanctuary, Eşençay Delta | Organic layer associated/immediately below Thera tephra | Organic-rich debris layer | 3295 | 30 | 54 |
| 20 | OxA-12301 | Miletos, Turkey | LMIA below Thera tephra | *Quercus* sp. Relative Years (RY)1000-1010 | 3439 | 30 | 64 |
| 21 | OxA-12302 | Miletos, Turkey | LMIA below Thera tephra | *Quercus* sp. RY1000-1010 | 3386 | 31 | 64 |
| 22 | OxA-12303 | Miletos, Turkey | LMIA below Thera tephra | *Quercus* sp. RY1010-1020 | 3467 | 31 | 64 |
| 23 | OxA-12407 | Miletos, Turkey | LMIA below Thera tephra | *Quercus* sp. RY1010-1020 | 3385 | 34 | 64 |
| 24 | OxA-12304 | Miletos, Turkey | LMIA below Thera tephra | *Quercus* sp. RY1020-1030 | 3404 | 31 | 64 |
| 25 | OxA-12305 | Miletos, Turkey | LMIA below Thera tephra | *Quercus* sp. RY1020-1030 | 3459 | 31 | 64 |
| 26 | OxA-12306 | Miletos, Turkey | LMIA below Thera tephra | *Quercus* sp. RY1030-1040 | 3416 | 31 | 64 |
| 27 | OxA-12307 | Miletos, Turkey | LMIA below Thera tephra | *Quercus* sp. RY1030-1040 | 3425 | 31 | 64 |
| 28 | OxA-12308 | Miletos, Turkey | LMIA below Thera tephra | *Quercus* sp. RY1040-1050 | 3361 | 31 | 64 |
| 29 | OxA-12309 | Miletos, Turkey | LMIA below Thera tephra | *Quercus* sp. RY1040-1050 | 3397 | 32 | 64 |
| 30 | OxA-12310 | Miletos, Turkey | LMIA below Thera tephra | *Quercus* sp. RY1050-1060 | 3345 | 32 | 64 |
| 31 | OxA-12311 | Miletos, Turkey | LMIA below Thera tephra | *Quercus* sp. RY1050-1060 | 3397 | 32 | 64 |
| 32 | OxA-12312 | Miletos, Turkey | LMIA below Thera tephra | *Quercus* sp. RY1060-1070 | 3388 | 30 | 64 |
| 33 | OxA-12313 | Miletos, Turkey | LMIA below Thera tephra | *Quercus* sp. RY1060-1070  (waney edge RY1072) | 3352 | 31 | 64 |
| 34 | DEM-1311 | Akrotiri, Thera | M2/1N002 LCI VDL | charcoal | 3307 | 24 | 78 |
| 35 | DEM-1529 | Akrotiri, Thera | M44A/26V LCI Advanced? VDL | charcoal | 3281 | 25 | 78 |
| 36 | DEM-1607 | Akrotiri, Thera | M26/43N047 LCI Advanced VDL | charcoal | 3228 | 30 | 78 |
| 37 | DEM-1624 | Akrotiri, Thera | 14/1BN005 LCI Advanced VDL | wood | 3360 | 25 | 78 |
| 38 | DEM-1615 | Akrotiri, Thera | M2/53AN003 LCI Advanced? | charcoal | 3389 | 25 | 78 |
| 39 | OxA-1552 | Akrotiri, Thera | VDL W. House Rm 5 | *Lathyrus cly.* | 3390 | 65 | 68 |
| 40 | OxA-1555 | Akrotiri, Thera | VDL W. House Rm 5 | *Lathyrus cly.* | 3245 | 65 | 68 |
| 41 | OxA-1548 | Akrotiri, Thera | VDL W. House Rm 5 | *Lathyrus cly.* | 3335 | 60 | 68 |
| 42 | OxA-1549 | Akrotiri, Thera | VDL W. House Rm 5 | *Lathyrus cly.* | 3460 | 80 | 68 |
| 43 | OxA-1550 | Akrotiri, Thera | VDL W. House Rm 5 | *Lathyrus* sp. | 3395 | 65 | 68 |
| 44 | OxA-1553 | Akrotiri, Thera | VDL W. House Rm 5 | *Lathyrus cly.* | 3340 | 65 | 68 |
| 45 | OxA-1554 | Akrotiri, Thera | VDL W. House Rm 5 | *Lathyrus cly.* | 3280 | 65 | 68 |
| 46 | OxA-1556 | Akrotiri, Thera | VDL W. House Rm 5 | *Hordeum* sp*.* | 3415 | 70 | 68 |
| 47 | Hd-7092-6795 | Akrotiri, Thera | VDL W. House Rm 5 | peas | 3360 | 60 | 77 |
| 48 | K-5352 | Akrotiri, Thera | VDL W. House Rm 3 | pulses | 3310 | 65 | 76 |
| 49 | K-3228 | Akrotiri, Thera | VDL W. House Rm 5 | pulses | 3340 | 55 | 76 |
| 50 | K-4255 | Akrotiri, Thera | VDL House 3, delta 1 | *Tamarix* twig 10 growth rings | 3380 | 60 | 76 |
| 51 | OxA-11817 | Akrotiri, Thera | VDL M2/76 N003 | ? *Lathyrus* sp. | 3348 | 31 | 64 |
| 52 | OxA-11818 | Akrotiri, Thera | VDL M7/68A N004 | *Hordeum* sp. | 3367 | 33 | 64 |
| 53 | OxA-11820 | Akrotiri, Thera | VDL M10/23A N012 | *Hordeum* sp. | 3400 | 31 | 64 |
| 54 | OxA-11869 | Akrotiri, Thera | VDL M31/43 N047 | *Hordeum* sp. | 3336 | 34 | 64 |
| 55 | OxA-12170 | Akrotiri, Thera | VDL M2/76 N003 | ? *Lathyrus* sp. | 3336 | 28 | 64 |
| 56 | OxA-12171 | Akrotiri, Thera | VDL M7/68A N004 | *Hordeum* sp. | 3372 | 28 | 64 |
| 57 | OxA-12172 | Akrotiri, Thera | VDL M31/43 N047 | *Hordeum* sp. | 3321 | 32 | 64 |
| 58 | OxA-12175 | Akrotiri, Thera | VDL M10/23A N012 | *Hordeum* sp. | 3318 | 28 | 64 |
| 59 | VERA-2756 | Akrotiri, Thera | VDL M31/43 N047 | *Hordeum* sp. | 3317 | 28 | 64 |
| 60 | VERA-2757 | Akrotiri, Thera | VDL M2/76 N003 | ? *Lathyrus* sp. | 3315 | 31 | 64 |
| 61 | VERA-2758 | Akrotiri, Thera | VDL M7/68A N004 | *Hordeum* sp. | 3339 | 28 | 64 |
| 62 | VERA-2757repeat | Akrotiri, Thera | VDL M2/76 N003 | ? *Lathyrus* sp. | 3390 | 32 | 64 |
| 63 | VERA-2758repeat | Akrotiri, Thera | VDL M7/68A N004 | *Hordeum* sp. | 3322 | 32 | 64 |
| 64 | OxA-25176 | Akrotiri, Thera | VDL W. House Rm 5 | insect chitin | 3368 | 29 | 79 |
| 65 | Hd-23599-24426 | Thera | Eruption pumice | Olive branch inner segment ‘rings’ 1-13 | 3383 | 11 | 80 |
| 66 | Hd-23587 | Thera | Eruption pumice | Olive branch next segment ‘rings’ 14-37 | 3372 | 12 | 80 |
| 67 | Hd-23589 | Thera | Eruption pumice | Olive branch next segment ‘rings’ 38-59 | 3349 | 12 | 80 |
| 68 | Hd-23588-24402 | Thera | Eruption pumice | Olive branch outer segment ‘rings’ 60-72 | 3331 | 10 | 80 |
| 69 | VERA-5614 | Thera | Pumice/pre-eruption | Olive branch ABA | 3282 | 21 | 82 |
| 70 | VERA-5614HS | Thera | Pumice/pre-eruption | Olive branch HS (extracted humic acids) | 3359 | 33 | 82 |
| 71 | VERA-5615 | Thera | Pumice/pre-eruption | Olive branch ABA | 3280 | 24 | 82 |
| 72 | VERA-5615HS | Thera | Pumice/pre-eruption | Olive branch HS | 3321 | 24 | 82 |
| 73 | VERA-5620 | Thera | Pumice/pre-eruption | Olive branch ABA | 3277 | 25 | 82 |
| 74 | VERA-5620HS | Thera | Pumice/pre-eruption | Olive branch HS | 3345 | 24 | 82 |
| 75 | VERA-5610 | Thera | Pumice/pre-eruption | Olive branch ABA | 3399 | 25 | 82 |
| 76 | VERA-5610HS | Thera | Pumice/pre-eruption | Olive branch HS | 3342 | 26 | 82 |
| 77 | VERA-5083 | Thera | Pumice/pre-eruption | Olive branch ABA | 3270 | 36 | 82 |
| 78 | VERA-5083HS | Thera | Pumice/pre-eruption | Olive branch HS | 3326 | 77 | 82 |
| 79 | VERA-5082 | Thera | Pumice/pre-eruption | Olive branch ABA | 3332 | 38 | 82 |
| 80 | VERA-5082HS | Thera | Pumice/pre-eruption | Olive branch HS | 3369 | 36 | 82 |
| 81 | VERA-5084 | Thera | Pumice/pre-eruption | Olive root ABA | 3354 | 32 | 82 |
| 82 | VERA-5084HS | Thera | Pumice/pre-eruption | Olive root HS | 3368 | 34 | 81 |
| 83 | K-3227 | Athinios Quarry, Thera | Under pumice | charcoal | 3400 | 70 | 75 |
| 84 | K-5353 | Akrotiri, Thera | VDL Bronos 1a hearth | pulses | 3430 | 90 | 76 |
| 85 | P-2794 | Akrotiri, Thera | VDL W. House Rm 5 | Soil? from jar | 3180 | 50 | 87 |
| 86 | P-1890 | Akrotiri, Thera | LMIA structure B Rm 5 | Charcoal (pine) | 3340 | 60 | 86 |
| 87 | L-362 | Phira Quarry | Below pumice | Charred wood | 3370 | 100 | 83 |
| 88 | P-1401 | Phira Quarry | Below pumice | Carbonized tree | 3420 | 40 | 85 |
| 89 | P-1697 | Boudouroglou mine | Below pumice | Carbonized beans | 3070 | 60 | 85 |
| 90 | P-1885 | Akrotiri, Thera | Structure D | Charred seeds  (legumes?) | 3250 | 50 | 85 |
| 91 | P-1888 | Akrotiri, Thera | Structure D | Charcoal, shrubs? | 3130 | 50 | 85 |
| 92 | P-1889 | Akrotiri, Thera | Structure D | Charcoal, shrubs? | 3300 | 50 | 85 |
| 93 | P-1892 | Akrotiri, Thera | Bronos Bridge Area 6 | Charcoal, shrubs? | 3330 | 50 | 85 |
| 94 | P-1894 | Akrotiri, Thera | Structure Delta Rm 3 | Charcoal, shrubs? | 3310 | 70 | 85 |
| 95 | P-1895 | Akrotiri, Thera | Structure Beta | Charcoal, shrubs? | 3320 | 50 | 85 |
| 96 | P-2559 | Akrotiri, Thera | VDL W. House Rm 5 | grains | 3370 | 70 | 86 |
| 97 | P-2565 | Akrotiri, Thera | VDL W. House Rm 5 | grains | 3310 | 60 | 86 |
| 98 | P-2791 | Akrotiri, Thera | VDL W. House Rm 5 | Seeds and charred material | 3340 | 60 | 87 |
| 99 | Hd-6058-5519 | Akrotiri, Thera | VDL Akrotiri 6 | grains | 3490 | 80 | 77 |
| 100 | Hd-6059-7967 | Akrotiri, Thera | VDL Akrotiri 6 | grains | 3140 | 70 | 77 |
| 101 | ETH-3315 | Akrotiri, Thera | VDL | seed | 3610 | 51 | 88 |
| 102 | ETH-3323 | Akrotiri, Thera | VDL | seed | 3437 | 54 | 88 |
| 103 | ETH-3324 | Akrotiri, Thera | VDL | seed | 3453 | 52 | 88 |
| 104 | OxA-1558 | Akrotiri, Thera | VDL stages (i)/(ii) | legumes | 3400 | 60 | 68 |
| 105 | OxA-1551 | Akrotiri, Thera | VDL stages (i)/(ii) | *Hordeum* sp. | 3465 | 65 | 68 |
| 106 | OxA-1557 | Akrotiri, Thera | VDL stages (i)/(ii) | legumes | 3240 | 60 | 68 |
| 107 | OxA-1687 | Akrotiri, Thera | VDL W. House Rm 5 | *Lathyrus* sp.  ‘residue’ fraction | 3280 | 60 | 68 |
| 108 | OxA-1689 | Akrotiri, Thera | VDL W. House Rm 5 | *Lathyrus* sp.  ‘residue’ fraction | 3270 | 60 | 68 |
| 109 | OxA-1691 | Akrotiri, Thera | VDL W. House Rm 5 | *Lathyrus* sp.  ‘residue’ fraction | 3320 | 60 | 68 |
| 110 | OxA-1692 | Akrotiri, Thera | VDL W. House Rm 5 | Legumes ‘residue’ fraction | 3325 | 90 | 68 |

Table S1B. Samples and ^14^C dates employed in this paper offering indications for the start of LHI (dataset i – from the modelled Phase J/K transition), early LMIA (dataset j) for mid-LHI and late-LHI (dataset k-1, k-2), and for LHII (Thera eruption TAQ, dataset i – VERA-4630).

| **No.** | **Date ID** | **Site** | **Archaeological Context** | **Material** | **^14^C Date**  **yrs BP** | **SD** | **Reference** |
| --- | --- | --- | --- | --- | --- | --- | --- |
| 111 | VERA-2678 | Kolonna, Aegina | Ceramic Phase E | *Hordeum vulgare* | 3724 | 35 | 89 |
| 112 | VERA-2680 | Kolonna, Aegina | Ceramic Phase E | *Hordeum vulgare* | 3722 | 35 | 89 |
| 113 | VERA-2681 | Kolonna, Aegina | Ceramic Phase E | *Hordeum vulgare* | 3739 | 35 | 89 |
| 114 | VERA-2679 | Kolonna, Aegina | Ceramic Phase E | *Hordeum vulgare* | 3761 | 35 | 89 |
| 115 | VRI-0395 | Kolonna, Aegina | Ceramic Phase E | charcoal | 3670 | 90 | 89 |
| 116 | HV-5841 | Kolonna, Aegina | Ceramic Phase E | charcoal | 3625 | 65 | 89 |
| 117 | VERA-2682 | Kolonna, Aegina | Ceramic Phase E | *Hordeum vulgare* | 3712 | 35 | 89 |
| 118 | VERA-2683 | Kolonna, Aegina | Ceramic Phase E | *Hordeum vulgare* | 3721 | 35 | 89 |
| 119 | HV-5840 | Kolonna, Aegina | Ceramic Phase E | charcoal | 3820 | 60 | 89 |
| 120 | VERA-4641 | Kolonna, Aegina | Ceramic Phase E | Bone *Ovis/Capra* | 3759 | 35 | 89 |
| 121 | VERA-2688 | Kolonna, Aegina | Ceramic Phase E | Bone *Ovis/Capra* | 3698 | 33 | 89 |
| 122 | VERA-2692 | Kolonna, Aegina | Ceramic Phase F | Bone (goat) | 3704 | 36 | 89 |
| 123 | VERA-4640 | Kolonna, Aegina | Ceramic Phase G | Bone *Bos* | 3800 | 44 | 89 |
| 124 | VERA-4639 | Kolonna, Aegina | Ceramic Phase G | Bone *Bos* | 3809 | 32 | 89 |
| 125 | VERA-4638 | Kolonna, Aegina | Ceramic Phase G | Bone *Ovis/Capra* | 3646 | 32 | 89 |
| 126 | VERA-4281 | Kolonna, Aegina | Ceramic Phase G | *Hordeum vulgare* | 3740 | 36 | 89 |
| 127 | VERA-4282 | Kolonna, Aegina | Ceramic Phase G | *Hordeum vulgare* | 3711 | 34 | 89 |
| 128 | VERA-4283 | Kolonna, Aegina | Ceramic Phase G | *Hordeum vulgare* | 3780 | 37 | 89 |
| 129 | VERA-4637 | Kolonna, Aegina | Ceramic Phase H | Bone *Bos* | 3643 | 30 | 89 |
| 130 | VERA-4636 | Kolonna, Aegina | Ceramic Phase H | Bone *Ovis/Capra* | 3628 | 30 | 89 |
| 131 | VERA-4280 | Kolonna, Aegina | Ceramic Phase H | *Hordeum vulgare* | 3724 | 39 | 89 |
| 132 | VERA-4279 | Kolonna, Aegina | Ceramic Phase H | *Hordeum vulgare* | 3718 | 38 | 89 |
| 133 | VERA-2687 | Kolonna, Aegina | Ceramic Phase H | Bone *Bos* | 3694 | 35 | 89 |
| 134 | VERA-4634 | Kolonna, Aegina | Ceramic Phase I | Bone *Sus* | 3544 | 37 | 89 |
| 135 | VERA-4278 | Kolonna, Aegina | Ceramic Phase I | Cereal indeterminate | 3522 | 38 | 89 |
| 136 | VERA-4038 | Kolonna, Aegina | Ceramic Phase I | Cereal indeterminate | 3506 | 34 | 89 |
| 137 | VERA-4576 | Kolonna, Aegina | Ceramic Phase I | Bone *Bos* | 3482 | 37 | 89 |
| 138 | VERA-4575 | Kolonna, Aegina | Ceramic Phase I | Bone *Sus* | 3537 | 36 | 89 |
| 139 | VERA-4578 | Kolonna, Aegina | Ceramic Phase I | Bone *Ovis/Capra* | 3501 | 39 | 89 |
| 140 | VERA-4579 | Kolonna, Aegina | Ceramic Phase I | Bone *Ovis/Capra* | 3526 | 38 | 89 |
| 141 | VERA-4580 | Kolonna, Aegina | Ceramic Phase I | Bone *Bos* | 3506 | 33 | 89 |
| 142 | VERA-4276 | Kolonna, Aegina | Ceramic Phase I | Cereal indeterminate | 3506 | 37 | 89 |
| 143 | VERA-4275 | Kolonna, Aegina | Ceramic Phase I | Cereal indeterminate | 3544 | 38 | 89 |
| 144 | VERA-4577 | Kolonna, Aegina | Ceramic Phase I | Bone *Ovis/Capra* | 3458 | 39 | 89 |
| 145 | VERA-4571 | Kolonna, Aegina | Ceramic Phase J | Bone *Ovis/Capra* | 3469 | 38 | 89 |
| 146 | VERA-4574 | Kolonna, Aegina | Ceramic Phase J | Bone *Sus* | 3430 | 39 | 89 |
| 147 | VERA-4573 | Kolonna, Aegina | Ceramic Phase J | Bone *Ovis/Capra* | 3485 | 36 | 89 |
| 148 | VERA-4572 | Kolonna, Aegina | Ceramic Phase J | Bone *Ovis/Capra* | 3407 | 38 | 89 |
| 149 | VERA-4570 | Kolonna, Aegina | Ceramic Phase J | Bone *Sus* | 3428 | 36 | 89 |
| 150 | VERA-4633 | Kolonna, Aegina | Ceramic Phase K | Bone *Ovis/Capra* | 3333 | 29 | 89 |
| 151 | VERA-4632 | Kolonna, Aegina | Ceramic Phase K | Bone *Ovis/Capra* | 3356 | 36 | 89 |
| 152 | VERA-4631 | Kolonna, Aegina | Ceramic Phase K | Bone *Ovis/Capra* | 3349 | 36 | 89 |
| 153 | VERA-4630 | Kolonna, Aegina | Ceramic Phase L | Bone *Ovis/Capra* | 3313 | 48 | 89 |
| 154 | VERA-4284 | Kolonna, Aegina | Ceramic Phase M | *Hordeum vulgare* | 3044 | 35 | 89 |
| 155 | VERA-4582 | Kolonna, Aegina | Ceramic Phase M | Charred wood (twig) | 2986 | 33 | 89 |
| 156 | VERA-4285 | Kolonna, Aegina | Ceramic Phase M | legumes | 3040 | 37 | 89 |
| 157 | OxA-11251 | Kommos, Crete | Early LMIA K85A/62D/9:92 | *Quercus* sp. | 3505 | 40 | 64,90 |
| 158 | VERA-2636 | Kommos, Crete | Early LMIA K85A/62D/9:92 | *Quercus* sp. | 3445 | 25 | 64,90 |
| 159 | OxA-11253 | Kommos, Crete | Early LMIA K85A/62D/8:83 | *Quercus* sp. | 3397 | 38 | 64,90 |
| 160 | VERA-2638 | Kommos, Crete | Early LMIA K85A/62D/8:83 | *Quercus* sp. | 3600 | 19 | 64,90 |
| 161 | OxA-11883 | Kommos, Crete | Early LMIA Space 25B Tr.66B | Likely *Cupressaceae* | 3485 | 33 | 64,90 |
| 162 | OxA-11944 | Kommos, Crete | Early LMIA Space 25B Tr.66B | Likely *Cupressaceae* | 3435 | 25 | 64,90 |
| 163 | OxA-3429 | Kommos, Crete | Early LMIA Space 25B Tr.66B | Likely *Cupressaceae* | 3350 | 70 | 64,90 |
| 164 | OxA-11252 | Kommos, Crete | Early LMIA K85A/66B/4:22+23 | Charred twig | 3375 | 45 | 64,90 |
| 165 | VERA-2637 | Kommos, Crete | Early LMIA K85A/66B/4:22+23 | Charred twig | 3390 | 20 | 64,90 |
| 166 | OxA-20127 | Lerna Shaft Grave 1 | Mid-LHI | Animal bone, *Ovis/Capra* metacarpus | 3522 | 27 | 91 |
| 167 | OxA-20128 | Lerna Shaft Grave 1 | Mid-LHI | Animal bone, *Ovis/Capra* metacarpus | 3479 | 27 | 91 |
| 168 | OxA-20170 | Lerna Shaft Grave 1 | Mid-LHI | Animal bone, *Ovis/Capra* R ulna | 3360 | 29 | 91 |
| 169 | OxA-22634 | Lerna Shaft Grave 1 | Mid-LHI | Animal bone, *Cervus* (or *Bos*?) femur | 3314 | 27 | 91 |
| 170 | OxA-22635 | Lerna Shaft Grave 1 | Mid-LHI | Animal bone, *Equus asinus* radius | 3391 | 26 | 91 |
| 171 | OxA—X-2304-54 | Lerna Shaft Grave 1 | Mid-LHI | Animal bone, *Cervus* tibia | 3378 | 28 | 91 |
| 172 | OxA-20126 | Lerna Shaft Grave 2 | Late LHI | Animal bone, *Bos* MT | 3356 | 27 | 91 |
| 173 | OxA-20128 | Lerna Shaft Grave 2 | Late LHI | Animal bone, *Ovis/Capra* metacarpus | 3326 | 28 | 91 |
| 174 | OxA-20169 | Lerna Shaft Grave 2 | Late LHI | Animal bone, *Ovis/Capra* metacarpus | 3393 | 31 | 91 |
| 175 | OxA-22631 | Lerna Shaft Grave 2 | Late LHI | Animal bone, *Ovis/Capra* metacarpus | 3276 | 27 | 91 |
| 176 | OxA-22632 | Lerna Shaft Grave 2 | Late LHI | Animal bone, *Sus* Mc III | 3313 | 27 | 91 |
| 177 | OxA-22633 | Lerna Shaft Grave 2 | Late LHI | Animal bone, *Bos* metacarpus | 3275 | 26 | 91 |

Table S1C. Samples and ^14^C dates employed in this paper relevant to the LMIB destructions at Chania, Myrtos-Pyrgos and Mochlos on Crete and the LMII destruction at Knossos on Crete. Only modern AMS ^14^C ages on short-lived samples are employed in the analyses in this paper from these four sites.

| **No.** | **Date ID** | **Site** | **Archaeological Context** | **Material** | **^14^C Date**  **yrs BP** | **SD** | **Reference** |
| --- | --- | --- | --- | --- | --- | --- | --- |
| 178 | OxA-2517 | Chania, Crete | LMIB Destruction TR10, Rm E | *Pisum sativum* | 3380 | 80 | 64,99 |
| 179 | OxA-2518 | Chania, Crete | LMIB Destruction TR17, 1984, Rm C | *Vicia faba* | 3340 | 80 | 64,99 |
| 180 | OxA-2646 | Chania, Crete | LMIB Destruction TR17, 1984, Rm C | *Hordeum* sp. | 3315 | 70 | 64,99 |
| 181 | OxA-2647 | Chania, Crete | LMIB Destruction TR24, 1989, L6, BA1 | Charred indeterminate seed remains | 3150 | 70 | 64,99 |
| 182 | OxA-10320 | Chania, Crete | LMIB Destruction TR17, 1984, Rm C | *Vicia faba* | 3208 | 26 | 64 |
| 183 | OxA-10321 | Chania, Crete | LMIB Destruction TR17, 1984, Rm C | *Hordeum* sp. | 3268 | 27 | 64 |
| 184 | OxA-10322 | Chania, Crete | LMIB Destruction TR10, Rm E | *Pisum sativum* | 3338 | 26 | 64 |
| 185 | OxA-10323 | Chania, Crete | LMIB Destruction TR24, 1989, L6, BA1 | Charred indeterminate seed remains | 3253 | 25 | 64 |
| 186 | OxA-3187 | Myrtos-Pyrgos, Crete | LMIB Destruction K5,2,1 | *Hordeum* sp. | 3230 | 70 | 64,99 |
| 187 | OxA-3188 | Myrtos-Pyrgos, Crete | LMIB Destruction K5,2,4 | *Hordeum* sp. | 3200 | 70 | 64,99 |
| 188 | OxA-3189 | Myrtos-Pyrgos, Crete | LMIB Destruction K5/K6,2,1 | *Vicia ervilia* | 3270 | 70 | 64,99 |
| 189 | OxA-3225 | Myrtos-Pyrgos, Crete | LMIB Destruction K5/L6,2,2 | *Vicia ervilia* | 3160 | 80 | 64,99 |
| 190 | OxA-10324 | Myrtos-Pyrgos, Crete | LMIB Destruction K5,2,1 | *Hordeum* sp. | 3270 | 26 | 64 |
| 191 | OxA-10325 | Myrtos-Pyrgos, Crete | LMIB Destruction K5/K6,2,1 | *Vicia ervilia* | 3228 | 26 | 64 |
| 192 | OxA-10326 | Myrtos-Pyrgos, Crete | LMIB Destruction K5/L6,2,2 | *Vicia ervilia* | 3227 | 25 | 64 |
| 193 | OxA-10411 | Myrtos-Pyrgos, Crete | LMIB Destruction K5,2,4 | *Hordeum* sp. | 3150 | 40 | 64 |
| 194 | Beta-85991 | Mochlos, Crete | LMIB Destruction B.kiln.2910 | *Olea europaea* stone | 3240 | 50 | 210 |
| 195 | Beta-85992 | Mochlos, Crete | LMIB Destruction A.2.212 | *Olea europaea* stone | 3180 | 40 | 210 |
| 196 | Beta-115890 | Mochlos, Crete | LMIB Destruction B.kiln.2801 | *Olea europaea* stones | 3170 | 60 | 210 |
| 197 | Beta-129765 | Mochlos, Crete | LMIB Destruction B.9.1705 | *Olea europaea* stone | 3220 | 40 | 210 |
| 198 | Beta-151768 | Mochlos, Crete | LMIB Destruction A.pit.2315N | *Olea europaea* stone | 3270 | 40 | 210 |
| 199 | OxA-2096 | Knossos, Crete | LMII Destruction, MUMK | *Hordeum* sp. | 3070 | 70 | 64,68 |
| 200 | OxA-2097 | Knossos, Crete | LMII Destruction, MUMK | *Hordeum* sp. | 3190 | 65 | 64,68 |
| 201 | OxA-2098 | Knossos, Crete | LMII Destruction, MUMK | *Hordeum* sp. | 3220 | 65 | 64,68 |
| 202 | OxA-11882 | Knossos, Crete | LMII Destruction, MUMK | *Hordeum* sp. | 3156 | 33 | 64 |
| 203 | OxA-11943 | Knossos, Crete | LMII Destruction, MUMK | *Hordeum* sp. | 3148 | 23 | 64 |

Table S1D. Additional samples and ^14^C dates from relatively recent (published 1999, 2001, 2006, 2012) work that may be specifically related to contexts from either before or after the Thera eruption. Samples identified as problematic because of unclear/insecure contexts or other issues in [68] are not used, nor dates on potentially problematic peat samples from lake contexts (e.g. the two TPQ dates from Gölhisar Gölu [206] – although it can be noted that including these two dates, as examples, which are stated as TPQs for the eruption, makes only a very small difference to the reported results, e.g. for Model 1 with the VERA-4630 TAQ as in Fig. 9 and Table 1, a re-run including these data, Beta-56673, 3300±70 ^14^C years BP and SRR-5188, 3225±45 ^14^C years BP, yields a Thera Eruption (Stage v) Boundary of 1606-1588 BCE (68.3% hpd), 1609-1559 BCE (95.4% hpd) in the model run reported in Fig. S2B, versus the average value for this Boundary of 1606-1589 BCE (68.3% hpd) and 1609-1560 BCE (95.4% hpd) as reported in Table 1).

| **No.** | **Date ID** | **Site** | **Archaeological Context** | **Material** | **^14^C Date**  **yrs BP** | **SD** | **Reference** |
| --- | --- | --- | --- | --- | --- | --- | --- |
| 204 | OxA-10319 | Akrotiri, Thera | M4N003 LCI, pre-VDL | *Olea europaea*, inner ‘rings’ 3-4 | 3424 | 38 | 64 |
| 205 | VERA-2747 | Akrotiri, Thera | M4N003 LCI, pre-VDL | *Olea europaea*, inner ‘rings’ 3-4 | 3386 | 30 | 64 |
| 206 | OxA-10316 | Akrotiri, Thera | M4N003 LCI, pre-VDL | *Olea europaea*, inner ‘rings’ 3-5 | 3342 | 38 | 64 |
| 207 | VERA-2744 | Akrotiri, Thera | M4N003 LCI, pre-VDL | *Olea europaea*, inner ‘rings’ 3-5 | 3427 | 31 | 64 |
| 208 | OxA-10318 | Akrotiri, Thera | M4N003 LCI, pre-VDL | *Olea europaea*, inner ‘rings’ 5-6 | 3355 | 40 | 64 |
| 209 | VERA-2746 | Akrotiri, Thera | M4N003 LCI, pre-VDL | *Olea europaea*, inner ‘rings’ 5-6 | 3471 | 28 | 64 |
| 210 | OxA-10315 | Akrotiri, Thera | M4N003 LCI, pre-VDL | *Olea europaea*, inner ‘rings’ 6-8bark | 3446 | 39 | 64 |
| 211 | VERA-2743 | Akrotiri, Thera | M4N003 LCI, pre-VDL | *Olea europaea*, inner ‘rings’ 6-8bark | 3413 | 28 | 64 |
| 212 | OxA-10317 | Akrotiri, Thera | M4N003 LCI, pre-VDL | *Olea europaea*, inner ‘rings’ 7-8bark | 3440 | 35 | 64 |
| 213 | VERA-2745 | Akrotiri, Thera | M4N003 LCI, pre-VDL | *Olea europaea*, inner ‘rings’ 7-8bark | 3386 | 28 | 64 |
| 214 | OxA-10314 | Akrotiri, Thera | 65/N001/I2 LCI, pre-VDL | *Tamarix* sp., ring 1 | 3330 | 27 | 64 |
| 215 | VERA-2751 | Akrotiri, Thera | 65/N001/I2 LCI, pre-VDL | *Tamarix* sp., ring 1 | 3325 | 28 | 64 |
| 216 | OxA-10313 | Akrotiri, Thera | 65/N001/I2 LCI, pre-VDL | *Tamarix* sp., ring 2 | 3353 | 27 | 64 |
| 217 | VERA-2749 | Akrotiri, Thera | 65/N001/I2 LCI, pre-VDL | *Tamarix* sp., ring 2 | 3335 | 33 | 64 |
| 218 | OxA-10312 | Akrotiri, Thera | 65/N001/I2 LCI, pre-VDL | *Tamarix* sp., ring 3bark | 3293 | 27 | 64 |
| 219 | VERA-2748 | Akrotiri, Thera | 65/N001/I2 LCI, pre-VDL | *Tamarix* sp., ring 3bark | 3319 | 28 | 64 |
| 220 | OxA-10730 | Trianda, Rhodes | Late MBA/Early LMIA AE1024 | *Quercus* sp., rings 1-10 | 3490 | 45 | 64 |
| 221 | OxA-11948 | Trianda, Rhodes | Late MBA/Early LMIA AE1024 | *Quercus* sp., rings 1-10 | 3526 | 25 | 64 |
| 222 | VERA-2742 | Trianda, Rhodes | Late MBA/Early LMIA  AE1024 | *Quercus* sp., rings 1-10 | 3476 | 28 | 64 |
| 223 | OxA-10729 | Trianda, Rhodes | Late MBA/Early LMIA  AE1024 | *Quercus* sp., rings 11-20 | 3410 | 45 | 64 |
| 224 | OxA-11946 | Trianda, Rhodes | Late MBA/Early LMIA  AE1024 | *Quercus* sp., rings 11-20 | 3474 | 24 | 64 |
| 225 | VERA-2741 | Trianda, Rhodes | Late MBA/Early LMIA  AE1024 | *Quercus* sp., rings 11-20 | 3485 | 28 | 64 |
| 226 | OxA-10728 | Trianda, Rhodes | Late MBA/Early LMIA  AE1024 | *Quercus* sp., rings 21-30bark | 3455 | 45 | 64 |
| 227 | OxA-11945 | Trianda, Rhodes | Late MBA/Early LMIA  AE1024 | *Quercus* sp., rings 21-30bark | 3473 | 24 | 64 |
| 228 | VERA-2740 | Trianda, Rhodes | Late MBA/Early LMIA  AE1024 | *Quercus* sp., rings 21-30bark | 3481 | 32 | 64 |
| 229 | OxA-11250 | Akrotiri, Thera | Late Middle Cycladic (LMC)/Earlier LCI | Wood charcoal | 3550 | 45 | 64 |
| 230 | Hd-22037 | Akrotiri, Thera | Late Middle Cycladic/Earlier LCI | Wood charcoal | 3552 | 19 | 64 |
| 231 | DEM-1458 | Akrotiri, Thera | Late Middle Cycladic | Wood charcoal | 3375 | 25 | 78 |
| 232 | DEM-1528 | Akrotiri, Thera | Before LC? (so LMC) | Wood charcoal | 3462 | 25 | 78 |
| 233 | DEM-1531 | Akrotiri, Thera | Late Middle Cycladic | Wood charcoal | 3441 | 25 | 78 |
| 234 | DEM-1623 | Akrotiri, Thera | Late Middle Cycladic | Wood charcoal | 3499 | 25 | 78 |
| 235 | DEM-89 | Trianda, Rhodes | Earlier LMIA | Wood charcoal | 3517 | 83 | 73 |
| 236 | DEM-859 | Trianda, Rhodes | Earlier LMIA | Wood charcoal | 3568 | 44 | 73 |
| 237 | DEM-1313 | Akrotiri, Thera | LCI (pre-LCI Advanced/VDL) M20/43N045 | Wood charcoal | 3396 | 25 | 78 |
| 238 | DEM-1314 | Akrotiri, Thera | LCI (pre-LCI Advanced/VDL) M18/43N045 | Wood charcoal | 3467 | 25 | 78 |
| 239 | DEM-1345 | Akrotiri, Thera | LCI (pre-LCI Advanced/VDL) M21/43N047 | Wood charcoal | 3441 | 25 | 78 |
| 240 | DEM-1455 | Akrotiri, Thera | LCI (pre-LCI Advanced/VDL) M22/53AN023 | Wood charcoal | 3508 | 25 | 78 |
| 241 | DEM-1456 | Akrotiri, Thera | LCI (pre-LCI Advanced/VDL) M18/53AN018 | Wood charcoal | 3456 | 25 | 78 |
| 242 | DEM-1457 | Akrotiri, Thera | LCI (pre-LCI Advanced/VDL) M15/53AN017 | Wood charcoal | 3436 | 25 | 78 |
| 243 | DEM-1609 | Akrotiri, Thera | LCI (pre-LCI Advanced/VDL) M12/53AN016 | Wood charcoal | 3433 | 25 | 78 |
| 244 | DEM-1610 | Akrotiri, Thera | LCI (pre-LCI Advanced/VDL) I3/53AN015 | Wood charcoal | 3420 | 25 | 78 |
| 245 | DEM-1646 | Akrotiri, Thera | LCI (pre-LCI Advanced/VDL) I1/61N001 W.S. | Wood charcoal | 3508 | 25 | 78 |
| 246 | DEM-828 | Trianda, Rhodes | Liamis property Room II, Layer ΣΤ’ Depth 3.59-3.66m Mature LBIA/LMIA | Wood charcoal | 3407 | 25 | 73 |
| 247 | DEM-830 | Trianda, Rhodes | Platsis property AE56, Western stone heap trench Layer V Depth 3.88-4.00m Mature LBIA/LMIA | Wood charcoal | 3449 | 21 | 73 |
| 248 | DEM-831 | Trianda, Rhodes | Platsis property AE27, Destruction layer northwards wall Nr 4 Depth 2.65-2.98m Mature LBIA/LMIA | Wood charcoal | 3466 | 23 | 73 |
| 249 | OxA-11894 | Miletos, Turkey | LMIA | Animal bone, *Ovis/Capra* | 3377 | 24 | 64 |
| 250 | OxA-11951 | Miletos, Turkey | LMIA | Animal bone, *Ovis/Capra* | 3423 | 23 | 64 |
| 251 | DEM-90 | Trianda, Rhodes | Markos property AE273, Square AB1, Layer Δ’ Depth 2.46-2.68m LMIB | Wood charcoal | 3258 | 54 | 73 |
| 252 | DEM-91 | Trianda, Rhodes | Markos property AE273, Square AB1, Layer Δ’ Depth 2.46-2.68m, LMIB | Wood charcoal | 3240 | 35 | 73 |
| 253 | DEM-856 | Trianda, Rhodes | Liamis property AE224, Square Γ2, Layer E’, from pit Depth 3.10-3.30m, LMIB | Wood charcoal | 3175 | 41 | 73 |
| 254 | DEM-829 | Trianda, Rhodes | Liamis property Square Γ2, Layer ΣΤ’ Depth 2.77-2.93/3.03m, LMIB, LMIB | Wood charcoal | 3171 | 33 | 73 |
| 255 | DEM-857 | Trianda, Rhodes | Liamis property AE236, Square Γ2, Layer Ηæ, from pit Depth 3.84-4.00m, LMIB | Wood charcoal | 3142 | 52 | 73 |
| 256 | DEM-858 | Trianda, Rhodes | Liamis property AE329, Square B2, under the floor Depth 3.03-3.16m, LMIB | Wood charcoal | 3138 | 50 | 73 |
| 257 | OxA-3674 | Kommos, Crete | LMII, Hillside 41A1/26 | Animal bone, charred | 3090 | 80 | 9 |

**Additional Reference [210] for Table S1**

1. Soles JS. Radiocarbon results. In Soles JS et al. Mochlos IC. Period III. Neopalatial settlement on the coast: the Artisan’s Quarter and the Farmhouse at Chalinomouri. The small finds. Philadelphia: INSTAP Academic Press; 2004. pp.145-149.
