# Supplementary material for: Second Intermediate Period date for the Thera (Santorini) eruption and historical implications
Source: PLoS One. 2022 Sep 20;17(9):e0274835. doi: 10.1371/journal.pone.0274835 (PMC9488803; doi:10.1371/journal.pone.0274835)
Supplement: S2 Table — (DOCX) [file pone.0274835.s002.docx]

Table S2. OxCal runfiles for the models used in this paper.

Dataset (a)

Options()

{

Resolution=1;

kIterations=3000;

};

Plot()

{

Outlier_Model("General",T(5),U(0,4),"t");

Outlier_Model("SSimple",N(0,2),0,"s");

D_Sequence("Miletos WM, Quercus sp.")

{

R_Combine("RY1000-RY1010")

{

Outlier ("SSimple",0.05);

R_Date("OxA-12301", 3439, 30)

{

Outlier ("SSimple",0.05);

};

R_Date("OxA-12302", 3386, 31)

{

Outlier ("SSimple",0.05);

};

};

Gap(10);

R_Combine("RY1010-RY1020")

{

Outlier ("SSimple",0.05);

R_Date("OxA-12303", 3467, 31)

{

Outlier ("SSimple",0.05);

};

R_Date("OxA-12407", 3385, 34)

{

Outlier ("SSimple",0.05);

};

};

Gap(10);

R_Combine("RY1020-RY1030")

{

Outlier ("SSimple",0.05);

R_Date("OxA-12304", 3404, 31)

{

Outlier ("SSimple",0.05);

};

R_Date("OxA-12305", 3459, 31)

{

Outlier ("SSimple",0.05);

};

};

Gap(10);

R_Combine("RY1030-RY1040")

{

Outlier ("SSimple",0.05);

R_Date("OxA-12306", 3416, 31)

{

Outlier ("SSimple",0.05);

};

R_Date("OxA-12307", 3425, 31)

{

Outlier ("SSimple",0.05);

};

};

Gap(10);

R_Combine("RY1040-RY1050")

{

Outlier ("SSimple",0.05);

R_Date("OxA-12308", 3361, 31)

{

Outlier ("SSimple",0.05);

};

R_Date("OxA-12309", 3397, 32)

{

Outlier ("SSimple",0.05);

};

};

Gap(10);

R_Combine("RY1050-RY1060")

{

Outlier ("SSimple",0.05);

R_Date("OxA-12310", 3345, 32)

{

Outlier ("SSimple",0.05);

};

R_Date("OxA-12311", 3397, 32)

{

Outlier ("SSimple",0.05);

};

};

Gap(10);

R_Combine("RY1060-RY1070")

{

Outlier ("SSimple",0.05);

R_Date("OxA-12312", 3388, 30)

{

Outlier ("SSimple",0.05);

};

R_Date("OxA-12313", 3352, 31)

{

Outlier ("SSimple",0.05);

};

};

Gap(7);

Date("Felling Miletos WM");

//waney edge

};

Sequence("Thera/Santorini Eruption/Tsunami *no* Thera/Santorini Data")

{

Tau_Boundary ("TnoT");

Phase ("Close TPQs for VDL or even date of VDL")

{

Label("LBIA/LMIA mature and destruction later below Thera tephra, Trianda, Rhodes, so TPQ");

R_Date ("DEM-94 charcoal", 3347,46)

{

Outlier ("General",0.05);

};

R_Date ("DEM-93 charcoal", 3358,48)

{

Outlier ("General",0.05);

};

R_Combine("Trianda short-lived late LMIA twig, pre-Thera-tephra, Quercus sp.")

{

Outlier ("General",0.05);

R_Date("OxA-10643", 3367, 39)

{

Outlier("SSimple",0.05);

};

R_Date("OxA-11884", 3344, 32)

{

Outlier("SSimple",0.05);

};

};

Date("=Felling Miletos WM");

Label("Thera Tsunami Palaikastro");

Label("Palaikastro Promontory");

R_Date("GrA-30336 Cattle bone",3310,35)

{

Outlier ("General",0.05);

};

R_Date("GrA-30339 Cattle bone",3390,35)

{

Outlier ("General",0.05);

};

Label("Bone/jaw, tooth Palaikastro drain with stratified Thera tephra");

R_Date("GrA-28991 goat/sheep bone/jaw",3325,40)

{

Outlier ("General",0.05);

};

R_Date("GrA-29041 goat/sheep bone/jaw",3345,40)

{

Outlier ("General",0.05);

};

R_Date("GrA-29042 tooth",3385,40)

{

Outlier ("General",0.05);

};

Label("Thera Tsunami Çeşme-Bağlararası");

R_Date("OxA-38858 H1a S",3275,17)

{

Outlier ("General",0.05);

};

R_Date("OxA-38881 H1a B",3367,22)

{

Outlier ("General",0.05);

};

R_Date("OxA-38973 H1a C",3318,19)

{

Outlier ("General",0.05);

};

R_Date("OxA-38972 H1a B",3316,20)

{

Outlier ("General",0.05);

};

R_Date("OxA-38857 H1a S",3312,17)

{

Outlier ("General",0.05);

};

R_Date("OxA-38950 H1b C",3384,22)

{

Outlier ("General",0.05);

};

R_Date("D-AMS019172 H1b B",3372,27)

{

Outlier ("General",0.05);

};

R_Date("OxA-38966 H1c C",3297,19)

{

Outlier ("General",0.05);

};

R_Date("D-AMS019173 H1c C",3291,30)

{

Outlier ("General",0.05);

};

Label(“Thera Tsunami Eşençay Delta”);

R_Date("Lyon7920 organic-rich layer",3295,30)

{

Outlier ("General",0.05);

};

};

Boundary("EnoT");

};

};

Note: EnoT = Thera Eruption with no samples from Thera/Santorini

Dataset (b)

Options()

{

Resolution=1;

kIterations=3000;

};

Plot()

{

Outlier_Model("General",T(5),U(0,4),"t");

Sequence("Akrotiri Stages 2/3")

{

Tau_Boundary ("Stages 2/3");

Phase ("Akrotiri secure Stages 2/3 Food Products in Use or Storage or LCI Adv charcoal")

{

R_Date("OxA-1552 Lathyrus sp.", 3390, 65)

{

Outlier ("General",0.05);

};

R_Date("OxA-1555 Lathyrus sp.", 3245, 65)

{

Outlier ("General",0.05);

};

R_Date("OxA-1548 Lathyrus sp.", 3335, 60)

{

Outlier ("General",0.05);

};

R_Date("OxA-1549 Lathyrus sp.", 3460, 80)

{

Outlier ("General",0.05);

};

R_Date("OxA-1550 Lathyrus sp.", 3395, 65)

{

Outlier ("General",0.05);

};

R_Date("OxA-1553 Lathyrus sp.", 3340, 65)

{

Outlier ("General",0.05);

};

R_Date("OxA-1554 Lathyrus sp.", 3280, 65)

{

Outlier ("General",0.05);

};

R_Date("OxA-1556 Hordeum sp.", 3415, 70)

{

Outlier ("General",0.05);

};

R_Date("K-5352 pulses", 3310, 65)

{

Outlier ("General",0.05);

};

R_Date("K-3228 pulses", 3340, 55)

{

Outlier ("General",0.05);

};

R_Date("OxA-11817 ?Lathyrus sp.", 3348, 31)

{

Outlier ("General",0.05);

};

R_Date("OxA-11818 Hordeum sp.", 3367, 33)

{

Outlier ("General",0.05);

};

R_Date("OxA-11820 Hordeum sp.", 3400, 31)

{

Outlier ("General",0.05);

};

R_Date("OxA-11869 Hordeum sp.", 3336, 34)

{

Outlier ("General",0.05);

};

R_Date("OxA-12170 ?Lathyrus sp.", 3336, 28)

{

Outlier ("General",0.05);

};

R_Date("OxA-12171 Hordeum sp.", 3372, 28)

{

Outlier ("General",0.05);

};

R_Date("OxA-12175 Hordeum sp.", 3318, 28)

{

Outlier ("General",0.05);

};

R_Date("OxA-12172 Hordeum sp.", 3321, 32)

{

Outlier ("General",0.05);

};

R_Date("VERA-2756 Hordeum sp.", 3317, 28)

{

Outlier ("General",0.05);

};

R_Date("VERA-2757 ?Lathyrus sp.", 3315, 31)

{

Outlier ("General",0.05);

};

R_Date("VERA-2758 Hordeum sp.", 3339, 28)

{

Outlier ("General",0.05);

};

R_Date("VERA-2757 repeat ?Lathyrus sp.", 3390, 32)

{

Outlier ("General",0.05);

};

R_Date("VERA-2758 repeat Hordeum sp.", 3322, 32)

{

Outlier ("General",0.05);

};

R_Date ("OxA-25176 insect chitin", 3368, 29)

{

Outlier ("General",0.05);

};

Label("Hd data on SL samples from Akrotiri VDL stages 2/3");

R_Date("Hd-7092-6795 peas",3360,60)

{

Outlier ("General",0.05);

};

Label("LCI Advanced or Advanced? or VDL charcoal, Akrotiri, Thera");

R_Date ("DEM-1311 LC I VDL", 3307, 25)

{

Outlier ("General",0.05);

};

R_Date ("DEM-1529 LC I Advanced? VDL", 3281,25)

{

Outlier ("General",0.05);

};

R_Date ("DEM-1607 LC I Advanced VDL", 3228,30)

{

Outlier ("General",0.05);

};

R_Date ("DEM-1624 LC I Advanced VDL", 3360,25)

{

Outlier ("General",0.05);

};

R_Date ("DEM-1615 LC I Advanced? VDL", 3389,25)

{

Outlier ("General",0.05);

};

R_Date("K-4255 twig in pumice so assume LCI Advanced", 3380, 60)

{

Outlier ("General",0.05);

};

};

Boundary("E2/3");

};

};

Notes:

1. E2/3 = End Boundary for Akrotiri stages (ii)/(iii) or 2/3.

2. Sample K-5353 pulses (no.84 Table S1) is not included as it is unclear from the available information provided whether this sample is from a secure stages (ii)/(iii) (or 2/3) context. Samples Hd-6058-5519 grains and Hd-6059-7967 grains (nos. 99, 100 in Table S1) are also not included as it is not clear from the available information whether these samples derive from a secure stages (ii)/(iii) (or 2/3) context.

Dataset (c)

Options()

{

Resolution=1;

kIterations=3000;

};

Plot()

{

Outlier_Model("General",T(5),U(0,4),"t");

Sequence("Friedrich et al. [80] olive as Sequence only")

{

Boundary("Start");

Sequence()

{

R_Date("Hd-23599-24426 'rings' 1-13", 3383, 11)

{

Outlier ("General",0.05);

};

R_Date("Hd-23587 'rings' 14-37", 3372, 12)

{

Outlier ("General",0.05);

};

R_Date("Hd-23589 'rings' 38-59", 3349, 12)

{

Outlier ("General",0.05);

};

R_Date("Hd-23588-24402 'rings' 60-72", 3331, 10)

{

Outlier ("General",0.05);

};

//Only Sequence, no ‘rings’ used

};

Boundary("EOlive");

};

Sequence("Other Thera/Santorini eruption olive wood from pumice")

{

Tau_Boundary ("Tolive");

Phase ("Olive from pumice")

{

Label("Olive branch or tree root samples from Thera/Santorini pre-eruption ABA and HS TPQ for eruption");

R_Date("VERA-5614 ABA olive branch",3282,21)

{

Outlier ("General",0.05);

};

R_Date("VERA-5614HS olive branch",3359,33)

{

Outlier ("General",0.05);

};

R_Date("VERA-5615 ABA olive branch", 3280,24)

{

Outlier ("General",0.05);

};

R_Date("VERA-5615HS olive branch", 3321,24)

{

Outlier ("General",0.05);

};

R_Date("VERA-5620 ABA olive branch",3277,25)

{

Outlier ("General",0.05);

};

R_Date("VERA-5620HS olive branch",3345,24)

{

Outlier ("General",0.05);

};

R_Date("VERA-5610 ABA olive branch",3399,25)

{

Outlier ("General",0.05);

};

R_Date("VERA-5610HS olive branch",3342,26)

{

Outlier ("General",0.05);

};

R_Date("VERA-5083 ABA olive branch",3270,36)

{

Outlier ("General",0.05);

};

R_Date("VERA-5083HS olive branch",3326,77)

{

Outlier ("General",0.05);

};

R_Date("VERA-5082 ABA olive branch",3332,38)

{

Outlier ("General",0.05);

};

R_Date("VERA-5082HS olive branch",3369,36)

{

Outlier ("General",0.05);

};

R_Date("VERA-5084 ABA root",3354,32)

{

Outlier ("General",0.05);

};

R_Date("VERA-5084HS root",3368,34)

{

Outlier ("General",0.05);

};

};

Boundary("=EOlive");

};

};

Note: EOlive = Thera Eruption Boundary from Olive wood samples (from Thera from VDL/stage (v) contexts).

Dataset (d)

Options()

{

Resolution=1;

kIterations=3000;

};

Plot()

{

Outlier_Model("General",T(5),U(0,4),"t");

Sequence("Thera/Santorini VDL Data from archaeological or older work – not olive samples")

{

Tau_Boundary ("Tdatasetd");

Phase ("Close TPQs for VDL or even date of VDL - all data")

{

//includes Oxford stages 1/2 dates since comparable contexts to some of the other LMIA destruction dates from Akrotiri

Label("LCI Advanced or Advanced? or VDL charcoal, Akrotiri, Thera");

R_Date ("DEM-1311 LC I VDL", 3307, 25)

{

Outlier ("General",0.05);

};

R_Date ("DEM-1529 LC I Advanced? VDL", 3281,25)

{

Outlier ("General",0.05);

};

R_Date ("DEM-1607 LC I Advanced VDL", 3228,30)

{

Outlier ("General",0.05);

};

R_Date ("DEM-1624 LC I Advanced VDL", 3360,25)

{

Outlier ("General",0.05);

};

R_Date ("DEM-1615 LC I Advanced? VDL", 3389,25)

{

Outlier ("General",0.05);

};

Label("pre-1980 data re VDL");

//Akrotiri or Thera VDL or under Minoan Pumice dates published before 1980 and not stated as undersized or clearly outlier or not then recognized as from likely older contexts (see also d and u in Housley et al. [68])

R_Date("K-3227 Athinios Quarry charcoal",3400,70)

{

Outlier ("General",0.05);

};

R_Date("P-2794 charcoal",3180,50)

{

Outlier ("General",0.05);

};

R_Date("P-1890 charcoal",3340,60)

{

Outlier ("General",0.05);

};

R_Date("L-362 Phira quarry charcoal",3370,100)

{

Outlier ("General",0.05);

};

R_Date("P-1401 Phira quarry charcoal",3420,40)

{

Outlier ("General",0.05);

};

R_Date("P-1697 beans",3070,60)

{

Outlier ("General",0.05);

};

R_Date("P-1885 charred seeds",3250,50)

{

Outlier ("General",0.05);

};

R_Date("P-1888 shrubs",3130,50)

{

Outlier ("General",0.05);

};

R_Date("P-1889 shrubs",3300,50)

{

Outlier ("General",0.05);

};

R_Date("P-1892 shrubs?",3330,50)

{

Outlier ("General",0.05);

};

R_Date("P-1894 shrubs",3310,70)

{

Outlier ("General",0.05);

};

R_Date("P-1895 shrubs?",3320,50)

{

Outlier ("General",0.05);

};

R_Date("P-2559 grain",3370,70)

{

Outlier ("General",0.05);

};

R_Date("P-2565 grain",3310,60)

{

Outlier ("General",0.05);

};

R_Date("P-2791 seeds & charred material",3340,60)

{

Outlier ("General",0.05);

};

Label("Three Hd data on SL samples from Akrotiri VDL");

R_Date("Hd-7092-6795 peas",3360,60)

{

Outlier ("General",0.05);

};

R_Date("Hd-6058-5519 grains",3490,80)

{

Outlier ("General",0.05);

};

R_Date("Hd-6059-7967 grains",3140,70)

{

Outlier ("General",0.05);

};

R_Date("OxA-1552 Lathyrus sp.", 3390, 65)

{

Outlier ("General",0.05);

};

R_Date("OxA-1555 Lathyrus sp.", 3245, 65)

{

Outlier ("General",0.05);

};

R_Date("OxA-1548 Lathyrus sp.", 3335, 60)

{

Outlier ("General",0.05);

};

R_Date("OxA-1549 Lathyrus sp.", 3460, 80)

{

Outlier ("General",0.05);

};

R_Date("OxA-1550 Lathyrus sp.", 3395, 65)

{

Outlier ("General",0.05);

};

R_Date("OxA-1553 Lathyrus sp.", 3340, 65)

{

Outlier ("General",0.05);

};

R_Date("OxA-1554 Lathyrus sp.", 3280, 65)

{

Outlier ("General",0.05);

};

R_Date("OxA-1556 Hordeum sp.", 3415, 70)

{

Outlier ("General",0.05);

};

R_Date("K-5352 pulses", 3310, 65)

{

Outlier ("General",0.05);

};

R_Date("K-5353 pulses", 3430, 90)

{

Outlier ("General",0.05);

};

R_Date("K-3228 pulses", 3340, 55)

{

Outlier ("General",0.05);

};

R_Date("K-4255 twig", 3380, 60)

{

Outlier ("General",0.05);

};

R_Date("OxA-11817 ?Lathyrus sp.", 3348, 31)

{

Outlier ("General",0.05);

};

R_Date("OxA-11818 Hordeum sp.", 3367, 33)

{

Outlier ("General",0.05);

};

R_Date("OxA-11820 Hordeum sp.", 3400, 31)

{

Outlier ("General",0.05);

};

R_Date("OxA-11869 Hordeum sp.", 3336, 34)

{

Outlier ("General",0.05);

};

R_Date("OxA-12170 ?Lathyrus sp.", 3336, 28)

{

Outlier ("General",0.05);

};

R_Date("OxA-12171 Hordeum sp.", 3372, 28)

{

Outlier ("General",0.05);

};

R_Date("OxA-12175 Hordeum sp.", 3318, 28)

{

Outlier ("General",0.05);

};

R_Date("OxA-12172 Hordeum sp.", 3321, 32)

{

Outlier ("General",0.05);

};

R_Date("VERA-2756 Hordeum sp.", 3317, 28)

{

Outlier ("General",0.05);

};

R_Date("VERA-2757 ?Lathyrus sp.", 3315, 31)

{

Outlier ("General",0.05);

};

R_Date("VERA-2758 Hordeum sp.", 3339, 28)

{

Outlier ("General",0.05);

};

R_Date("VERA-2757 repeat ?Lathyrus sp.", 3390, 32)

{

Outlier ("General",0.05);

};

R_Date("VERA-2758 repeat Hordeum sp.", 3322, 32)

{

Outlier ("General",0.05);

};

R_Date ("OxA-25176 insect chitin", 3368, 29)

{

Outlier ("General",0.05);

};

Label("Oxford VDL stages 1/2 dates");

R_Date("OxA-1558 legumes",3400,60)

{

Outlier ("General",0.05);

};

R_Date("OxA-1551 Hordeum sp.",3465,65)

{

Outlier ("General",0.05);

};

R_Date("OxA-1557 legumes",3240,60)

{

Outlier ("General",0.05);

};

Label("The 'residue' series II dates from Housley et al. 1990 [68] stages 1/2 and 2/3");

R_Date("OxA-1687 Lathyrus sp.",3280,60)

{

Outlier ("General",0.05);

};

R_Date("OxA-1689 Lathyrus sp.",3270,60)

{

Outlier ("General",0.05);

};

R_Date("OxA-1691 Lathyrus sp.",3320,60)

{

Outlier ("General",0.05);

};

R_Date("OxA-1692 Lathyrus sp.",3325,90)

{

Outlier ("General",0.05);

};

Label("Set of ETH dates listed as Akrotiri LMIA destruction, so 'VDL', in Kutschera and Stadler [88]; use only dates on short-lived samples");

R_Date("ETH-3315 seed",3610,51)

{

Outlier ("General",0.05);

};

R_Date("ETH-3323 seed",3437,54)

{

Outlier ("General",0.05);

};

R_Date("ETH-3324 seed",3453,52)

{

Outlier ("General",0.05);

};

};

Boundary("EallS");

};

};

Note: EallT = Thera Eruption Boundary from all samples from Thera/Santorini from stages (ii)/(iii) or (i)/(ii) or from VDL or likely VDL or LCI advanced or similar contexts and not likely residual or older initial use/procurement material.

Datasets (e) to (h)

The respective OxCal runfiles are identical to those above except for the Delta_R line added at the beginning of each file, e.g.:

Plot()

{

Delta_R("Aegean Max Test",4,2);

**Dataset (i)**

Options()

{

Resolution=1;

kIterations=3000;

};

Plot("Dataset i")

{

//S or SL = short-lived plant material (e.g. seeds or plant matter), B = animal bone, C = charcoal (apparently not very long-lived treat as SL, T = wood twig, so shorter-lived)

Outlier_Model("General",T(5),U(0,4),"t");

Outlier_Model("SSimple",N(0,2),0,"s");

Sequence("Dataset i: Kolonna, Aegina for MH/LHI, Phases J/K, Transition")

{

Boundary("Begin E");

Phase("Phase E")

{

Sequence()

{

Combine("fire destruction")

{

Outlier ("General",0.05);

R_Date("VERA-2678 S", 3724, 35)

{

Outlier("SSimple",0.05);

};

R_Date("VERA-2680 S", 3722, 35)

{

Outlier("SSimple",0.05);

};

R_Date("VERA-2681 S", 3739, 35)

{

Outlier("SSimple",0.05);

};

R_Date("VERA-2679 S", 3761, 35)

{

Outlier("SSimple",0.05);

};

R_Date("VRI-0395 C", 3670, 90)

{

Outlier("SSimple",0.05);

};

R_Date("HV-5841 C", 3625, 65)

{

Outlier("SSimple",0.05);

};

R_Date("VERA-2682 S", 3712, 35)

{

Outlier("SSimple",0.05);

};

R_Date("VERA-2683 S", 3721, 35)

{

Outlier("SSimple",0.05);

};

R_Date("HV-5840 C", 3820, 65)

{

Outlier("SSimple",0.05);

};

};

R_Date("VERA-4641 B", 3759, 35)

{

Outlier ("General",0.05);

};

};

R_Date("VERA-2688 B", 3698, 33)

{

Outlier ("General",0.05);

};

};

Boundary("Transition E/F");

Phase("Phase F")

{

R_Date("VERA-2692 B", 3704, 36)

{

Outlier ("General",0.05);

};

};

Boundary("Transition F/G");

Phase("Phase G")

{

Sequence("Sequence")

{

R_Date("VERA-4640 B", 3800, 44)

{

Outlier ("General",0.05);

};

R_Date("VERA-4639 B", 3809, 32)

{

Outlier ("General",0.05);

};

};

R_Date("VERA-4638 B", 3646, 32)

{

Outlier ("General",0.05);

};

R_Date("VERA-4281 S", 3740, 36)

{

Outlier ("General",0.05);

};

R_Date("VERA-4282 S", 3711, 34)

{

Outlier ("General",0.05);

};

R_Date("VERA-4283 S", 3780, 37)

{

Outlier ("General",0.05);

};

};

Boundary("Transition G/H");

Phase("Phase H")

{

Sequence("Sequence")

{

R_Date("VERA-4637 B", 3643, 30)

{

Outlier ("General",0.05);

};

R_Date("VERA-4636 B", 3628, 30)

{

Outlier ("General",0.05);

};

};

Sequence("Sequence")

{

R_Date("VERA-4280 S", 3724, 39)

{

Outlier ("General",0.05);

};

R_Date("VERA-4279 S", 3718, 38)

{

Outlier ("General",0.05);

};

};

R_Date("VERA-2687", 3694 B, 35)

{

Outlier ("General",0.05);

};

};

Boundary("Transition H/I");

Phase("Phase I")

{

Sequence()

{

Phase("before Minoan layer")

{

R_Date("VERA-4634 B", 3544, 37)

{

Outlier ("General",0.05);

};

R_Date("VERA-4278 S", 3522, 38)

{

Outlier ("General",0.05);

};

};

Combine("Minoan layer")

{

Outlier ("General",0.05);

R_Date("VERA-4038 S", 3506, 34)

{

Outlier("SSimple",0.05);

};

R_Date("VERA-4576 B", 3482, 37)

{

Outlier("SSimple",0.05);

};

R_Date("VERA-4575 B", 3537, 36)

{

Outlier("SSimple",0.05);

};

R_Date("VERA-4578 B", 3501, 39)

{

Outlier("SSimple",0.05);

};

R_Date("VERA-4579 B", 3526, 38)

{

Outlier("SSimple",0.05);

};

R_Date("VERA-4580 B", 3506, 33)

{

Outlier("SSimple",0.05);

};

R_Date("VERA-4276 S", 3506, 37)

{

Outlier("SSimple",0.05);

};

R_Date("VERA-4275 S", 3544, 38)

{

Outlier("SSimple",0.05);

};

};

R_Date("VERA-4577 B", 3458, 39)

{

Outlier ("General",0.05);

};

};

};

Boundary("Transition I/J");

Phase("Phase J = MHIII")

{

Sequence()

{

Combine()

{

Outlier ("General",0.05);

R_Date("VERA-4571 B", 3469, 38)

{

Outlier("SSimple",0.05);

};

R_Date("VERA-4574 B", 3430, 39)

{

Outlier("SSimple",0.05);

};

R_Date("VERA-4573 B", 3485, 36)

{

Outlier("SSimple",0.05);

};

};

Phase()

{

R_Date("VERA-4572 B", 3407, 38)

{

Outlier ("General",0.05);

};

R_Date("VERA-4570 B", 3428, 36)

{

Outlier ("General",0.05);

};

};

};

};

Boundary("Kolonna Transition J/K MH/LHI");

Phase("Phase K - LHI")

{

Sequence()

{

R_Date("VERA-4633 B", 3333, 29)

{

Outlier ("General",0.05);

};

R_Date("VERA-4632 B", 3356, 36)

{

Outlier ("General",0.05);

};

R_Date("VERA-4631 B", 3349, 36)

{

Outlier ("General",0.05);

};

};

};

Boundary("Kolonna Transition K/L");

Phase("Phase L - LHII")

{

R_Date("VERA-4630 B", 3313, 48)

{

Outlier ("General",0.05);

};

};

Boundary("End Kolonna L: End LHII");

Boundary("Begin Kolonna M: LHIIIA");

Phase("Phase M - LHIIIA")

{

Combine()

{

Outlier ("General",0.05);

R_Date("VERA-4284 S", 3044, 35)

{

Outlier("SSimple",0.05);

};

R_Date("VERA-4582 T", 2986, 33)

{

Outlier("SSimple",0.05);

};

R_Date("VERA-4285 S", 3040, 37)

{

Outlier("SSimple",0.05);

};

};

};

Boundary("End Phase M");

};

};

**Dataset (j)**

Options()

{

Resolution=1;

kIterations=3000;

};

Plot("Dataset i")

{

Outlier_Model("General",T(5),U(0,4),"t");

Outlier_Model("SSimple",N(0,2),0,"s");

Sequence("Dataset j, Kommos early LMIA")

{

Boundary("Start Kommos Early LMIA charcoal TPQ Early LMIA");

Phase( "Kommos early LMIA secure charcoal longer-lived, TPQs")

{

R_Combine("K85A/62D/9:92 Quercus sp.")

{

Outlier ("General",0.05);

R_Date("OxA-11251", 3505, 40)

{

Outlier("SSimple",0.05);

};

R_Date("VERA-2636", 3445, 25)

{

Outlier("SSimple",0.05);

};

};

R_Combine ("K85A/62D/8:83 Quercus sp.")

{

Outlier ("General",0.05);

R_Date("OxA-11253", 3397, 38)

{

Outlier("SSimple",0.05);

};

R_Date("VERA-2638", 3600, 19)

{

Outlier("SSimple",0.05);

};

//Same sample but X2 22.484 > 3.8 - given the other data assume VERA-2638 must be much too old outlier

};

R_Combine("Space 25B, Tr.66B likely Cupressaceae")

{

Outlier ("General",0.05);

R_Date("OxA-11883", 3485, 33)

{

Outlier("SSimple",0.05);

};

R_Date("OxA-11944", 3435, 25)

{

Outlier("SSimple",0.05);

};

R_Date("OxA-3429", 3350, 70)

{

Outlier("SSimple",0.05);

};

};

};

Boundary("longer-lived samples to shorter-lived twig and likely age Early LMIA");

R_Combine("Kommos Early LMIA charred twig - Likely Early LMIA Date")

{

Outlier ("General",0.05);

R_Date("OxA-11252 K85A/66B/4:22+23 twig", 3375, 45)

{

Outlier("SSimple",0.05);

};

R_Date("VERA-2637 K85A/66B/4:22+23 twig", 3390, 20)

{

Outlier("SSimple",0.05);

};

};

Boundary("End early LMIA Kommos data");

};

};

**Dataset (k – part 1)**

Options()

{

Resolution=1;

kIterations=3000;

};

Plot("Lerna Shaft Grave 1")

{

Outlier_Model("General",T(5),U(0,4),"t");

Sequence("Lerna Shaft Grave 1 Mid-LHI animal bone")

{

Tau_Boundary("T");

Phase ("SG1")

{

//R_Date("OxA-20127 bone",3522,27)

//{

// Outlier ("General",0.05);

//};

//R_Date("OxA-20128 bone",3479,27)

//{

// Outlier ("General",0.05);

//};

R_Date("OxA-20170 bone",3360,29)

{

Outlier ("General",0.05);

};

R_Date("OxA-22634 bone",3314,27)

{

Outlier ("General",0.05);

};

R_Date("OxA-22635 bone",3391,26)

{

Outlier ("General",0.05);

};

R_Date("OxA-X-2304-54 bone",3378,28)

{

Outlier ("General",0.05);

};

};

Boundary("E");

};

Tau=(E-T);

Tau&= U(0,20);

};

Note: the run used in Fig. 10 excludes the substantially older OxA-20127 and OxA-20128 samples – see the discussion in Materials and methods. This model version shown.

**Dataset (k – part 2)**

Options()

{

Resolution=1;

kIterations=3000;

};

Plot("Lerna Shaft Grave 2")

{

Outlier_Model("General",T(5),U(0,4),"t");

Sequence("Lerna Shaft Grave 2 Late LHI animal bone")

{

Tau_Boundary("T");

Phase("SG2")

{

R_Date("OxA-20126 bone",3356,27)

{

Outlier ("General",0.05);

};

R_Date("OxA-20168 bone",3326,28)

{

Outlier ("General",0.05);

};

R_Date("OxA-20169 bone",3393,31)

{

Outlier ("General",0.05);

};

R_Date("OxA-22631 bone",3276,27)

{

Outlier ("General",0.05);

};

R_Date("OxA-22632 bone",3313,27)

{

Outlier ("General",0.05);

};

R_Date("OxA-22633 bone",3275,26)

{

Outlier ("General",0.05);

};

};

Boundary("E");

};

Tau=(E-T);

Tau&= U(0,20);

};

**Dataset (l)**

Options()

{

Resolution=1;

kIterations=3000;

};

Plot()

{

Outlier_Model("General",T(5),U(0,4),"t");

Sequence("LMIB to LMII Destruction Datasets")

{

Boundary();

Phase("LMIB Destruction Data Sets")

{

Sequence ("Chania LMIB Destruction")

{

Tau_Boundary ("T Chania");

Phase ("Chania, all charred short-lived plant matter")

{

R_Date("OxA-2517 Pisum sativum", 3380, 80)

{

Outlier ("General",0.05);

};

R_Date("OxA-2518 Vicia faba", 3340, 80)

{

Outlier ("General",0.05);

};

R_Date("OxA-2646 Hordeum sp.", 3315, 70)

{

Outlier ("General",0.05);

};

R_Date("OxA-2647 charred seed", 3150, 70)

{

Outlier ("General",0.05);

};

R_Date("OxA-10320 Vicia faba", 3208, 26)

{

Outlier ("General",0.05);

};

R_Date("OxA-10321 Horedeum sp.", 3268, 27)

{

Outlier ("General",0.05);

};

R_Date("OxA-10322 Pisum sativum", 3338, 26)

{

Outlier ("General",0.05);

};

R_Date("OxA-10323 charred seed", 3253, 25)

{

Outlier ("General",0.05);

};

};

Boundary ("CD");

};

Sequence ("Sequence of LMIB Late to LMIB Final Destructions")

{

Sequence("Myrtos-Pyrgos (LMIB Late) Destruction")

{

Tau_Boundary ("T Myrtos-Pyrgos");

Phase ("Myrtos-Pyrgos, charred short-lived plant material")

{

R_Date("OxA-3187 Hordeum sp.", 3230, 70)

{

Outlier ("General",0.05);

};

R_Date("OxA-3188 Hordeum sp.", 3200, 70)

{

Outlier ("General",0.05);

};

R_Date("OxA-3189 Vicia ervilia", 3270, 70)

{

Outlier ("General",0.05);

};

R_Date("OxA-3225 Vicia ervilia", 3160, 80)

{

Outlier ("General",0.05);

};

R_Date("OxA-10324 Hordeum sp.", 3270, 26)

{

Outlier ("General",0.05);

};

R_Date("OxA-10325 Vicia ervilia", 3228, 26)

{

Outlier ("General",0.05);

};

R_Date("OxA-10326 Vicia ervilia", 3227, 25)

{

Outlier ("General",0.05);

};

R_Date("OxA-10411 Hordeum sp.", 3150, 40)

{

Outlier ("General",0.05);

};

};

Boundary ("MPD");

};

Sequence("Mochlos (LMIB Final) Destruction")

{

Tau_Boundary (T Mochlos);

Phase ("Mochlos olive stones")

{

R_Date("Beta-85991 Olea europaea", 3240, 50)

{

Outlier ("General",0.05);

};

R_Date("Beta-85992 Olea europaea", 3180, 40)

{

Outlier ("General",0.05);

};

R_Date("Beta-115890 Olea europaea", 3170, 60)

{

Outlier ("General",0.05);

};

R_Date("Beta-129765 Olea europaea", 3220, 40)

{

Outlier ("General",0.05);

};

R_Date("Beta-151768 Olea europaea", 3270, 40)

{

Outlier ("General",0.05);

};

};

Boundary("MD");

};

};

};

Boundary("Period Between LMIB Destructions and Knossos LMII Destruction");

Sequence("Knossos LMII Destruction short-lived")

{

Tau_Boundary ("T Knossos");

Phase ("Knossos LMII Destruction, charred short-lived plant material")

{

R_Date("OxA-2096 Hordeum sp.", 3070, 70)

{

Outlier ("General",0.05);

};

R_Date("OxA-2097 Hordeum sp.", 3190, 65)

{

Outlier ("General",0.05);

};

R_Date("OxA-2098 Hordeum sp.", 3220, 65)

{

Outlier ("General",0.05);

};

R_Date("OxA-11882 Hordeum sp.", 3156, 33)

{

Outlier ("General",0.05);

};

R_Date("OxA-11943 Hordeum sp.", 3148, 23)

{

Outlier ("General",0.05);

};

};

Boundary ("KD");

};

Boundary();

};

};

**Model 1 (Fig. 9)**

Options()

{

Resolution=1;

kIterations=3000;

};

Plot()

{

Outlier_Model("General",T(5),U(0,4),"t");

Outlier_Model("SSimple",N(0,2),0,"s");

D_Sequence("Miletos WM, Quercus sp.")

{

R_Combine("RY1000-RY1010")

{

Outlier ("SSimple",0.05);

R_Date("OxA-12301", 3439, 30)

{

Outlier ("SSimple",0.05);

};

R_Date("OxA-12302", 3386, 31)

{

Outlier ("SSimple",0.05);

};

};

Gap(10);

R_Combine("RY1010-RY1020")

{

Outlier ("SSimple",0.05);

R_Date("OxA-12303", 3467, 31)

{

Outlier ("SSimple",0.05);

};

R_Date("OxA-12407", 3385, 34)

{

Outlier ("SSimple",0.05);

};

};

Gap(10);

R_Combine("RY1020-RY1030")

{

Outlier ("SSimple",0.05);

R_Date("OxA-12304", 3404, 31)

{

Outlier ("SSimple",0.05);

};

R_Date("OxA-12305", 3459, 31)

{

Outlier ("SSimple",0.05);

};

};

Gap(10);

R_Combine("RY1030-RY1040")

{

Outlier ("SSimple",0.05);

R_Date("OxA-12306", 3416, 31)

{

Outlier ("SSimple",0.05);

};

R_Date("OxA-12307", 3425, 31)

{

Outlier ("SSimple",0.05);

};

};

Gap(10);

R_Combine("RY1040-RY1050")

{

Outlier ("SSimple",0.05);

R_Date("OxA-12308", 3361, 31)

{

Outlier ("SSimple",0.05);

};

R_Date("OxA-12309", 3397, 32)

{

Outlier ("SSimple",0.05);

};

};

Gap(10);

R_Combine("RY1050-RY1060")

{

Outlier ("SSimple",0.05);

R_Date("OxA-12310", 3345, 32)

{

Outlier ("SSimple",0.05);

};

R_Date("OxA-12311", 3397, 32)

{

Outlier ("SSimple",0.05);

};

};

Gap(10);

R_Combine("RY1060-RY1070")

{

Outlier ("SSimple",0.05);

R_Date("OxA-12312", 3388, 30)

{

Outlier ("SSimple",0.05);

};

R_Date("OxA-12313", 3352, 31)

{

Outlier ("SSimple",0.05);

};

};

Gap(7);

Date("Felling Miletos WM");

//waney edge

};

Sequence("ref. [80] Olive as Sequence Only – no ‘rings’[146]")

{

Boundary("Start");

Sequence()

{

R_Date("Hd-23599-24426 'rings' 1-13", 3383, 11)

{

Outlier ("General",0.05);

};

R_Date("Hd-23587 'rings' 14-37", 3372, 12)

{

Outlier ("General",0.05);

};

R_Date("Hd-23589 'rings' 38-59", 3349, 12)

{

Outlier ("General",0.05);

};

R_Date("Hd-23588-24402 'rings' 60-72", 3331, 10)

{

Outlier ("General",0.05);

};

};

Boundary("TE5");

};

//insert Sofular Cave Sequence here – see below

Sequence()

{

Sequence("Akrotiri Stages 2/3 Stored or LCI Adv charcoal")

{

Tau_Boundary ("Stages 2/3");

Phase ("Akrotiri secure Stages 2/3 Food Products in Use/Storage or LCI Adv charcoal")

{

R_Date("OxA-1552 Lathyrus sp.", 3390, 65)

{

Outlier ("General",0.05);

};

R_Date("OxA-1555 Lathyrus sp.", 3245, 65)

{

Outlier ("General",0.05);

};

R_Date("OxA-1548 Lathyrus sp.", 3335, 60)

{

Outlier ("General",0.05);

};

R_Date("OxA-1549 Lathyrus sp.", 3460, 80)

{

Outlier ("General",0.05);

};

R_Date("OxA-1550 Lathyrus sp.", 3395, 65)

{

Outlier ("General",0.05);

};

R_Date("OxA-1553 Lathyrus sp.", 3340, 65)

{

Outlier ("General",0.05);

};

R_Date("OxA-1554 Lathyrus sp.", 3280, 65)

{

Outlier ("General",0.05);

};

R_Date("OxA-1556 Hordeum sp.", 3415, 70)

{

Outlier ("General",0.05);

};

R_Date("K-5352 pulses", 3310, 65)

{

Outlier ("General",0.05);

};

//R_Date("K-5353 pulses NOT CLEAR IF 2/3 so exclude", 3430, 90)

//{

// Outlier ("General",0.05);

//};

R_Date("K-3228 pulses", 3340, 55)

{

Outlier ("General",0.05);

};

R_Date("OxA-11817 ?Lathyrus sp.", 3348, 31)

{

Outlier ("General",0.05);

};

R_Date("OxA-11818 Hordeum sp.", 3367, 33)

{

Outlier ("General",0.05);

};

R_Date("OxA-11820 Hordeum sp.", 3400, 31)

{

Outlier ("General",0.05);

};

R_Date("OxA-11869 Hordeum sp.", 3336, 34)

{

Outlier ("General",0.05);

};

R_Date("OxA-12170 ?Lathyrus sp.", 3336, 28)

{

Outlier ("General",0.05);

};

R_Date("OxA-12171 Hordeum sp.", 3372, 28)

{

Outlier ("General",0.05);

};

R_Date("OxA-12175 Hordeum sp.", 3318, 28)

{

Outlier ("General",0.05);

};

R_Date("OxA-12172 Hordeum sp.", 3321, 32)

{

Outlier ("General",0.05);

};

R_Date("VERA-2756 Hordeum sp.", 3317, 28)

{

Outlier ("General",0.05);

};

R_Date("VERA-2757 ?Lathyrus sp.", 3315, 31)

{

Outlier ("General",0.05);

};

R_Date("VERA-2758 Hordeum sp.", 3339, 28)

{

Outlier ("General",0.05);

};

R_Date("VERA-2757 repeat ?Lathyrus sp.", 3390, 32)

{

Outlier ("General",0.05);

};

R_Date("VERA-2758 repeat Hordeum sp.", 3322, 32)

{

Outlier ("General",0.05);

};

R_Date ("OxA-25176 insect chitin", 3368, 29)

{

Outlier ("General",0.05);

};

Label("Hd data on SL samples from Akrotiri VDL");

R_Date("Hd-7092-6795 peas",3360,60)

{

Outlier ("General",0.05);

};

// R_Date("Hd-6058-5519 grains no context so exclude",3490,80)

//{

// Outlier ("General",0.05);

// };

// R_Date("Hd-6059-7967 grains no context so exclude",3140,70)

// {

// Outlier ("General",0.05);

// };

Label("LCI Advanced or Advanced? or VDL charcoal, Akrotiri, Thera");

R_Date ("DEM-1311 LC I VDL", 3307, 25)

{

Outlier ("General",0.05);

};

R_Date ("DEM-1529 LC I Advanced? VDL", 3281,25)

{

Outlier ("General",0.05);

};

R_Date ("DEM-1607 LC I Advanced VDL", 3228,30)

{

Outlier ("General",0.05);

};

R_Date ("DEM-1624 LC I Advanced VDL", 3360,25)

{

Outlier ("General",0.05);

};

R_Date ("DEM-1615 LC I Advanced? VDL", 3389,25)

{

Outlier ("General",0.05);

};

R_Date( "K-4255 twig in pumice so assume LCI Advanced", 3380, 60)

{

Outlier ("General",0.05);

};

};

Boundary("E2/3");

};

Interval("Stage 3 to eruption");

Sequence("Santorini Eruption/Tsunami no Santorini Data and olive in pumice dates")

{

Tau_Boundary ("TnoS+dataset iii");

Phase ("Close TPQs for VDL or even date of VDL")

{

Label("LBIA/LMIA mature and destruction later below Thera tephra, Trianda, Rhodes, so TPQ");

R_Date ("DEM-94 charcoal", 3347,46)

{

Outlier ("General",0.05);

};

R_Date ("DEM-93 charcoal", 3358,48)

{

Outlier ("General",0.05);

};

R_Combine("Trianda short-lived late LMIA twig, pre-Thera-tephra, Quercus sp.")

{

Outlier ("General",0.05);

R_Date("OxA-10643", 3367, 39)

{

Outlier("SSimple",0.05);

};

R_Date("OxA-11884", 3344, 32)

{

Outlier("SSimple",0.05);

};

};

Date("=Felling Miletos WM");

Label("Thera Tsunami Palaikastro");

Label("Palaikastro Promontory");

R_Date("GrA-30336 Cattle bone",3310,35)

{

Outlier ("General",0.05);

};

R_Date("GrA-30339 Cattle bone",3390,35)

{

Outlier ("General",0.05);

};

Label("Bone/jaw, tooth Palaikastro drain with stratified Thera tephra");

R_Date("GrA-28991 goat/sheep bone/jaw",3325,40)

{

Outlier ("General",0.05);

};

R_Date("GrA-29041 goat/sheep bone/jaw",3345,40)

{

Outlier ("General",0.05);

};

R_Date("GrA-29042 tooth",3385,40)

{

Outlier ("General",0.05);

};

Label("Thera Tsunami Çeşme-Bağlararası");

R_Date("OxA-38858 H1a S",3275,17)

{

Outlier ("General",0.05);

};

R_Date("OxA-38881 H1a B",3367,22)

{

Outlier ("General",0.05);

};

R_Date("OxA-38973 H1a C",3318,19)

{

Outlier ("General",0.05);

};

R_Date("OxA-38972 H1a B",3316,20)

{

Outlier ("General",0.05);

};

R_Date("OxA-38857 H1a S",3312,17)

{

Outlier ("General",0.05);

};

R_Date("OxA-38950 H1b C",3384,22)

{

Outlier ("General",0.05);

};

R_Date("D-AMS019172 H1b B",3372,27)

{

Outlier ("General",0.05);

};

R_Date("OxA-38966 H1c C",3297,19)

{

Outlier ("General",0.05);

};

R_Date("D-AMS019173 H1c C",3291,30)

{

Outlier ("General",0.05);

};

Label(“Thera Tsunami Eşençay Delta”);

R_Date("Lyon7920 organic-rich layer",3295,30)

{

Outlier ("General",0.05);

};

Label("Olive branch or tree root samples from Santorini pre-eruption ABA and HS TPQ for eruption");

R_Date("VERA-5614 ABA olive branch",3282,21)

{

Outlier ("General",0.05);

};

R_Date("VERA-5614HS olive branch",3359,33)

{

Outlier ("General",0.05);

};

R_Date("VERA-5615 ABA olive branch", 3280,24)

{

Outlier ("General",0.05);

};

R_Date("VERA-5615HS olive branch", 3321,24)

{

Outlier ("General",0.05);

};

R_Date("VERA-5620 ABA olive branch",3277,25)

{

Outlier ("General",0.05);

};

R_Date("VERA-5620HS olive branch",3345,24)

{

Outlier ("General",0.05);

};

R_Date("VERA-5610 ABA olive branch",3399,25)

{

Outlier ("General",0.05);

};

R_Date("VERA-5610HS olive branch",3342,26)

{

Outlier ("General",0.05);

};

R_Date("VERA-5083 ABA olive branch",3270,36)

{

Outlier ("General",0.05);

};

R_Date("VERA-5083HS olive branch",3326,77)

{

Outlier ("General",0.05);

};

R_Date("VERA-5082 ABA olive branch",3332,38)

{

Outlier ("General",0.05);

};

R_Date("VERA-5082HS olive branch",3369,36)

{

Outlier ("General",0.05);

};

R_Date("VERA-5084 ABA root",3354,32)

{

Outlier ("General",0.05);

};

R_Date("VERA-5084HS root",3368,34)

{

Outlier ("General",0.05);

};

};

Boundary("=TE5");

};

Before("Kolonna Phase L TAQ")

{

Date("Vera-4630 Prior dataset i",Prior("VERA_4630_B","VERA_4630_B.prior"););

};

};

Difference("D","TE5","E2/3",LnN(ln(3),ln(2)));

};

Note: TE5 = Thera Eruption Boundary (stage v) with LnN(ln(3),ln(2))) constraint applied to the Difference query.

Note: the alternative models reported in Tables 1-2 with the uniform 0-15 years constraint applied to the Difference query are the same except for the line of code:

Difference("D","TE5","E2/3",U(0,15));

Note: The Model 1 runfile uses a Prior command. This calls a probability distribution function previously defined—in this case the modelled posterior probability for the date VERA-4630 from the run of dataset (i). In order for the model to run, this Prior file must have been saved beforehand, and named as “VERA_4630_B.prior”, either by running the dataset (i) runfile above and saving the probability distribution file for VERA-4630, or by copying the file listing below and saving this as a Prior file named “VERA_4630_B.prior”:

-1733.5 0

-1732.5 2.8558e-7

-1731.5 5.814e-7

-1730.5 7.242e-7

-1729.5 0.0000021725

-1728.5 0.000003621

-1727.5 0.000006089

-1726.5 0.000005793

-1725.5 0.000005946

-1724.5 0.000011301

-1723.5 0.000013769

-1722.5 0.000013769

-1721.5 0.000012464

-1720.5 0.000014208

-1719.5 0.000020725

-1718.5 0.000017828

-1717.5 0.000024346

-1716.5 0.000021164

-1715.5 0.000022459

-1714.5 0.000018695

-1713.5 0.000015942

-1712.5 0.000016666

-1711.5 0.00001739

-1710.5 0.000014208

-1709.5 0.000011301

-1708.5 0.000012749

-1707.5 0.00000971

-1706.5 0.0000087

-1705.5 0.000007823

-1704.5 0.00000768

-1703.5 0.000009424

-1702.5 0.000007394

-1701.5 0.000006089

-1700.5 0.000006375

-1699.5 0.000007976

-1698.5 0.000008119

-1697.5 0.000011015

-1696.5 0.000008119

-1695.5 0.000012321

-1694.5 0.00000971

-1693.5 0.000013045

-1692.5 0.000015656

-1691.5 0.000017104

-1690.5 0.000025366

-1689.5 0.000026375

-1688.5 0.000023336

-1687.5 0.00003188

-1686.5 0.00003566

-1685.5 0.0000442

-1684.5 0.00004334

-1683.5 0.0000558

-1682.5 0.00005695

-1681.5 0.00006927

-1680.5 0.00007174

-1679.5 0.00007783

-1678.5 0.00009884

-1677.5 0.00010624

-1676.5 0.00011812

-1675.5 0.00012986

-1674.5 0.00013782

-1673.5 0.00015392

-1672.5 0.00017464

-1671.5 0.00019334

-1670.5 0.00021479

-1669.5 0.00023913

-1668.5 0.00025043

-1667.5 0.00029246

-1666.5 0.0003186

-1665.5 0.0003488

-1664.5 0.0004

-1663.5 0.0004259

-1662.5 0.000462

-1661.5 0.000529

-1660.5 0.0005746

-1659.5 0.0005951

-1658.5 0.0006142

-1657.5 0.0006436

-1656.5 0.0006657

-1655.5 0.0007058

-1654.5 0.0007342

-1653.5 0.0007649

-1652.5 0.0007542

-1651.5 0.0007503

-1650.5 0.0007377

-1649.5 0.0006738

-1648.5 0.0006748

-1647.5 0.0006639

-1646.5 0.00063

-1645.5 0.000682

-1644.5 0.0007201

-1643.5 0.0007949

-1642.5 0.0008565

-1641.5 0.0009323

-1640.5 0.0010452

-1639.5 0.0010871

-1638.5 0.0010959

-1637.5 0.0011359

-1636.5 0.0012219

-1635.5 0.0012168

-1634.5 0.0012564

-1633.5 0.0013129

-1632.5 0.0013948

-1631.5 0.0014355

-1630.5 0.0015126

-1629.5 0.0015971

-1628.5 0.0016229

-1627.5 0.0017483

-1626.5 0.0018429

-1625.5 0.0019465

-1624.5 0.0021291

-1623.5 0.0023113

-1622.5 0.0025828

-1621.5 0.0028267

-1620.5 0.0030307

-1619.5 0.003402

-1618.5 0.003616

-1617.5 0.003873

-1616.5 0.004096

-1615.5 0.004335

-1614.5 0.004668

-1613.5 0.004981

-1612.5 0.00529

-1611.5 0.005491

-1610.5 0.005711

-1609.5 0.006021

-1608.5 0.006117

-1607.5 0.006335

-1606.5 0.006388

-1605.5 0.006602

-1604.5 0.006669

-1603.5 0.006714

-1602.5 0.006857

-1601.5 0.006952

-1600.5 0.00711

-1599.5 0.00709

-1598.5 0.007146

-1597.5 0.007162

-1596.5 0.007238

-1595.5 0.007359

-1594.5 0.007256

-1593.5 0.00734

-1592.5 0.007428

-1591.5 0.007534

-1590.5 0.007589

-1589.5 0.007592

-1588.5 0.007704

-1587.5 0.007734

-1586.5 0.00783

-1585.5 0.007784

-1584.5 0.007864

-1583.5 0.007912

-1582.5 0.007955

-1581.5 0.008065

-1580.5 0.008072

-1579.5 0.008034

-1578.5 0.008165

-1577.5 0.008139

-1576.5 0.008228

-1575.5 0.008275

-1574.5 0.008317

-1573.5 0.008349

-1572.5 0.008323

-1571.5 0.008306

-1570.5 0.008312

-1569.5 0.008348

-1568.5 0.008468

-1567.5 0.008529

-1566.5 0.008548

-1565.5 0.008657

-1564.5 0.008873

-1563.5 0.008887

-1562.5 0.008966

-1561.5 0.00904

-1560.5 0.00919

-1559.5 0.009209

-1558.5 0.009269

-1557.5 0.009425

-1556.5 0.009394

-1555.5 0.009515

-1554.5 0.009589

-1553.5 0.009619

-1552.5 0.009566

-1551.5 0.009594

-1550.5 0.00961

-1549.5 0.009618

-1548.5 0.00966

-1547.5 0.009729

-1546.5 0.00975

-1545.5 0.009916

-1544.5 0.009989

-1543.5 0.009979

-1542.5 0.010133

-1541.5 0.010115

-1540.5 0.010199

-1539.5 0.01006

-1538.5 0.010022

-1537.5 0.009933

-1536.5 0.009834

-1535.5 0.009809

-1534.5 0.009821

-1533.5 0.0096

-1532.5 0.009373

-1531.5 0.008959

-1530.5 0.008477

-1529.5 0.007834

-1528.5 0.007172

-1527.5 0.006667

-1526.5 0.006602

-1525.5 0.00667

-1524.5 0.007024

-1523.5 0.007527

-1522.5 0.007976

-1521.5 0.008124

-1520.5 0.008214

-1519.5 0.007961

-1518.5 0.00779

-1517.5 0.007394

-1516.5 0.006864

-1515.5 0.006407

-1514.5 0.006171

-1513.5 0.005945

-1512.5 0.005786

-1511.5 0.005835

-1510.5 0.00575

-1509.5 0.005807

-1508.5 0.005555

-1507.5 0.005301

-1506.5 0.004817

-1505.5 0.004411

-1504.5 0.003981

-1503.5 0.003382

-1502.5 0.0030644

-1501.5 0.0026529

-1500.5 0.0022649

-1499.5 0.0019507

-1498.5 0.0016887

-1497.5 0.0014901

-1496.5 0.0012641

-1495.5 0.0010878

-1494.5 0.00094

-1493.5 0.0007926

-1492.5 0.0006833

-1491.5 0.0005854

-1490.5 0.0005291

-1489.5 0.0004551

-1488.5 0.0004213

-1487.5 0.0004003

-1486.5 0.000379

-1485.5 0.0003694

-1484.5 0.0003709

-1483.5 0.0003649

-1482.5 0.0003951

-1481.5 0.0004478

-1480.5 0.0004659

-1479.5 0.0004981

-1478.5 0.0005281

-1477.5 0.0005945

-1476.5 0.0006444

-1475.5 0.0006993

-1474.5 0.0007716

-1473.5 0.0008517

-1472.5 0.0008696

-1471.5 0.0009325

-1470.5 0.0009254

-1469.5 0.0009829

-1468.5 0.0009704

-1467.5 0.0009742

-1466.5 0.0009788

-1465.5 0.0009659

-1464.5 0.0009193

-1463.5 0.0008952

-1462.5 0.000892

-1461.5 0.000831

-1460.5 0.0008041

-1459.5 0.0007429

-1458.5 0.0007487

-1457.5 0.0006652

-1456.5 0.0006446

-1455.5 0.0005842

-1454.5 0.0005222

-1453.5 0.0004888

-1452.5 0.0004619

-1451.5 0.0003961

-1450.5 0.0003762

-1449.5 0.0003286

-1448.5 0.00031145

-1447.5 0.00028217

-1446.5 0.00026232

-1445.5 0.00023565

-1444.5 0.00022551

-1443.5 0.00019594

-1442.5 0.00018044

-1441.5 0.00017058

-1440.5 0.00015609

-1439.5 0.00014333

-1438.5 0.00012073

-1437.5 0.0001145

-1436.5 0.00010421

-1435.5 0.00009536

-1434.5 0.00008116

-1433.5 0.00007681

-1432.5 0.00007261

-1431.5 0.00006334

-1430.5 0.00005492

-1429.5 0.00005435

-1428.5 0.00005029

-1427.5 0.00004985

-1426.5 0.00004826

-1425.5 0.00003855

-1424.5 0.00004116

-1423.5 0.00003246

-1422.5 0.000029568

-1421.5 0.000027966

-1420.5 0.000024489

-1419.5 0.000020286

-1418.5 0.000022612

-1417.5 0.000017104

-1416.5 0.000016084

-1415.5 0.000013473

-1414.5 0.000012464

-1413.5 0.000008843

-1412.5 0.000009128

-1411.5 0.000011454

-1410.5 0.000007537

-1409.5 0.000011158

-1408.5 0.000007976

-1407.5 0.000007242

-1406.5 0.000007823

-1405.5 0.00000565

-1404.5 0.000007099

-1403.5 0.000006517

-1402.5 0.000006956

-1401.5 0.000008261

-1400.5 0.00000565

-1399.5 0.000004783

-1398.5 0.000004488

-1397.5 0.000005365

-1396.5 0.000004641

-1395.5 0.0000027538

-1394.5 0.000004783

-1393.5 0.000004926

-1392.5 0.000003478

-1391.5 0.000004059

-1390.5 0.00000565

-1389.5 0.000004202

-1388.5 0.000005365

-1387.5 0.0000023152

-1386.5 0.0000023152

-1385.5 0.000003478

-1384.5 0.000004926

-1383.5 0.000003917

-1382.5 0.0000023152

-1381.5 0.0000023152

-1380.5 0.0000021725

-1379.5 0.000005365

-1378.5 0.0000028966

-1377.5 0.000004783

-1376.5 0.0000021725

-1375.5 0.000003335

-1374.5 0.0000024682

-1373.5 0.0000018869

-1372.5 0.000002611

-1371.5 0.0000023152

-1370.5 0.0000017441

-1369.5 0.0000015911

-1368.5 0.0000018869

-1367.5 0.0000024682

-1366.5 0.0000011627

-1365.5 0.000003764

-1364.5 0.0000013055

-1363.5 7.242e-7

-1362.5 0.0000014483

-1361.5 0.0000018869

-1360.5 0.0000018869

-1359.5 0.0000014483

-1358.5 0.0000010097

-1357.5 0.0000017441

-1356.5 0.0000020297

-1355.5 0.0000015911

-1354.5 0.0000018869

-1353.5 0.0000021725

-1352.5 0.0000011627

-1351.5 0.0000017441

-1350.5 0.0000013055

-1349.5 8.669e-7

-1348.5 0.0000013055

-1347.5 8.669e-7

-1346.5 0.0000013055

-1345.5 4.386e-7

-1344.5 0.0000021725

-1343.5 0.0000014483

-1342.5 0.0000027538

-1341.5 0.0000013055

-1340.5 8.669e-7

-1339.5 0.0000010097

-1338.5 0.0000015911

-1337.5 0.0000011627

-1336.5 2.8558e-7

-1335.5 5.814e-7

-1334.5 1.4279e-7

-1333.5 7.242e-7

-1332.5 2.8558e-7

-1331.5 7.242e-7

-1330.5 7.242e-7

-1329.5 8.669e-7

-1328.5 2.8558e-7

-1327.5 2.8558e-7

-1326.5 1.4279e-7

-1325.5 8.669e-7

-1324.5 1.4279e-7

-1323.5 5.814e-7

-1322.5 4.386e-7

-1321.5 2.8558e-7

-1320.5 5.814e-7

-1319.5 2.8558e-7

-1318.5 8.669e-7

-1317.5 4.386e-7

-1316.5 2.8558e-7

-1315.5 0.0000011627

-1314.5 2.8558e-7

-1313.5 2.8558e-7

-1312.5 0

-1311.5 5.814e-7

-1310.5 7.242e-7

-1309.5 7.242e-7

-1308.5 0.0000011627

-1307.5 1.4279e-7

-1306.5 1.4279e-7

-1305.5 4.386e-7

-1304.5 0

-1303.5 2.8558e-7

-1302.5 2.8558e-7

-1301.5 2.8558e-7

-1300.5 0

-1299.5 0

-1298.5 1.4279e-7

-1297.5 0

-1296.5 2.8558e-7

-1295.5 0

-1294.5 1.4279e-7

-1293.5 0

-1292.5 1.4279e-7

-1291.5 0

-1290.5 0

-1289.5 0

**Model 2 (Fig. 12)**

The version with the GSRO test adds at the start (e.g. above Outlier_Model lines) the line: Delta_R(“Aegean Max Test”,4,2);

Options()

{

Resolution=1;

kIterations=300;

};

Plot()

{

Outlier_Model("General",T(5),U(0,4),"t");

Outlier_Model("SSimple",N(0,2),0,"s");

Outlier_Model("Charcoal",Exp(1,-10,0),U(0,3),"t");

D_Sequence("Miletos WM, Quercus sp.")

{

R_Combine("RY1000-RY1010")

{

Outlier ("SSimple",0.05);

R_Date("OxA-12301", 3439, 30)

{

Outlier ("SSimple",0.05);

};

R_Date("OxA-12302", 3386, 31)

{

Outlier ("SSimple",0.05);

};

};

Gap(10);

R_Combine("RY1010-RY1020")

{

Outlier ("SSimple",0.05);

R_Date("OxA-12303", 3467, 31)

{

Outlier ("SSimple",0.05);

};

R_Date("OxA-12407", 3385, 34)

{

Outlier ("SSimple",0.05);

};

};

Gap(10);

R_Combine("RY1020-RY1030")

{

Outlier ("SSimple",0.05);

R_Date("OxA-12304", 3404, 31)

{

Outlier ("SSimple",0.05);

};

R_Date("OxA-12305", 3459, 31)

{

Outlier ("SSimple",0.05);

};

};

Gap(10);

R_Combine("RY1030-RY1040")

{

Outlier ("SSimple",0.05);

R_Date("OxA-12306", 3416, 31)

{

Outlier ("SSimple",0.05);

};

R_Date("OxA-12307", 3425, 31)

{

Outlier ("SSimple",0.05);

};

};

Gap(10);

R_Combine("RY1040-RY1050")

{

Outlier ("SSimple",0.05);

R_Date("OxA-12308", 3361, 31)

{

Outlier ("SSimple",0.05);

};

R_Date("OxA-12309", 3397, 32)

{

Outlier ("SSimple",0.05);

};

};

Gap(10);

R_Combine("RY1050-RY1060")

{

Outlier ("SSimple",0.05);

R_Date("OxA-12310", 3345, 32)

{

Outlier ("SSimple",0.05);

};

R_Date("OxA-12311", 3397, 32)

{

Outlier ("SSimple",0.05);

};

};

Gap(10);

R_Combine("RY1060-RY1070")

{

Outlier ("SSimple",0.05);

R_Date("OxA-12312", 3388, 30)

{

Outlier ("SSimple",0.05);

};

R_Date("OxA-12313", 3352, 31)

{

Outlier ("SSimple",0.05);

};

};

Gap(7);

Date("Felling Miletos WM");

//waney edge

};

Sequence("ref. [80] with Olive as Sequence Only - no ‘rings’")

{

Boundary("Start Olive");

Sequence()

{

R_Date("Hd-23599-24426 'rings' 1-13", 3383, 11)

{

Outlier ("General",0.05);

};

R_Date("Hd-23587 'rings' 14-37", 3372, 12)

{

Outlier ("General",0.05);

};

R_Date("Hd-23589 'rings' 38-59", 3349, 12)

{

Outlier ("General",0.05);

};

R_Date("Hd-23588-24402 'rings' 60-72", 3331, 10)

{

Outlier ("General",0.05);

};

};

Boundary("TE5");

};

Sequence()

{

Tau_Boundary("TM4N003 Olive");

Phase("M4N003 Olea europaea")

{

//The age differences suggest not same annual 'rings' sampled; olive 'rings' also not secure basis for fixed Sequence. Hence Tau_Boundary & Boundary model to bark/use

R_Date("OxA-10319 Inner 'rings' 3-4", 3424, 38)

{

Outlier ("General",0.05);

};

R_Date("VERA-2747 Inner 'rings' 3-4", 3386, 30)

{

Outlier ("General",0.05);

};

R_Date("OxA-10316 'rings' 3-5", 3342, 38)

{

Outlier ("General",0.05);

};

R_Date("VERA-2744 'rings' 3-5", 3427, 31)

{

Outlier ("General",0.05);

};

R_Date("OxA-10318 'rings' 5-6", 3355, 40)

{

Outlier ("General",0.05);

};

R_Date("VERA-2746 'rings' 5-6", 3471, 28)

{

Outlier ("General",0.05);

};

R_Date("OxA-10315 'rings' 6-8bark", 3446, 39)

{

Outlier ("General",0.05);

};

R_Date("VERA-2743 'rings' 6-8bark", 3413, 28)

{

Outlier ("General",0.05);

};

R_Date("OxA-10317 Outermost 'rings' 7-8bark", 3440, 35)

{

Outlier ("General",0.05);

};

R_Date("VERA-2745 Outermost 'rings' 7-8bark", 3386, 28)

{

Outlier ("General",0.05);

};

};

Boundary("Use TM4N003 Olive");

};

D_Sequence("65/N001/I2 Tamarix sp.")

{

R_Combine("Ring 1")

{

Outlier ("SSimple",0.05);

R_Date("OxA-10314", 3330, 27)

{

Outlier ("SSimple",0.05);

};

R_Date("VERA-2751", 3325, 28)

{

Outlier ("SSimple",0.05);

};

};

R_Combine("Ring 2")

{

Outlier ("SSimple",0.05);

R_Date("OxA-10313", 3353, 27)

{

Outlier ("SSimple",0.05);

};

R_Date("VERA-2749", 3335, 33)

{

Outlier ("SSimple",0.05);

};

};

R_Combine("65/N001/I2 Ring 3 Bark")

{

Outlier ("SSimple",0.05);

R_Date("OxA-10312", 3293, 27)

{

Outlier ("SSimple",0.05);

};

R_Date("VERA-2748", 3319, 28)

{

Outlier ("SSimple",0.05);

};

};

};

D_Sequence("Trianda WM, Quercus sp. late MBA/Early LMIA")

{

R_Combine("RY1pith-RY10 @5.5")

{

Outlier ("SSimple",0.05);

R_Date("OxA-10730", 3490, 45)

{

Outlier ("SSimple",0.05);

};

R_Date("OxA-11948", 3526, 25)

{

Outlier ("SSimple",0.05);

};

R_Date("VERA-2742", 3476, 28)

{

Outlier ("SSimple",0.05);

};

};

Gap( 10);

R_Combine("RY11-RY20 @15.5")

{

Outlier ("SSimple",0.05);

R_Date("OxA-10729", 3410, 45)

{

Outlier ("SSimple",0.05);

};

R_Date("OxA-11946", 3474, 24)

{

Outlier ("SSimple",0.05);

};

R_Date("VERA-2741", 3485, 28)

{

Outlier ("SSimple",0.05);

};

};

Gap( 10);

R_Combine("RY21-RY30bark @25.5")

{

Outlier ("SSimple",0.05);

R_Date("OxA-10728", 3455, 45)

{

Outlier ("SSimple",0.05);

};

R_Date("OxA-11945", 3473, 24)

{

Outlier ("SSimple",0.05);

};

R_Date("VERA-2740", 3481, 32)

{

Outlier ("SSimple",0.05);

};

};

Gap(4.5);

Date("Felling Trianda WM");

//bark/waney edge

};

Sequence("Kolonna, Aegina")

{

//S = short-lived plant material, B = animal bone, C = charcoal (apparently not very long-lived, T = wood twig, so shorter-lived).

//ref. [89] data and model

Boundary("Begin E");

Phase("Phase E")

{

Sequence()

{

Combine("fire destruction")

{

Outlier ("General",0.05);

R_Date("VERA-2678 S", 3724, 35)

{

Outlier("SSimple",0.05);

};

R_Date("VERA-2680 S", 3722, 35)

{

Outlier("SSimple",0.05);

};

R_Date("VERA-2681 S", 3739, 35)

{

Outlier("SSimple",0.05);

};

R_Date("VERA-2679 S", 3761, 35)

{

Outlier("SSimple",0.05);

};

R_Date("VRI-0395 C", 3670, 90)

{

Outlier("SSimple",0.05);

};

R_Date("HV-5841 C", 3625, 65)

{

Outlier("SSimple",0.05);

};

R_Date("VERA-2682 S", 3712, 35)

{

Outlier("SSimple",0.05);

};

R_Date("VERA-2683 S", 3721, 35)

{

Outlier("SSimple",0.05);

};

R_Date("HV-5840 C", 3820, 65)

{

Outlier("SSimple",0.05);

};

};

R_Date("VERA-4641 B", 3759, 35)

{

Outlier ("General",0.05);

};

};

R_Date("VERA-2688 B", 3698, 33)

{

Outlier ("General",0.05);

};

};

Boundary("Transition E/F");

Phase("Phase F")

{

R_Date("VERA-2692 B", 3704, 36)

{

Outlier ("General",0.05);

};

};

Boundary("Transition F/G");

Phase("Phase G")

{

Sequence("Sequence")

{

R_Date("VERA-4640 B", 3800, 44)

{

Outlier ("General",0.05);

};

R_Date("VERA-4639 B", 3809, 32)

{

Outlier ("General",0.05);

};

};

R_Date("VERA-4638 B", 3646, 32)

{

Outlier ("General",0.05);

};

R_Date("VERA-4281 S", 3740, 36)

{

Outlier ("General",0.05);

};

R_Date("VERA-4282 S", 3711, 34)

{

Outlier ("General",0.05);

};

R_Date("VERA-4283 S", 3780, 37)

{

Outlier ("General",0.05);

};

};

Boundary("Transition G/H");

Phase("Phase H")

{

Sequence("Sequence")

{

R_Date("VERA-4637 B", 3643, 30)

{

Outlier ("General",0.05);

};

R_Date("VERA-4636 B", 3628, 30)

{

Outlier ("General",0.05);

};

};

Sequence("Sequence")

{

R_Date("VERA-4280 S", 3724, 39)

{

Outlier ("General",0.05);

};

R_Date("VERA-4279 S", 3718, 38)

{

Outlier ("General",0.05);

};

};

R_Date("VERA-2687", 3694 B, 35)

{

Outlier ("General",0.05);

};

};

Boundary("Transition H/I");

Phase("Phase I")

{

Sequence()

{

Phase("before Minoan layer")

{

R_Date("VERA-4634 B", 3544, 37)

{

Outlier ("General",0.05);

};

R_Date("VERA-4278 S", 3522, 38)

{

Outlier ("General",0.05);

};

};

Combine("Minoan layer")

{

Outlier ("General",0.05);

R_Date("VERA-4038 S", 3506, 34)

{

Outlier("SSimple",0.05);

};

R_Date("VERA-4576 B", 3482, 37)

{

Outlier("SSimple",0.05);

};

R_Date("VERA-4575 B", 3537, 36)

{

Outlier("SSimple",0.05);

};

R_Date("VERA-4578 B", 3501, 39)

{

Outlier("SSimple",0.05);

};

R_Date("VERA-4579 B", 3526, 38)

{

Outlier("SSimple",0.05);

};

R_Date("VERA-4580 B", 3506, 33)

{

Outlier("SSimple",0.05);

};

R_Date("VERA-4276 S", 3506, 37)

{

Outlier("SSimple",0.05);

};

R_Date("VERA-4275 S", 3544, 38)

{

Outlier("SSimple",0.05);

};

};

R_Date("VERA-4577 B", 3458, 39)

{

Outlier ("General",0.05);

};

};

};

Boundary("Transition I/J");

Phase("Phase J = MHIII")

{

Sequence()

{

Combine()

{

Outlier ("General",0.05);

R_Date("VERA-4571 B", 3469, 38)

{

Outlier("SSimple",0.05);

};

R_Date("VERA-4574 B", 3430, 39)

{

Outlier("SSimple",0.05);

};

R_Date("VERA-4573 B", 3485, 36)

{

Outlier("SSimple",0.05);

};

};

Phase()

{

R_Date("VERA-4572 B", 3407, 38)

{

Outlier ("General",0.05);

};

R_Date("VERA-4570 B", 3428, 36)

{

Outlier ("General",0.05);

};

};

};

};

Boundary("Kolonna Transition J/K");

Phase("Phase K - LHI")

{

Sequence()

{

R_Date("VERA-4633 B", 3333, 29)

{

Outlier ("General",0.05);

};

R_Date("VERA-4632 B", 3356, 36)

{

Outlier ("General",0.05);

};

R_Date("VERA-4631 B", 3349, 36)

{

Outlier ("General",0.05);

};

};

};

Boundary("Kolonna Transition K/L");

Phase("Phase L - LHII")

{

R_Date("VERA-4630 B", 3313, 48)

{

Outlier ("General",0.05);

};

Date("Kolonna LHII");

};

Boundary("End Kolonna L: End LHII");

Boundary("Begin Kolonna M: LHIIIA");

Phase("Phase M - LHIIIA")

{

Combine()

{

Outlier ("General",0.05);

R_Date("VERA-4284 S", 3044, 35)

{

Outlier("SSimple",0.05);

};

R_Date("VERA-4582 T", 2986, 33)

{

Outlier("SSimple",0.05);

};

R_Date("VERA-4285 S", 3040, 37)

{

Outlier("SSimple",0.05);

};

};

};

Boundary("End Phase M");

};

Sequence("Kommos early LMIA TPQs to Date Early LMIA")

{

Boundary("Start Kommos Early LMIA charcoal TPQ Early LMIA");

Phase( "Kommos early LMIA secure charcoal longer-lived, TPQs")

{

R_Combine("K85A/62D/9:92 Quercus sp.")

{

Outlier ("General",0.05);

R_Date("OxA-11251", 3505, 40)

{

Outlier("SSimple",0.05);

};

R_Date("VERA-2636", 3445, 25)

{

Outlier("SSimple",0.05);

};

};

R_Combine ("K85A/62D/8:83 Quercus sp.")

{

Outlier ("General",0.05);

R_Date( "OxA-11253", 3397, 38)

{

Outlier("SSimple",0.05);

};

//R_Date("VERA-2638", 3600, 19)

//{

// Outlier("SSimple",0.05);

//};

//Same sample but X2 22.484 > 3.8 - given other data assume VERA-2638 must be too old outlier and exclude

};

R_Combine("Space 25B, Tr.66B likely Cupressaceae")

{

Outlier ("General",0.05);

R_Date("OxA-11883", 3485, 33)

{

Outlier("SSimple",0.05);

};

R_Date("OxA-11944", 3435, 25)

{

Outlier("SSimple",0.05);

};

R_Date("OxA-3429", 3350, 70)

{

Outlier("SSimple",0.05);

};

};

};

Boundary("longer-lived samples to shorter-lived twig and likely age Early LMIA");

R_Combine("Kommos Early LMIA charred twig")

{

Outlier ("General",0.05);

R_Date("OxA-11252 K85A/66B/4:22+23 twig", 3375, 45)

{

Outlier("SSimple",0.05);

};

R_Date("VERA-2637 K85A/66B/4:22+23 twig", 3390, 20)

{

Outlier("SSimple",0.05);

};

};

Boundary("End early LMIA Kommos data");

};

Page();

Sequence("Early LCI/LMIA to LMII")

{

Boundary("Start");

After("Late MBA/Early LMIA TPQ or Transition MH to LHI")

{

Date("=Felling Trianda WM");

Date("=Kolonna Transition J/K");

};

Phase("Late Middle Cycladic (LMC)/Early LCI/LMIA Akrotiri or Earlier LMIA Trianda")

{

Sequence()

{

Boundary();

Phase("Akrotiri LMC/Earlier LCI")

{

R_Combine("M54/2/VII/60/de>247")

{

Outlier("Charcoal",1);

R_Date("OxA-11250 charcoal", 3550, 45)

{

Outlier ("SSimple",0.05);

};

R_Date("Hd22037 charcoal", 3552, 19)

{

Outlier ("SSimple",0.05);

};

};

R_Date("DEM-1458 charcoal",3375,25)

{

Outlier("Charcoal",1);

};

R_Date("DEM-1528 charcoal before LC?", 3462, 25)

{

Outlier("Charcoal",1);

};

R_Date("DEM-1531 charcoal LMC", 3441, 25)

{

Outlier("Charcoal",1);

};

R_Date("DEM-1623 charcoal LMC", 3499, 25)

{

Outlier("Charcoal",1);

};

};

Boundary();

};

Sequence()

{

Boundary();

Phase("Trianda Earlier LMIA")

{

R_Date("DEM-89 charcoal",3517,83)

{

Outlier ("SSimple",0.05);

};

R_Date("DEM-859 charcoal",3568,44)

{

Outlier ("SSimple",0.05);

};

};

Boundary();

};

Date("=Kommos Early LMIA charred twig");

};

Boundary("Early LMC/LCI to LCI Transition");

Phase("LCI Akrotiri pre final VDL LCI Charcoal and SL Stages 1/2 or LCI or LMIA Trianda")

{

Sequence()

{

Boundary();

Phase("Akrotiri LCI to pre-stages 2/3")

{

Date("=Use TM4N003 Olive");

Date("=65/N001/I2 Ring 3 Bark");

R_Date ("DEM-1313 LC I", 3396, 25)

{

Outlier("Charcoal",1);

};

R_Date ("DEM-1314 LC I", 3467, 25)

{

Outlier("Charcoal",1);

};

R_Date ("DEM-1345 LC I", 3441, 25)

{

Outlier("Charcoal",1);

};

R_Date ("DEM-1455 LC I", 3508, 30)

{

Outlier("Charcoal",1);

};

R_Date ("DEM-1456 LC I", 3456, 25)

{

Outlier("Charcoal",1);

};

R_Date ("DEM-1457 LC I", 3436, 25)

{

Outlier("Charcoal",1);

};

R_Date ("DEM-1609 LC I", 3433, 25)

{

Outlier("Charcoal",1);

};

R_Date ("DEM-1610 LC I", 3420, 25)

{

Outlier("Charcoal",1);

};

R_Date ("DEM-1646 LC I", 3508, 25)

{

Outlier("Charcoal",1);

};

R_Date("OxA-1558 legumes",3400,60)

{

Outlier ("General",0.05);

};

R_Date("OxA-1551 Hordeum sp.",3465,65)

{

Outlier ("General",0.05);

};

R_Date("OxA-1557 legumes",3240,60)

{

Outlier ("General",0.05);

};

R_Date("K-5353 pulses", 3430, 90)

{

Outlier ("General",0.05);

};

R_Date("Hd-6058-5519 grains",3490,80)

{

Outlier ("General",0.05);

};

R_Date("Hd-6059-7967 grains",3140,70)

{

Outlier ("General",0.05);

};

};

Boundary();

};

Sequence()

{

Boundary();

Phase("Mature LBIA/LMIA charcoal from Trianda, Rhodes")

{

R_Date ("DEM-828 charcoal", 3407,25)

{

Outlier("Charcoal",1);

};

R_Date ("DEM-830 charcoal", 3449,21)

{

Outlier("Charcoal",1);

};

R_Date ("DEM-831 charcoal", 3466,23)

{

Outlier("Charcoal",1);

};

};

Boundary();

};

Sequence()

{

Boundary();

Phase("Miletos LMIA bone samples")

{

R_Date("OxA-11954", 3377, 24)

{

Outlier ("General",0.05);

};

R_Date("OxA-11951", 3423, 23)

{

Outlier ("General",0.05);

};

};

Boundary();

};

};

Boundary("Transition LCI to LCI Advanced or Stages 2/3 or VDL");

Sequence("Akrotiri Stages 2/3 Stored or LCI Adv charcoal")

{

Tau_Boundary ("Stages 2/3");

Phase ("Akrotiri secure Stages 2/3 Food Products in Use/Storage or LCI Adv char n=31")

{

R_Date("OxA-1552 Lathyrus sp.", 3390, 65)

{

Outlier ("General",0.05);

};

R_Date("OxA-1555 Lathyrus sp.", 3245, 65)

{

Outlier ("General",0.05);

};

R_Date("OxA-1548 Lathyrus sp.", 3335, 60)

{

Outlier ("General",0.05);

};

R_Date("OxA-1549 Lathyrus sp.", 3460, 80)

{

Outlier ("General",0.05);

};

R_Date("OxA-1550 Lathyrus sp.", 3395, 65)

{

Outlier ("General",0.05);

};

R_Date("OxA-1553 Lathyrus sp.", 3340, 65)

{

Outlier ("General",0.05);

};

R_Date("OxA-1554 Lathyrus sp.", 3280, 65)

{

Outlier ("General",0.05);

};

R_Date("OxA-1556 Hordeum sp.", 3415, 70)

{

Outlier ("General",0.05);

};

R_Date("K-5352 pulses", 3310, 65)

{

Outlier ("General",0.05);

};

//R_Date("K-5353 pulses NOT CLEAR IF 2/3", 3430, 90)

//{

// Outlier ("General",0.05);

//};

R_Date("K-3228 pulses", 3340, 55)

{

Outlier ("General",0.05);

};

R_Date("OxA-11817 ?Lathyrus sp.", 3348, 31)

{

Outlier ("General",0.05);

};

R_Date("OxA-11818 Hordeum sp.", 3367, 33)

{

Outlier ("General",0.05);

};

R_Date("OxA-11820 Hordeum sp.", 3400, 31)

{

Outlier ("General",0.05);

};

R_Date("OxA-11869 Hordeum sp.", 3336, 34)

{

Outlier ("General",0.05);

};

R_Date("OxA-12170 ?Lathyrus sp.", 3336, 28)

{

Outlier ("General",0.05);

};

R_Date("OxA-12171 Hordeum sp.", 3372, 28)

{

Outlier ("General",0.05);

};

R_Date("OxA-12175 Hordeum sp.", 3318, 28)

{

Outlier ("General",0.05);

};

R_Date("OxA-12172 Hordeum sp.", 3321, 32)

{

Outlier ("General",0.05);

};

R_Date("VERA-2756 Hordeum sp.", 3317, 28)

{

Outlier ("General",0.05);

};

R_Date("VERA-2757 ?Lathyrus sp.", 3315, 31)

{

Outlier ("General",0.05);

};

R_Date("VERA-2758 Hordeum sp.", 3339, 28)

{

Outlier ("General",0.05);

};

R_Date("VERA-2757 repeat ?Lathyrus sp.", 3390, 32)

{

Outlier ("General",0.05);

};

R_Date("VERA-2758 repeat Hordeum sp.", 3322, 32)

{

Outlier ("General",0.05);

};

R_Date ("OxA-25176 insect chitin", 3368, 29)

{

Outlier ("General",0.05);

};

Label("Hd data on SL samples from Akrotiri VDL");

R_Date("Hd-7092-6795 peas",3360,60)

{

Outlier ("General",0.05);

};

// R_Date("Hd-6058-5519 grains no context so exclude",3490,80)

//{

// Outlier ("General",0.05);

// };

// R_Date("Hd-6059-7967 grains no context so exclude",3140,70)

// {

// Outlier ("General",0.05);

// };

Label("LCI Advanced or Advanced? or VDL charcoal, Akrotiri, Thera");

R_Date ("DEM-1311 LC I VDL", 3307, 25)

{

Outlier ("General",0.05);

};

R_Date ("DEM-1529 LC I Advanced? VDL", 3281,25)

{

Outlier ("General",0.05);

};

R_Date ("DEM-1607 LC I Advanced VDL", 3228,30)

{

Outlier ("General",0.05);

};

R_Date ("DEM-1624 LC I Advanced VDL", 3360,25)

{

Outlier ("General",0.05);

};

R_Date ("DEM-1615 LC I Advanced? VDL", 3389,25)

{

Outlier ("General",0.05);

};

R_Date("K-4255 twig in pumice so assume LCI Advanced", 3380, 60)

{

Outlier ("General",0.05);

};

};

Boundary("E2/3");

};

Interval("Stage 2/3 to Eruption");

Sequence("Santorini Eruption/Tsunami no Santorini Data and olive in pumice dates")

{

Tau_Boundary ("TnoS+dataset iii");

Phase ("Close TPQs for VDL or even date of VDL")

{

Label("LBIA/LMIA mature and destruction later below Thera tephra, Trianda, Rhodes, so TPQ");

R_Date ("DEM-94 charcoal", 3347,46)

{

Outlier ("General",0.05);

};

R_Date ("DEM-93 charcoal", 3358,48)

{

Outlier ("General",0.05);

};

R_Combine("Trianda short-lived late LMIA twig, pre-Thera-tephra, Quercus sp.")

{

Outlier ("General",0.05);

R_Date("OxA-10643", 3367, 39)

{

Outlier("SSimple",0.05);

};

R_Date("OxA-11884", 3344, 32)

{

Outlier("SSimple",0.05);

};

};

Date("=Felling Miletos WM");

Label("Thera Tsunami Palaikastro");

Label("Palaikastro Promontory");

R_Date("GrA-30336 Cattle bone",3310,35)

{

Outlier ("General",0.05);

};

R_Date("GrA-30339 Cattle bone",3390,35)

{

Outlier ("General",0.05);

};

Label("Bone/jaw, tooth Palaikastro drain with stratified Thera tephra");

R_Date("GrA-28991 goat/sheep bone/jaw",3325,40)

{

Outlier ("General",0.05);

};

R_Date("GrA-29041 goat/sheep bone/jaw",3345,40)

{

Outlier ("General",0.05);

};

R_Date("GrA-29042 tooth",3385,40)

{

Outlier ("General",0.05);

};

Label("Thera Tsunami Çeşme-Bağlararası");

R_Date("OxA-38858 H1a S",3275,17)

{

Outlier ("General",0.05);

};

R_Date("OxA-38881 H1a B",3367,22)

{

Outlier ("General",0.05);

};

R_Date("OxA-38973 H1a C",3318,19)

{

Outlier ("General",0.05);

};

R_Date("OxA-38972 H1a B",3316,20)

{

Outlier ("General",0.05);

};

R_Date("OxA-38857 H1a S",3312,17)

{

Outlier ("General",0.05);

};

R_Date("OxA-38950 H1b C",3384,22)

{

Outlier ("General",0.05);

};

R_Date("D-AMS019172 H1b B",3372,27)

{

Outlier ("General",0.05);

};

R_Date("OxA-38966 H1c C",3297,19)

{

Outlier ("General",0.05);

};

R_Date("D-AMS019173 H1c C",3291,30)

{

Outlier ("General",0.05);

};

Label(“Thera Tsunami Eşençay Delta”);

R_Date("Lyon7920 organic-rich layer",3295,30)

{

Outlier ("General",0.05);

};

Label("Olive branch or tree root samples from Santorini pre-eruption ABA and HS TPQ for eruption");

R_Date("VERA-5614 ABA olive branch",3282,21)

{

Outlier ("General",0.05);

};

R_Date("VERA-5614HS olive branch",3359,33)

{

Outlier ("General",0.05);

};

R_Date("VERA-5615 ABA olive branch", 3280,24)

{

Outlier ("General",0.05);

};

R_Date("VERA-5615HS olive branch", 3321,24)

{

Outlier ("General",0.05);

};

R_Date("VERA-5620 ABA olive branch",3277,25)

{

Outlier ("General",0.05);

};

R_Date("VERA-5620HS olive branch",3345,24)

{

Outlier ("General",0.05);

};

R_Date("VERA-5610 ABA olive branch",3399,25)

{

Outlier ("General",0.05);

};

R_Date("VERA-5610HS olive branch",3342,26)

{

Outlier ("General",0.05);

};

R_Date("VERA-5083 ABA olive branch",3270,36)

{

Outlier ("General",0.05);

};

R_Date("VERA-5083HS olive branch",3326,77)

{

Outlier ("General",0.05);

};

R_Date("VERA-5082 ABA olive branch",3332,38)

{

Outlier ("General",0.05);

};

R_Date("VERA-5082HS olive branch",3369,36)

{

Outlier ("General",0.05);

};

R_Date("VERA-5084 ABA root",3354,32)

{

Outlier ("General",0.05);

};

R_Date("VERA-5084HS root",3368,34)

{

Outlier ("General",0.05);

};

};

Boundary("=TE5");

};

Before("Kolonna Phase L TAQ")

{

Date("=VERA-4630 B");

};

Interval("Interval not represented, Post-Eruption Final LMIB to Earlier LMIB");

Boundary("Start Extant LMIB or LHII Datasets");

Phase("LMIB or LHII")

{

Sequence("LMIB Trianda, Rhodes, charcoal")

{

Boundary();

Phase()

{

R_Date("DEM-90",3258,54)

{

Outlier ("General",0.05);

};

R_Date("DEM-91",3240,35)

{

Outlier ("General",0.05);

};

R_Date("DEM-856",3175,41)

{

Outlier ("General",0.05);

};

R_Date("DEM-829",3171,33)

{

Outlier ("General",0.05);

};

R_Date("DEM-857",3142,52)

{

Outlier ("General",0.05);

};

R_Date("DEM-858",3138,50)

{

Outlier ("General",0.05);

};

Date("Date Estimate for Later/Late? LMIB, Trianda, Rhodes");

};

Boundary();

};

Sequence ("Chania LMIB Destruction")

{

Tau_Boundary ("TC");

Phase ("Chania, charred seeds")

{

R_Date("OxA-2517", 3380, 80)

{

Outlier ("General",0.05);

};

R_Date("OxA-2518", 3340, 80)

{

Outlier ("General",0.05);

};

R_Date("OxA-2646", 3315, 70)

{

Outlier ("General",0.05);

};

R_Date("OxA-2647", 3150, 70)

{

Outlier ("General",0.05);

};

R_Date("OxA-10320", 3208, 26)

{

Outlier ("General",0.05);

};

R_Date("OxA-10321", 3268, 27)

{

Outlier ("General",0.05);

};

R_Date("OxA-10322", 3338, 26)

{

Outlier ("General",0.05);

};

R_Date("OxA-10323", 3253, 25)

{

Outlier ("General",0.05);

};

};

Boundary ("Chania LMIB Destruction, later LMIB");

};

Sequence ("LMIB Late to LMIB Final Destructions")

{

Sequence("Myrtos-Pyrgos (LMIB Late) Destruction")

{

Tau_Boundary ("TMP");

Phase ("Myrtos-Pyrgos, charred seeds")

{

R_Date("OxA-3187", 3230, 70)

{

Outlier ("General",0.05);

};

R_Date("OxA-3188", 3200, 70)

{

Outlier ("General",0.05);

};

R_Date("OxA-3189", 3270, 70)

{

Outlier ("General",0.05);

};

R_Date("OxA-3225", 3160, 80)

{

Outlier ("General",0.05);

};

R_Date("OxA-10324", 3270, 26)

{

Outlier ("General",0.05);

};

R_Date("OxA-10325", 3228, 26)

{

Outlier ("General",0.05);

};

R_Date("OxA-10326", 3227, 25)

{

Outlier ("General",0.05);

};

R_Date("OxA-10411", 3150, 40)

{

Outlier ("General",0.05);

};

};

Boundary ("Myrtos-Pyrgos Destruction, LMIB Late");

};

Sequence("Mochlos (LMIB Final)")

{

Tau_Boundary ("TM");

Phase ("Mochlos Olive stones")

{

R_Date("Beta-85991", 3240, 50)

{

Outlier ("General",0.05);

};

R_Date("Beta-85992", 3180, 40)

{

Outlier ("General",0.05);

};

R_Date("Beta-115890", 3170, 60)

{

Outlier ("General",0.05);

};

R_Date("Beta-129765", 3220, 40)

{

Outlier ("General",0.05);

};

R_Date("Beta-151768", 3270, 40)

{

Outlier ("General",0.05);

};

};

Boundary ("Mochlos Destruction, LMIB Final");

};

};

};

Boundary("LMIB Final or LHII end to LMII");

Phase ("LMII")

{

Sequence("Knossos LMII Destruction short-lived")

{

Tau_Boundary ("T5");

Phase ("Knossos LMII Destruction, charred seeds")

{

R_Date("OxA-2096", 3070, 70)

{

Outlier ("General",0.05);

};

R_Date("OxA-2097", 3190, 65)

{

Outlier ("General",0.05);

};

R_Date("OxA-2098", 3220, 65)

{

Outlier ("General",0.05);

};

R_Date("OxA-11882", 3156, 33)

{

Outlier ("General",0.05);

};

R_Date("OxA-11943", 3148, 23)

{

Outlier ("General",0.05);

};

};

Boundary ("Knossos LMII Destruction");

};

R_Date("OxA-3674 Kommos LMII bone", 3090, 80)

{

Outlier ("General",0.05);

};

};

Before("Beginning LHIIIA")

{

Date("=Begin Kolonna M: LHIIIA");

};

Boundary("End Sequence");

};

Difference("D","TE5","E2/3",LnN(ln(3),ln(2)));

};

Note: the run reported in Fig. 12A, and in Table 4 (left) no GSRO version, used kIterations=3000;

**Model 1 revised to include Sofular Cave signals (Fig. 13)**

The model is as listed for Model 1 (Fig. 9) above but with the addition of the following lines of code inserted where indicated in the runfile above for Model 1

(where reads: //insert Sofular Cave Sequence here – see below):

For Fig. 13A:

Sequence ("Sofular Cave")

{

Boundary();

Phase("Sofular Br and Mo immediate eruption tracers")

{

C_Date("Br",-1621,25);

C_Date("Mo",-1617,25);

Date("=TE5");

};

Boundary();

};

For Fig. 13B:

Sequence ("Sofular Cave")

{

Boundary();

Combine("=TE5")

{

C_Date("Br",-1621,25);

C_Date("Mo",-1617,25);

};

Boundary();

};
