# Supplementary material for: Second Intermediate Period date for the Thera (Santorini) eruption and historical implications
Source: PLoS One. 2022 Sep 20;17(9):e0274835. doi: 10.1371/journal.pone.0274835 (PMC9488803; doi:10.1371/journal.pone.0274835)
Supplement: S1 File — (DOCX) [file pone.0274835.s003.docx]

**File S1.** **Comments on the individual datasets and their modelling.**

Dataset (a): All data are employed. There are no large outliers. One date only is a very marginal ~6% (>5%) outlier: OxA-12303. One date only has a poor OxCal individual Agreement value, OxA-38858 (~42 < 60).

Dataset (b): All data are employed. Only one date, DEM-1607, is a modest outlier at ~13% probability and is also the only date with a (very) poor OxCal individual Agreement value (~8 < 60).

Dataset (c): All data are employed. Only one date is a very marginal ~6% (>5%) outlier, VERA-5610 ABA; no date has a poor OxCal individual Agreement value.

Dataset (d): All data are employed despite some large outliers (down-weighted by the outlier model). Three dates stand out as especially large outliers (regarding this, arbitrarily, as ≥25% outlier probability): P-1697 ~91% outlier probability (date is too recent), P-1888 ~77% outlier probability (date is too recent) and ETH-3315 ~57% outlier probability (date is too old). In all 9 dates have Posterior v. Prior outlier probabilities >5%, but just 6 are greater than 10% probability outliers (additions to the three large outliers already mentioned are: DEM-1607 ~19%, P-2794 ~22%, Hd-6059-7967 ~19%). See also Fig. 4.

Datasets (e) to (h): re-runs of (a) to (d) applying a maximum likely GSRO for the southern Aegean via the added code line:

Delta_R (“Aegean Max. Test”,4,2);

Dataset (i): All data are employed using the site stratigraphy-derived model in [82]. Four data are flagged as minor outliers: Hv-5841 (7% >5%), VERA-4639 (7% >5%), VERA-4638 (9% >5%), VERA-4582 (7% >5%). Three of these samples have individual OxCal Agreement values <60: Hv-5841 (~34), VERA-4639 (~47) and VERA-4638 (~51).

Dataset (j): All data are employed. OxA-11253 (one of two dates on sample K85A/62D/8:83) is, however, a clear outlier (99% >5%). The weighted average for the two dates is also (even down-weighting OxA-11253 almost entirely) a minor outlier (8% >5%), and two other dates are minor outliers: VERA-2638 (8% >5%) and OxA-3429 (10% >5%). None of the dates have poor individual OxCal Agreement values. Removing OxA-11253 and re-running the model achieves better agreement values and no outlier >8%, but the age range/probability determined for the K85A/66B/4:22+23 charred twig – the aim of the model dating an early short/shorter-lived LMIA sample – is almost the same. Fig. 9 thus uses the initial all-data model.

Dataset (k): All the data are employed initially. The part 1 (Shaft Grave 1) set exhibits 3 outliers at ~11% (OxA-20128, date too old), ~12% (OxA-22634, date too recent) and ~26% probability (OxA-20127, date too old). OxA-20127 and OxA-22634 also exhibit OxCal individual Agreement values <60: ~43 and ~8 respectively. If not regarded as acceptable noise—it is a small set (n=6) and a larger population might reduce the evidence of variability—then this might suggest some non-homogeneity in this assemblage, contrary the excavator assumptions. The two oldest dates give non-modelled (95.4%) calibrated age ranges 1932-1749 BCE (most likely 68.3%: 1894-1774 BCE) and 1885-1698 BCE (most likely 68.3% 1876-1747 BCE). These dates are, on any Aegean chronology (whether ‘high’ and especially ‘low’), much too old to be appropriate for mid-LHI (where either a 17^th^ century BCE date for the ‘high’ chronology or a 16^th^ century date for the ‘low’ chronology would be anticipated). They fall before the modelled Middle Helladic to Late Helladic transition (start LHI) from dataset (i) – whereas they should be afterwards. Thus, as proposed also in [91], the Shaft Grave 1 set was re-run excluding these two data (OxA-20127, OxA-20128). This produces a much more coherent result. There is then just one very minor outlier, OxA-22634 at ~6% probability (this date has an individual OxCal Agreement value of ~35 <60). The resultant Phase end Boundary from this re-run (4-date) model is employed in this paper. The part 2 (Shaft Grave 2) set offers a much more coherent grouping than Shaft Grave 1, with just one minor outlier (~7% probability), OxA-20169. This date also has a poor OxCal individual Agreement value of ~38 < 60.

Dataset (l): All data are employed; no outliers are flagged, and no individual OxCal Agreement values are <60.

Model 1: Only three dates are minor outliers. DEM-1607 at ~10% probability, OxA-12303 at ~7% and VERA-5610 ABA at ~6%. None of the other 87 elements in the model are flagged as outliers. Only two dates have poor individual OxCal Agreement values: DEM-1607 at ~5 < 60, and OxA-388858 at ~36 < 60.

Model 2: Citing the results from the no GSRO Model 2 runs, just three elements of the 241 assessed offer larger outliers (arbitrarily treated as >10% probability) as part of Model 2: 65/N001/I2 Ring 3 Bark at ~26% > 5% (also poor OxCal agreement ~29 < 60), with OxA-10312 within this weighted average an 11% > 5% outlier, and DEM-859 at ~63% >5% (also poor OxCal agreement ~1 < 60). No other element (or date within an element – where R_Combine) has an outlier probability >10% (and just one is ~10%: Hd-6059-7967). In all there are 224 elements in Model 2 with the General Outlier or SSimple Outlier model applied (thus excluding 17 elements with the Charcoal Outlier model applied) and 22 have outlier probability >5%, just 3 (noted above) have >10% outlier probability, and the other 19 have 6% (n=10), 7% (n=3), 8% (n=2), 9% (n=3) to 10% (n=1) typical outlier probability. If we reasonably regard those elements with 6% outlier probability as very marginal (and in different model runs a few are sometimes at 5% outlier probability) then in fact only 5.6% of the dataset (12 of 214) are outliers, which is more or less as would be expected at the 95% level. Just 13 elements in Model 2 typically have individual OxCal Agreement values <60. The three critically low values (e.g. arbitrarily assigned as those <20) are DEM-859 (noted above) at ~1, Hd-6059-7967 at ~2 (outlier probability ~10%) and DEM-1607 at ~6 (outlier probability ~8%). Across such a large, complex, model this performance is good.
