# Supplementary figures and images for: Second Intermediate Period date for the Thera (Santorini) eruption and historical implications
Source: PLoS One. 2022 Sep 20;17(9):e0274835. doi: 10.1371/journal.pone.0274835 (PMC9488803; doi:10.1371/journal.pone.0274835)

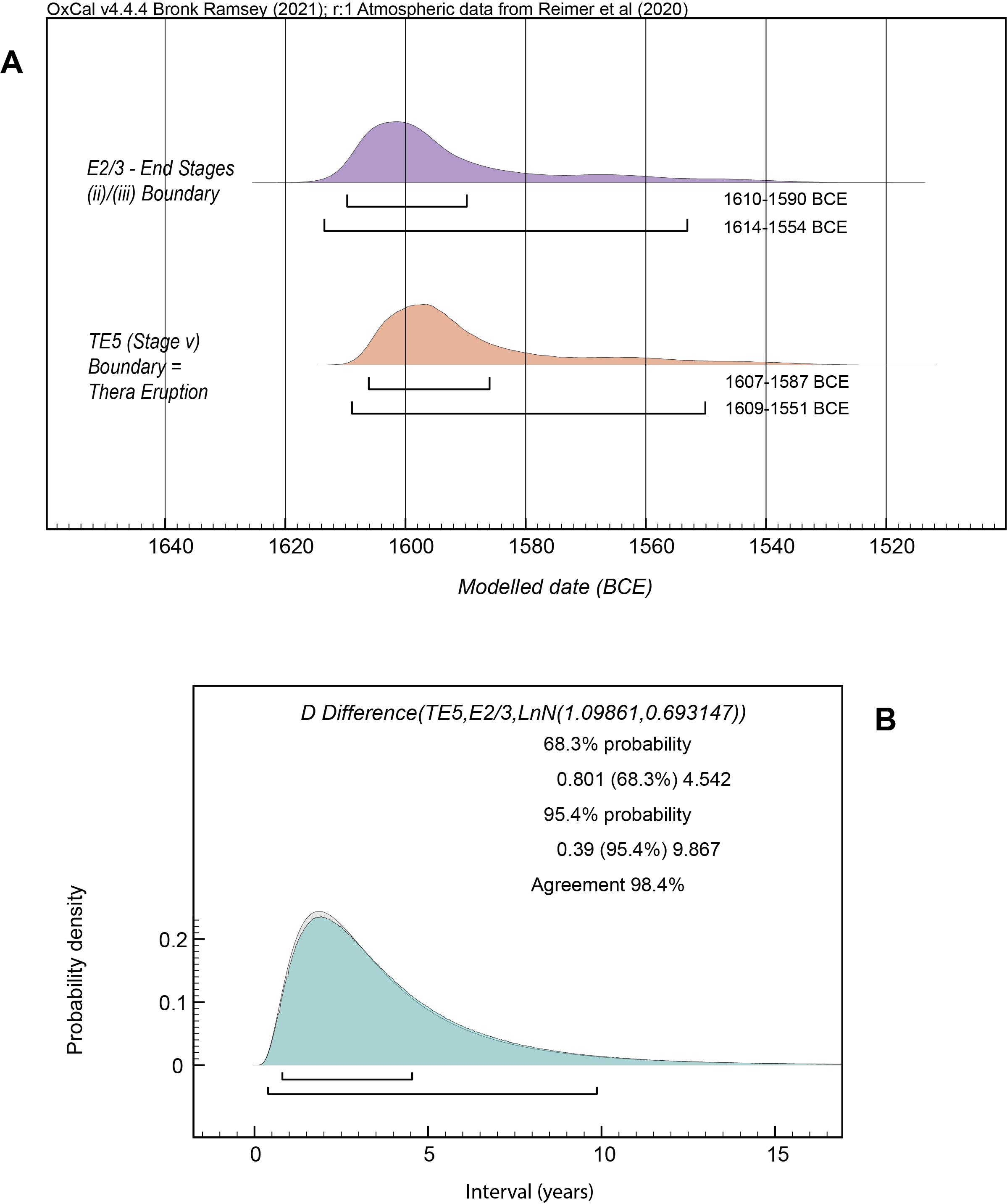

Supplement: S1 Fig — (A) Results for the end of Stages (ii)/(iii) Boundary and the Thera Eruption Boundary from Model 1 run 1 with log-normal, LnN(ln(3),ln(2)), constraint applied (Table 2), detailing the 68.3% and 95.4% hpd calendar age ranges. (B) Modelled posterior (solid, cyan) probability versus the log-normal prior (hollow distribution) for the Difference constraint. (JPG) [file pone.0274835.s005.jpg]

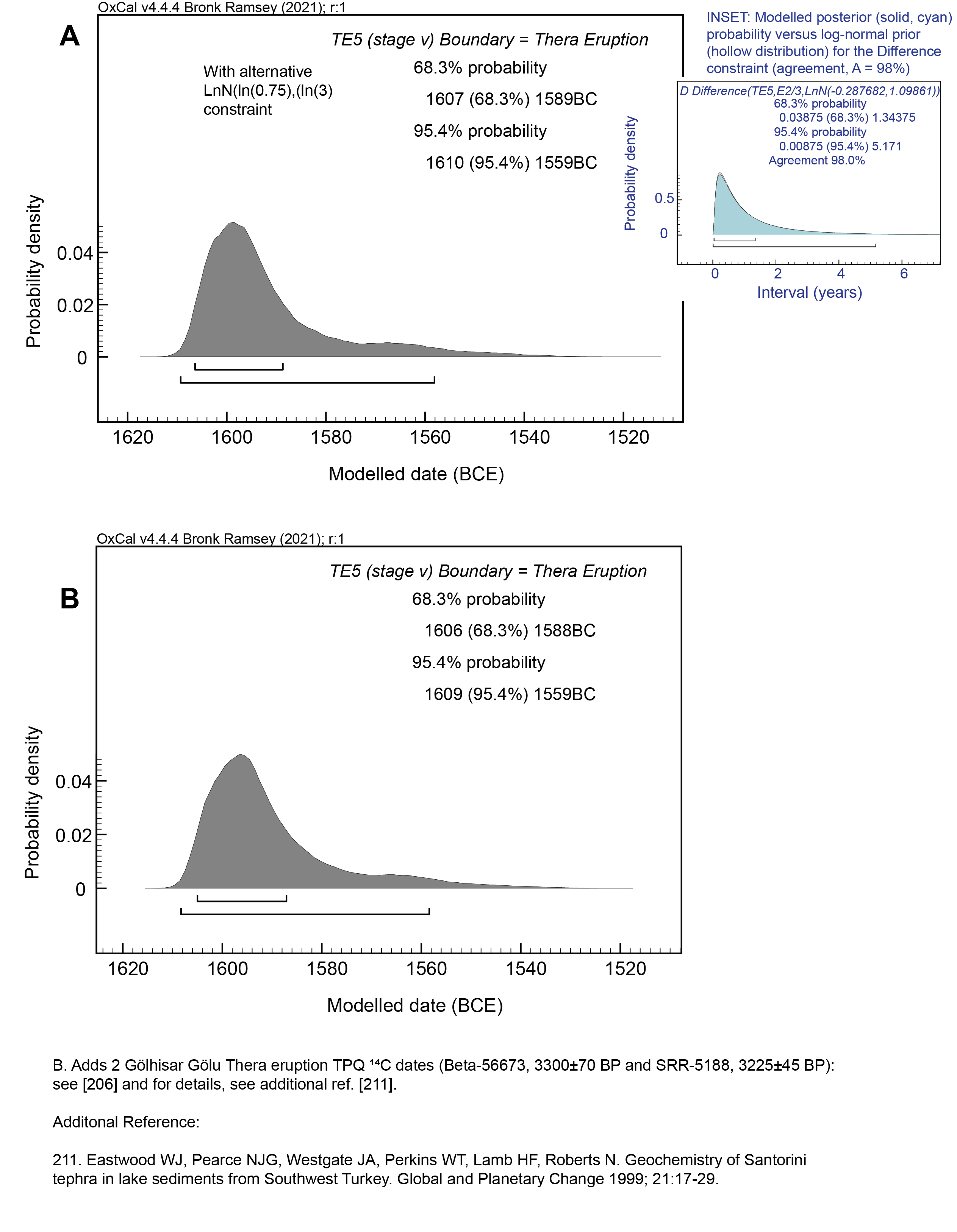

Supplement: S2 Fig — (A) Model 1 (with VERA-4630 TAQ) as in Fig 9 and reported in Table 1 but re-run instead with a more compressed/shorter LnN(ln(0.75),ln(3)) constraint on the Difference query for the time interval between stages (ii)/(iii) and stage (v). The modelled Thera Eruption (stage v) Boundary determined is very similar to the result reported with the slightly looser LnN(ln(3),ln(2)) constraint in the main text (Fig 9 and Table 1). Note: the INSET shows the modelled posterior (solid, cyan) probability versus the log-normal prior (hollow distribution) for this Difference constraint and shows good agreement (98.0%) for this more compressed/shorter constraint also (compare Fig 9B). (B) Model 1 (with VERA-4630 TAQ) as in Fig 9 and reported in Table 1 re-run adding two Thera eruption TPQ 14C dates from Gölhisar Gölu. The modelled Thera Eruption (stage v) Boundary is very similar to that shown in Fig 9 and the values reported for this Boundary from multiple runs of the Fig 9 model in Table 1. (JPG) [file pone.0274835.s006.jpg]

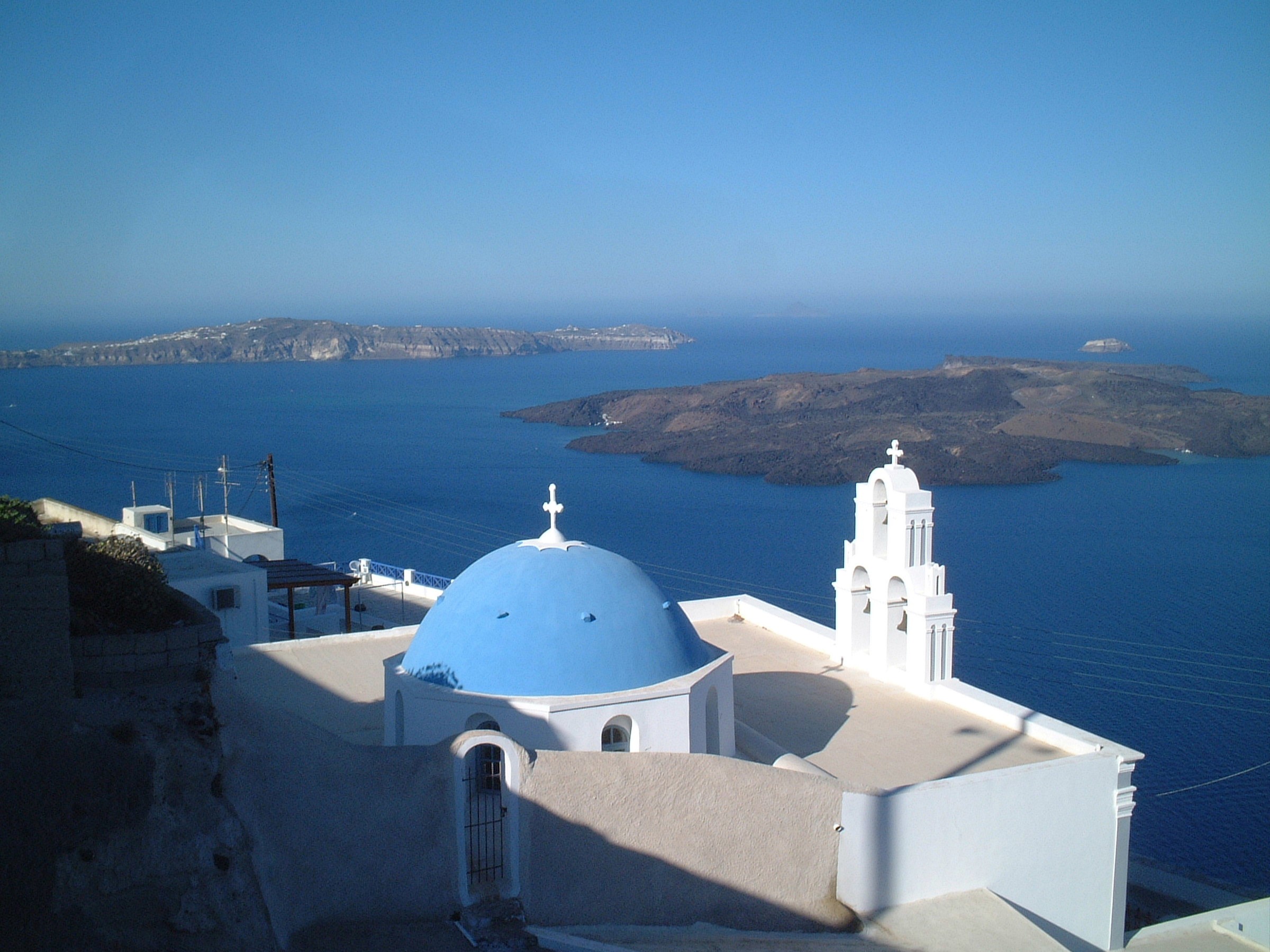

Supplement: S3 Fig — (JPG) [file pone.0274835.s007.jpg]

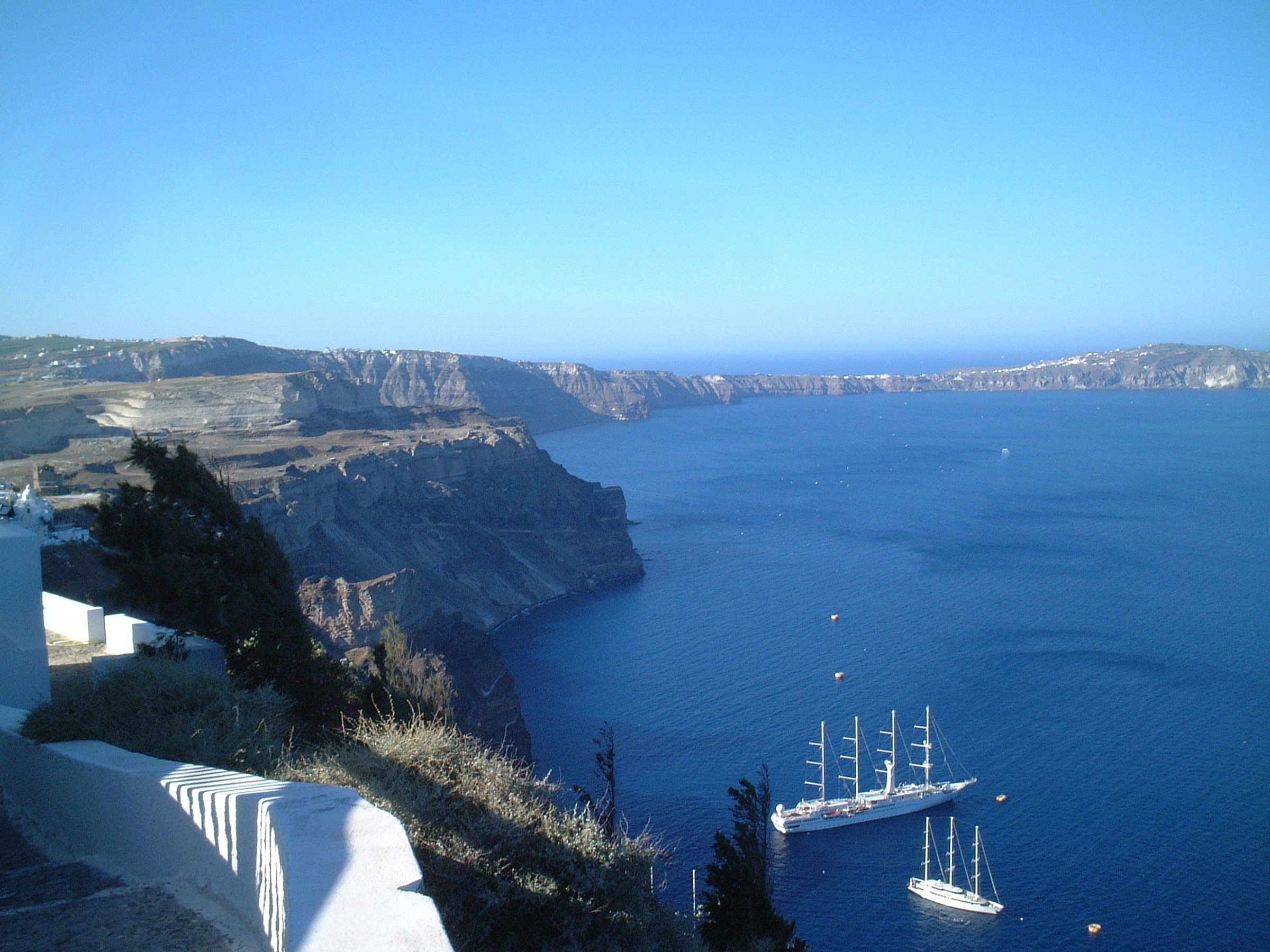

Supplement: S4 Fig — (JPG) [file pone.0274835.s008.jpg]
